# Congrong Shujing Granules ameliorates mitochondrial associated membranes to against MPP^+^-induced neurological damage in the cellular model of Parkinson’s disease

**DOI:** 10.3389/fphar.2025.1509317

**Published:** 2025-05-30

**Authors:** Chutian Zhang, Peizhen Huang, Huiling Cheng, Xinxin Yuan, Mei Zhou, Ying Liu, Ting Liu, Dan Chen, Qian Xu, Jing Cai

**Affiliations:** ^1^ Academy of Integrative Medicine, College of Integrative Medicine, Fujian University of Traditional Chinese Medicine, Fuzhou, Fujian, China; ^2^ Department of Neurology, Hubei Provincial Hospital of Traditional Chinese Medicine, Affiliated Hospital of Hubei University of Traditional Chinese Medicine, Wuhan, Hubei, China; ^3^ The Third Affiliated People’s Hospital of Fujian University of Traditional Chinese Medicine, Fuzhou, Fujian, China; ^4^ College of Pharmacy, Fujian University of Traditional Chinese Medicine, Fuzhou, Fujian, China

**Keywords:** mitochondrial calcium overload, IP3R-VDAC-MCU complex, reactive oxygen species, nerve cell apoptosis, neuroprotection

## Abstract

**Context:**

Congrong-Shujing Granules (CRSJG) are known to protect dopaminergic neurons in Parkinson’s disease (PD), and the mechanism may be related to the improvement of mitochondria-associated membranes (MAMs).

**Objective:**

To investigate the impact of CRSJG-medicated serum on MAMs in the 1-methyl-4-phenylpyridinium (MPP^+^)-induced PD cell model.

**Materials and methods:**

Human neuroblastoma (SH-SY5Y) cells were treated with 1,000 μmol/L MPP^+^ for 24 h, resulting in three experimental groups: MPP^+^, CRSJG, and 2-APB. The MPP^+^ group received blank serum, CRSJG group was treated with CRSJG-medicated serum, and the 2-APB group was given 100 μmol/L 2-APB. An untreated control group was also included. Serum pharmacokinetics for the CRSJG group were analyzed using ultra-performance liquid chromatography-tandem mass spectrometry. Intracellular-Ca^2+^, reactive oxygen species (ROS), mitochondrial membrane potential (MMP), and apoptosis rates were measured using fluorescent probes and flow cytometry. Transmission electron microscopy was employed to examine the morphology of MAMs. Western blot was conducted to identify proteins related to apoptosis and Ca^2+^ transport.

**Results:**

CRSJG-medicated serum identified by pharmacokinetic markers including echinacoside, paeoniflorin, salvianolic acid B, acteoside, and tanshinone IIa. The EC_50_ for MPP^+^-induced reduction in cell proliferation was 1,110 μmol/L. CRSJG-medicated serum, especially at 2.5%, significantly improved cell proliferation after 24 h. The serum effectively mitigated damage within the MAMs region and reduced both the mitochondrial-Ca^2+^ fluorescence intensity and the expression of the IP_3_R-VDAC-MCU complex in MPP^+^-induced neuronal cells. Additionally, it significantly decreased the levels of ROS, the decline in MMP, and apoptosis rates in these cells.

**Discussion and conclusion:**

The findings provide novel insights into the potential of CRSJG in treating neuronal loss in PD.

## Introduction

Parkinson’s disease (PD) affects over six million middle-aged and elderly individuals worldwide (de Lau and Breteler, 2006; Ascherio and Schwarzschild, 2016). The etiology of PD is complex, involving disturbances in Ca^2+^ homeostasis within various cellular compartments, notably the mitochondria and endoplasmic reticulum. These disturbances are considered central to the pathogenesis of the disease (Zaichick et al., 2017; Walters and Usachev, 2023).

The mitochondria and endoplasmic reticulum function as the primary intracellular Ca^2+^ reservoirs and play crucial roles in maintaining Ca^2+^ homeostasis, which is essential for the stability of neuronal function (Xu et al., 2020; Borsche et al., 2021). Mitochondrial associated membranes (MAMs), as a pivotal junction between the mitochondria and endoplasmic reticulum, is involved in facilitating Ca^2+^ transport, mitochondrial autophagy, neuronal apoptosis, and other vital intracellular processes (Barazzuol et al., 2021). Within this interface, the inositol 1,4,5-trisphosphate receptor (IP_3_R) -voltage-dependent anion channel (VDAC) -mitochondrial calcium uniporter (MCU) complex, located in the MAMs region, is instrumental in regulating Ca^2+^ exchange between the mitochondria and endoplasmic reticulum (Lim et al., 2021; Mao et al., 2022). IP_3_R, positioned on the endoplasmic reticulum surface, acts as a Ca^2+^ efflux channel, enabling Ca^2+^ release into the cytosol (Parys and Vervliet, 2020); VDAC, prevalent in the mitochondrial outer membrane, mediates the ingress and egress of ions and metabolites (Benz, 2021); The MCU complex, found in the mitochondrial inner membrane, is paramount for the entry of cytosolic Ca^2+^ into the mitochondria (Yoo, 2022). Exposure to adverse stimuli, including chemical toxins and inflammation, leads to increased expression of proteins in the IP_3_R-VDAC-MCU complex, located between the endoplasmic reticulum and mitochondria. This induces hypertransport of Ca^2+^, disrupting intracellular Ca^2+^ balance, impairing mitochondrial function, and exacerbating the progression of PD (Takada et al., 2017; Erustes et al., 2021).

**FIGURE 1:**
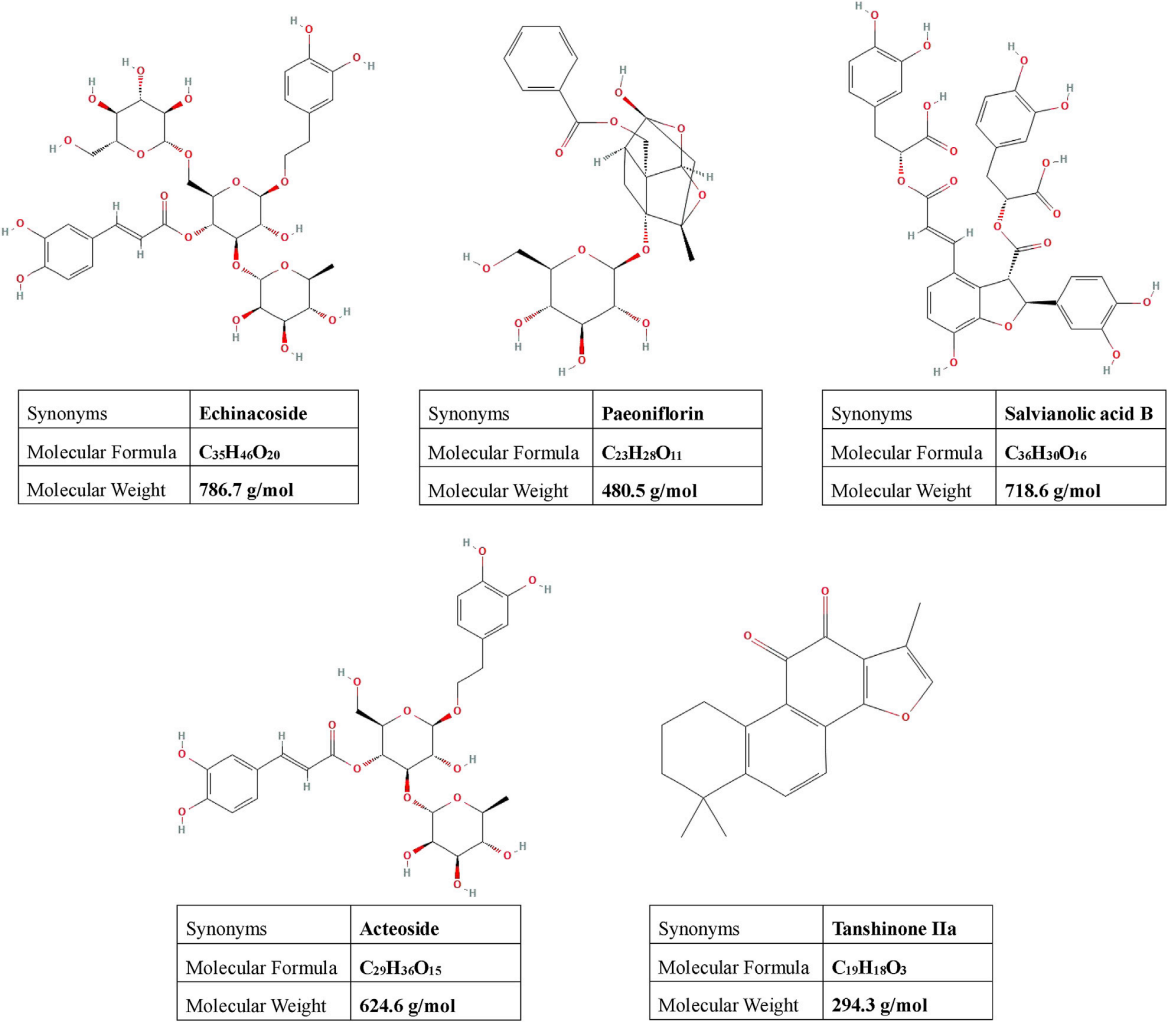
Presents the chemical structures of the primary active compounds identified in CRSJG-medicated serum. The structural information for each compound was obtained from the National Center for Biotechnology Information (NCBI) as follows: echinacoside (PubChem CID: 5281771), paeoniflorin (PubChem CID: 442534), salvianolic acid B (PubChem CID: 6451084), acteoside (PubChem CID: 5281800), and tanshinone IIa (PubChem CID: 164676).

Congrong Shujing Granules (CRSJG), developed by our team, are based on the classical Chinese medicine formula *Dihuang Yinzi*. Previous research has confirmed the neuroprotective efficacy of CRSJG, particularly its effects on mitochondria and endoplasmic reticulum in dopaminergic (DA) neurons (Xu et al., 2020). A variety of bioactive compounds-echinacoside, paeoniflorin, salvianolic acid B, acteoside, and tanshinone IIa (Figure 1 provides the chemical structures of these compounds)-are present in the medicinal plants comprising CRSJG. These compounds were selected for their pharmacological relevance to PD, a neurodegenerative disorder characterized by oxidative stress, mitochondrial dysfunction, calcium signaling, or neuroinflammation (Wu et al., 2019; Subedi and Gaire, 2021; Li et al., 2022; Gong et al., 2024; Han et al., 2024).

However, the effects of CRSJG on intracellular Ca^2+^ homeostasis and the structural and functional integrity of MAMs remain unexplored. Therefore, this study examines the impact of CRSJG-medicated serum on Ca^2+^ homeostasis and MAMs in a 1-methyl-4-phenylpyridinium (MPP^+^)-induced Parkinson’s disease cell model, elucidating the underlying mechanisms of CRSJG’s protective role in DA neurons.

## Materials and methods

### Reagents

MPP^+^ was obtained from Macklin, China; 2-ABP was obtained from APExBIO, United States; reference substance of Rutin was obtained from Yuanye, China; reference substance of echinacoside was obtained from Alfa, China; reference substance of paeoniflorin, salvianolic acid B, acteoside and tanshinone IIa were obtained from Dilger, China; CCK-8, Fluo-3-AM fluorescent probe, Reactive oxygen species (ROS) assay kit, and JC-1 mitochondrial membrane potential (MMP) assay kit were obtained from Beyotime, China; Rhod-2-AM cell permeable calcium ion fluorescence probe Yeasen, China; IP_3_R, VDAC, GRP75, Cyt-C, Bax, Bcl-2, Caspase-3, and Caspase-9 antibodies were obtained from Proteintech, China; Cell apoptosis kit and MCU antibodies were obtained from Abbkine, China; MCU antibodies and BCA Protein Assay Kit were obtained from Boster, China; DMEM medium was obtained from Gibco, United States; Origin fetal bovine serum (FBS) was obtained from MRC, United States.

### Animals

The study has been approved by the ethics committee of Fujian University of Traditional Chinese Medicine (No. 2020063), and all operations were carried out in adherence to the Guide for the Care and Use of Laboratory Animals. Forty Sprague-Dawley rats (SD rats), SPF grade, were obtained from Slac Laboratory (certificate 20170005022867). Rats were raised in an SPF-grade animal laboratory with a relative humidity of 60%, 25°C, and a 12-h light/dark cycle.

### Extraction of Congrong Shujing Granules

CRSJG is a new traditional Chinese medicine developed by *the Creation of Major New Drugs for Major National Science and Technology Projects* (No.2019ZX09301154), and it was obtained from *the Third Affiliated People’s Hospital of Fujian University of Traditional Chinese Medicine*. The compound ingredients include *Cistanche deserticola* Y.C. Ma (Rou congrong, Orobanchaceae), *Polygonatum kingianum* Collett & Hemsl. (Jiu huangjing, Asparagaceae), *Salvia miltiorrhiza* Bunge (Dan shen, Lamiaceae), *Paeonia lactiflora* Pall. (Chi shao, Paeoniaceae), and *Paeonia suffruticosa* Andrews (Mu danpi, Paeoniaceae), the dosage ratio of each component is 6:12:15:12:10, and related details are summarized in Table 1 (Ou et al., 2022).

The total amount of the five materials was 275 g, including *Cistanche deserticola* (30 g), *Polygonatum kingianum* (60 g), *Salvia miltiorrhiza* (75 g), *Paeonia lactiflora* (60 g), and *Paeonia suffruticosa* (50 g). We added 2,750 mL and 2,200 mL of water sequentially, and decocted the mixture twice at 100°C for 1 h each time. After filtering the residue, 5% dextrin was added, mixed thoroughly, and dried to yield CRSJG.

**TABLE 1:** Compound ingredients of Congrong Shujing granules.

Latin name	Family	Chinese name	Medicinal parts	Origin	Percentage
*Cistanche deserticola* Y.C. Ma	Orobanchaceae	Rou congrong	root and rhizome pulp	Neimenggu	11%
*Polygonatum kingianum* Collett & Hemsl.	Asparagaceae	Jiu huangjing	root	Hebei	22%
*Salvia miltiorrhiza* Bunge	Lamiaceae	Dan shen	root	Shandong	27%
*Paeonia lactiflora* Pall.	Paeoniaceae	Chi shao	root	Neimenggu	22%
*Paeonia suffruticosa* Andrews	Paeoniaceae	Mu danpi	root bark	Anhui	18%

### Preparation of CRSJG-medicated serum

After 1 week of adaptive feeding, rats were randomly divided into drug-containing serum group (n = 14) and blank serum group (n = 14). The CRSJG-medicated serum group received CRSJG via oral gavage at a dose of 10 g/(kg·d) for seven consecutive days, while the blank serum group received an equal volume of normal saline. Following the administration period, all rats were fasted for 12 h before anesthesia but were provided unrestricted access to water. All rats were injected with a dose of urethane (1,000 mg/kg), and blood was collected from the abdominal aorta. Rat-serum was collected, inactivated, and stored at −80°C.

### Pharmacokinetics of main components of CRSJG-medicated serum determined by ultra-performance liquid chromatography-tandem mass spectrometry

Rats were randomly selected from the drug-containing serum group (n = 6) and the blank serum group (n = 6). These rats were fasted for 12 h but allowed free access to water. They received 10 g/(kg·d) of CRSJG or an equivalent dose of normal saline via intragastric administration. Orbital blood samples were collected at 0.083, 0.167, 0.25, 0.5, 0.75, 1, 2, 4, 6, 8, 12, 24, and 36 h post-administration. Reference substances were weighed and dissolved in methanol by ultrasonication to prepare the reference solutions. The concentrations of these solutions were: echinacoside 126,819 ng/mL, paeoniflorin 2,657 ng/mL, salvianolic acid B 2,563 ng/mL, acteoside 603.5 ng/mL, tanshinone IIa 137.1 ng/mL, and rutin 457.5 ng/mL.

Chromatographic analysis: Chromatographic separation was performed using an ACQUITY UPLC BEH C18 column (2.1 mm × 100 mm, 1.7 μmol/L). The mobile phase consisted of acetonitrile (A) and 0.1% formic acid in water (B), with a gradient elution at a flow rate of 0.3 mL/min. The column temperature was maintained at 40 °C, and the injection volume was 2 μL. The gradient profile was as follows: from 0.0 to 1.5 min, 14%–30% A; from 1.5 to 3.0 min, 30%–70% A; from 3.0 to 5.0 min, 70%–90% A; from 5.0 to 6.0 min, held at 90% A; from 6.0 to 6.1 min, 90%–14% A; and from 6.1 to 7.0 min, held at 14% A.

Mass spectrometry: electrospray ionization source (ESI); Capillary ionization voltage 3.5 kV; Ion source temperature 110°C; Desolvent gas N_2_, volume flow rate 800 L/h, 500°C; Back-blowing gas N_2_, volume flow 50 L/h; Collision gas Ar, volume flow 0.13 mL/min; Mass spectrum data acquisition and processing by Masslynx4.1 software, multiple reaction monitoring (MRM) scanning, and related mass spectrum conditions are shown in Table 2.

**TABLE 2:** Mass spectrometry conditions.

Component	Mass spectrometry conditions				
	ESI	Parent (m/z)	Daughter (m/z)	Cone (V)	Collision (V)
Echinacoside	—	785.20	461.10	50	25
Paeoniflorin	—	525.16	121.02	28	25
Salvianolic acid B	+	521.07	295.05	40	18
Acteoside	—	623.10	161.10	46	37
Tanshinone II A	+	295.13	249.09	30	22
Rutin	—	609.14	300.02	35	35

Calibration standards were prepared by diluting methanol stock to yield a series of gradient reference solutions. 200 μL of each standard was dried under a gentle nitrogen stream at 45°C, then reconstituted in 200 µL of the blank serum. Ultra-performance liquid chromatography-tandem mass spectrometry (UPLC-MS/MS) was used to analyze these samples, construct standard curves, and assess linearity. To validate the method, three quality-control levels (low, medium, high) of internal-standard mixtures were spiked into blank serum, and the following performance characteristics were evaluated: precision tests, recovery assays, extraction recovery assays, matrix effect analyses, and sample stability. Pharmacokinetic parameters were determined in CRSJG-medicated serum collected at defined intervals post-intragastric administration, and were calculated using DAS 2.0 software with a non-compartmental model.

### Cell culture

Human neuroblastoma cell line (SH-SY5Y) was obtained from *Shanghai Cell Bank, Chinese Academy of Sciences*. Cells were cultured in DMEM high-glucose medium (10% FBS, 100 U/mL penicillin and 100 μg/mL streptomycin) at saturated humidity, 5% CO_2_, and 37°C.

### Construction of MPP^+^-induced neurological damage model

Cell Counting Kit-8 (CCK-8) Assay was used to screen the induction concentration of MPP^+^. MPP^+^ (0, 250, 500, 750, 1,000, 1,500, 2000 μmol/L) was added to the cells, and a blank control group (without cells or drugs) was included to account for background absorbance. After 24 h of intervention, 10 μL CCK-8 (Beyotime, China) was added, incubated for 2 h, and the optical density (OD) at 450 nm was measured using a microplate reader (Bio-TEKEL, United States).

### Screening of CRSJG-medicated serum concentration

For CRSJG-medicated serum intervention, SH-SY5Y cells were pre-treated with 1,000 μmol/L MPP^+^ for 24 h, followed by exposure to 0%, 2.5%, 5%, 10%, 15%, or 20% CRSJG-medicated serum. Three zero adjustment holes (without cells or drugs) were included. At 12 h, 24 h, 36 h, and 48 h post-treatment, 10 μL CCK-8 solution was added to each well, followed by 2 h of incubation under the same conditions. The OD at 450 nm was recorded using a microplate reader. OD values of the Background absorbance controls (wells containing only the medium and CCK-8 reagent) were subtracted from experimental readings to ensure data accuracy.

### Experimental grouping and drug intervention

SH-SY5Y cells were divided into MPP^+^ group, 2-aminoethoxydiphenyl borate (2-APB) group, CRSJG group with 1,000 μmol/L MPP^+^, control group without MPP^+^. 2-APB is a classic cell-penetrable inhibitor of IP_3_R that can block IP_3_-mediated intracellular calcium release, and was used as a positive control drug in our study.

SH-SY5Y cells were seeded at a density of 1 × 10^5^ cells/mL in culture dishes, and intervention begins when cells density reached 70–80 percent. CRSJG-medicated serum was added to CRSJG group, and blank serum was added to control group, MPP^+^ group, and 2-APB group. We ensured that the total serum concentration (CRSJG-medicated serum or blank serum + FBS) at 10% in all groups. The cells were cultured for 24 h with 5% CO_2_, 37°C. The intervention system of each group was as follows: Control group (7.5% FBS +2.5% blank serum); MPP^+^ group (7.5% FBS +1000 μmol/L MPP^+^ + 2.5% blank serum); 2-APB group (7.5% FBS +1000 μmol/L MPP^+^ + 2.5% blank serum +100 μmol/L 2-APB); CRSJG group (7.5% FBS +1000 μmol/L MPP^+^ + 2.5% CRSJG-medicated serum). MPP^+^ and 2-APB were dissolved directly in high-glucose DMEM to achieve the required working concentrations.

### Intracellular Ca^2+^ levels analysis

The intracellular Ca^2+^ levels were detected by fluorescence probe and flow cytometry. After induction with MPP^+^ and subsequent treatment with either CRSJG-medicated serum or blank serum, SH-SY5Y cells were incubated with 300 μL of the mitochondrial Ca^2+^-sensitive fluorescent dye Rhod-2 AM (Yeasen, China; experimental concentration 10 μmol/L) and 300 μL of the cytoplasmic Ca^2+^-sensitive fluorescent dye Fluo-3 AM (Beyotime, China; experimental concentration 10 μmol/L) for 30 min at 37°C in the dark. Following incubation, cells were washed twice with PBS, and fluorescence images were acquired using a fluorescence microscope (Nikon, Japan). The mean fluorescence intensity (MFI) was quantified using *ImageJ* software.

For flow cytometry analysis of intracellular Ca^2+^ Levels, SH-SY5Y cells stained using the same protocol were gently dissociated into a single-cell suspension using a pipette, centrifuged at 300 g for 3 min, and then resuspended in 200 μL of PBS. The average fluorescence intensity of FITC (for Fluo-3 AM) or PE (for Rhod-2 AM) channels was recorded by flow cytometry (Beckman Coulter, United States).

### Reactive oxygen species measurement

Intracellular ROS levels in SH-SY5Y cells were measured using ROS assay kit (Beyotime, China), following the manufacturer’s instructions. SH-SY5Y cells were collected and prepared as a single-cell suspension. For the positive control group, 1 μL of Rosup was added and incubated for 20 min to induce ROS production. The negative control group received no treatment. All other experimental groups followed the previously described intervention protocol. After drug treatment, cells were incubated with 10 μmol/L DCFH-DA diluted in PBS at 37°C for 30 min in the dark. Following incubation, cells were washed three times with PBS to remove excess dye. Intracellular ROS levels were then quantified using flow cytometry (FITC). Detailed gating strategies are shown in Annex 1.

### Mitochondrial membrane potential assessment

MMP was evaluated using a JC-1 assay kit (Beyotime, China) in accordance with the manufacturer’s protocol. SH-SY5Y cells were collected and prepared as a single-cell suspension. The positive control group was treated with 10 μmol/L CCCP for 20 min. The negative control group remained untreated. Other groups received the same interventions as described previously. Following treatment, cells were incubated with JC-1 working solution (1×) at 37°C for 30 min in the dark. Cells were then washed twice with JC-1 buffer and analyzed by flow cytometry. JC-1 aggregates (high MMP) were detected in the PE channel, while JC-1 monomers (low MMP) were measured in the FITC channel. Detailed gating strategies are shown in Annex 1.

### Cell apoptosis detection

Cell apoptosis was assessed using an Annexin V-FITC/PI Apoptosis Detection Kit (Abbkine, China). After treatment, SH-SY5Y cells were harvested, washed twice with cold PBS, and resuspended in 500 μL of binding buffer. Cells were then stained with 5 μL Annexin V-FITC and 10 μL PI and incubated at 37°C for 30 min in the dark. Single-staining controls were prepared using Annexin V-FITC or PI only, and untreated cells served as the negative control. Samples were analyzed by flow cytometry, and the percentages of early and late apoptotic cells were quantified. Detailed gating strategies are shown in Annex 1.

### Western blot analysis

Western blotting was performed to detect proteins related to apoptosis and Ca^2+^ transport. SH-SY5Y cells were trypsinized with 0.25% trypsin-EDTA, collected by centrifugation at 1,200 rpm for 5 min at 4°C, and washed twice with ice-cold PBS. The cell pellet was resuspended in RIPA lysis buffer (containing 1% PMSF and protease/phosphatase inhibitors), followed by incubation on ice for 15 min with intermittent vortexing. Lysates were then centrifuged at 12,000 rpm for 15 min at 4°C, and the supernatant containing total protein was collected.

Protein concentration was determined using the BCA Protein Assay Kit (Boster, China). Equal amounts of protein were mixed with 1× SDS loading buffer and denatured at 95°C for 10 min. Samples were separated on a 10% SDS-PAGE gel and transferred onto 0.45 μm PVDF membranes (Millipore, United States). Membranes were blocked with 5% non-fat milk in PBS for 2 h at room temperature, followed by incubation with primary antibodies (IP3R, VDAC, GRP75, Cyt-C, Bax, Bcl-2, Caspase-3, and Caspase-9 antibodies were obtained from Proteintech, China); MCU antibodies were obtained from Abbkine, China; Dilution Ratio 1:1,000) overnight at 4°C with gentle shaking.

After washing, membranes were incubated with HRP-conjugated secondary antibodies (Proteintech, China; Dilution Ratio 1:10,000) for 1 h at room temperature. Protein bands were detected using an enhanced chemiluminescence (ECL) detection system and analyzed using *ImageLab* software (Bio-rad, United States).

### Transmission electron microscope

SH-SY5Y cells cultured in each group were fixed with 2.5% glutaraldehyde for 5 min. Cells were scraped off with the cell scraper, centrifuged for 15 min, 2000 rpm, and then covered with electron microscopy fixing solution, so that the cells were completely suspended in the fixing solution, and fixed for 30 min. After dehydrating with acetone, samples were embedded and sectioned into ultrathin slices, which were stained with lead citrate for 10 min and uranium acetate for 30 min. After washing and drying, the stained cells samples were scanned and observed by transmission electron microscope (Hitach, Japen).

### Statistical analysis

All experimental data were processed and analyzed by SPSS 24.0, and presented as mean ± standard deviation. The Kolmogorov-Smirnov (K-S) test was used to assess the normality of data distribution. If the data conformed to a normal distribution, One-way analysis of variance (ANOVA) was performed to compare differences among groups; the Least Significant Difference (LSD) method was applied when variances were homogeneous; the Games-Howell method was used when variances were heterogeneous. If the data did not follow a normal distribution, the Kruskal-Wallis test (non-parametric independent sample rank-sum test) was used instead. The test level was α = 0.05, and *p* < 0.05. Finally, GraphPad Prism version 8.0.2 (263) was used to complete each experimental statistical chart.

## Results

### Chemical composition of CRSJG-medicated serum

**FIGURE 2:**
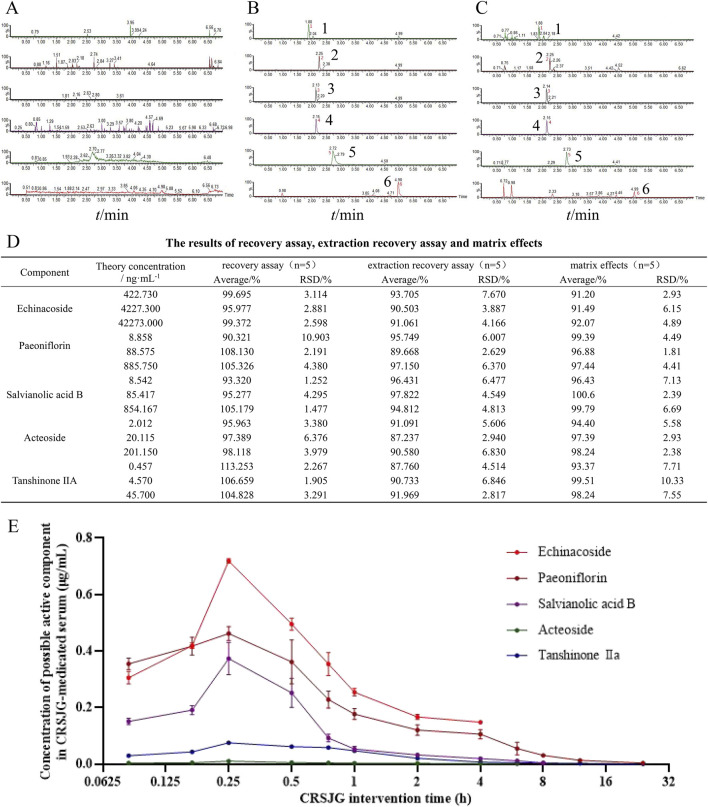
Chemical composition of CRSJG-medicated serum. **(A–C)** Specificity test of the main components of CRSJG-medicated serum and rutin, **(A)** Blank serum; **(B)** Mixed standard solution of six components in blank serum; **(C)** CRSJG-medicated serum; 1. echinacoside; 2. acteoside; 3. internal standard (rutin); 4. paeoniflorin; 5. salvianolic acid B; 6. tanshinone Ⅱ A. **(D)** The results of recovery assay, extraction recovery assay and matrix effects; **(E)** Pharmacokinetics of main components of CRSJG-medicated serum.

Methodological analysis demonstrated that the chromatographic peaks of the primary constituents and rutin were distinctly separated, with no interference from endogenous substances (Figures 2A–C). The recovery and extraction assays for each main component and rutin met established standards. Additionally, rat serum exhibited no significant interference with the primary components of CRSJG and rutin (Figure 2D). These findings comply with the criteria for biological sample analysis. Pharmacokinetic profiling indicated the presence of CRSJG’s key markers - echinacoside, paeoniflorin, salvianolic acid B, acteoside, and tanshinone IIa,- in CRSJG-medicated serum. The mean quantitative results showed that the plasma concentration of the drug peaked at 0.25 h (15 min) post-administration: echinacoside at 718.7 ng/mL, paeoniflorin at 462.0 ng/mL, salvianolic acid B at 373.5 ng/mL, acteoside at 10.61 ng/mL, and tanshinone IIa at 75.24 ng/mL (Figure 2E). The detailed results of the pharmacokinetic parameters of CRSJG-medicated serum are listed in the supplementary figure.

### CRSJG-medicated serum prevented MPP^+^-induced cell death

**FIGURE 3:**
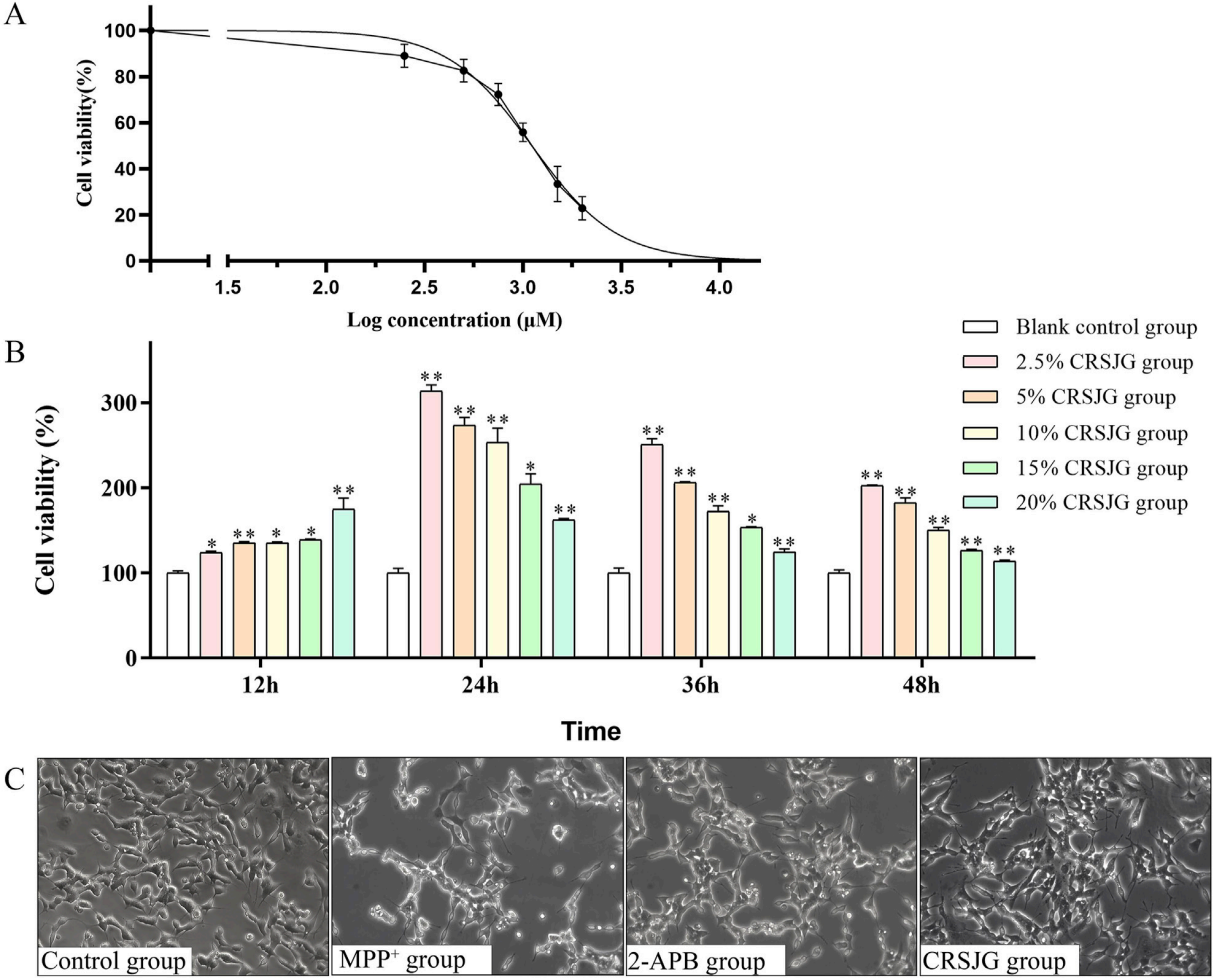
CRSJG-medicated serum prevented MPP^+^-induced cell death. **(A)** Cells were exposed to different concentration gradients of MPP^+^ in the range of 0–2000 μmol/L, and MPP^+^ EC_50_ was calculated using GraphPad Prism version 8.0.2 (263); **(B)** Protective effect of different concentrations of CRSJG-medicated serum on 1,000 μmol/L MPP^+^ induced cells at 12 h, 24 h, 36 h and 48 h; **(C)** After treating with 1,000 μmol/L MPP^+^ -induced SH-SY5Y cells with 2.5% CRSJG-medicated serum or 100 μmol/L 2-APB for 24 h, the cell morphology was observed by inverted fluorescence microscopy (×200). Results are showed as mean ± standard deviation (SD) from three independent experiments. M: * P < 0.05, ** P < 0.01 vs. 0 μmol/L group or Blank control group.

We investigated the impact of MPP^+^ on cell proliferation independently. The findings indicated that all tested concentrations of MPP^+^ significantly decreased cell proliferation, with an EC_50_ value of 1,110 μmol/L (Figure 3A). Therefore, 1,000 μmol/L MPP^+^, being closest to the EC_50_ value, was selected for subsequent experiments. Subsequently, we applied varying concentrations of CRSJG-medicated serum to MPP^+^ induced cells over different durations. Compared to the blank control group, which received blank serum intervention, any concentration of CRSJG-medicated serum was found to effectively mitigate the MPP^+^ induced reduction in cell proliferation rate after an intervention exceeding 12 h. Notably, a 2.5% CRSJG-medicated serum demonstrated the most significant enhancement in proliferation at 24 h of intervention, thereby establishing the most optimal treatment condition (Figures 3B,C). This criterion was therefore chosen for application in further experiments.

### CRSJG regulates intracellular Ca^2+^ homeostasis and alleviates mitochondrial Ca^2+^ overload caused by MPP^+^

**FIGURE 4:**
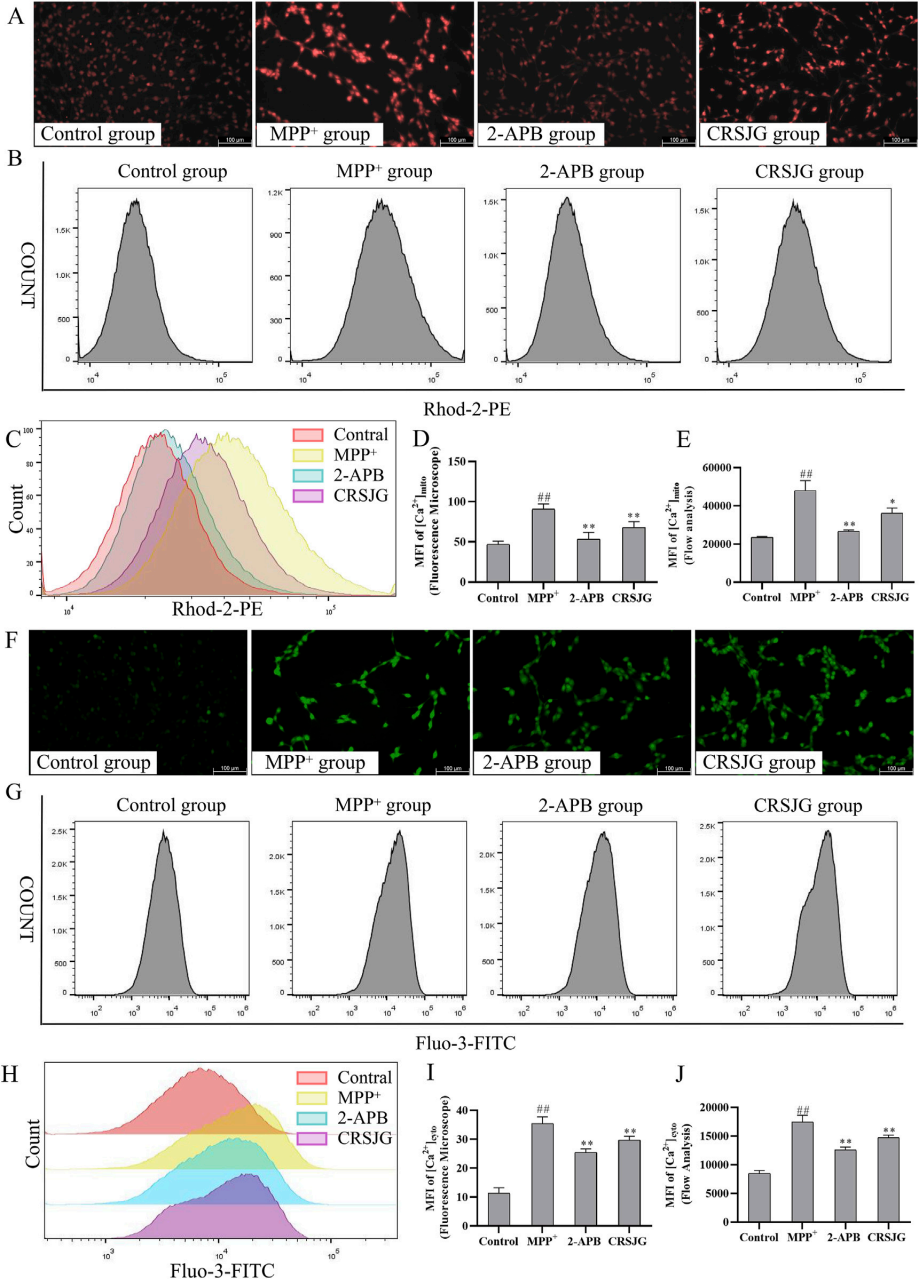
CRSJG regulates intracellular Ca^2+^ homeostasis to relieve mitochondrial Ca^2+^ overload. After treating with 1,000 μmol/L MPP^+^ -induced SH-SY5Y cells with 2.5% CRSJG-medicated serum or 100 μmol/L 2-APB for 24 h, **(A,D)** Rhod-2 fluorescence probe was used to detect the mito-Ca^2+^ levels in each group (×200), and the quantitative results of MFI; **(B,C,E)** Flow cytometry was used to detect the mito-Ca^2+^ levels, and the quantitative results of MFI; **(F,I)** Fluo-3 fluorescence probe was used to detect the cyto-Ca^2+^ levels in each group (×200), and the quantitative results of MFI; **(G,H,J)** Flow cytometry was used to detect the cyto-Ca^2+^ levels, and the quantitative results of MFI. Results are showed as mean ± standard deviation (SD), of which all results are from four independent experiments. M: ##*P* < 0.01 vs. control group; * *P* < 0.05, ** *P* < 0.01 vs. MPP^+^ group.

We respectively employed Rhod-2 and Fluo-3 fluorescence probes to measure mitochondrial-Ca^2+^ (mito-Ca^2+^) and cytoplasmic-Ca^2+^ (cyto-Ca^2+^) levels respectively, followed by a quantitative analysis of mean fluorescence intensity (MFI). Both fluorescence microscopy and flow cytometry analyses revealed that CRSJG effectively mitigated mito-Ca^2+^ aggregation in MPP^+^ induced nerve cells (Figures 4A–E), while maintaining cyto-Ca^2+^ levels at a reduced level (Figures 4F–J). The trends observed in mito-Ca^2+^ and cyto-Ca^2+^ levels following CRSJG intervention paralleled those seen with 2-APB, an inhibitor of intracellular Ca^2+^ transport, albeit with less pronounced effects than 2-APB.

### CRSJG improved the mitochondrial membrane potential reduction and ROS level increase caused by MPP^+^

**FIGURE 5:**
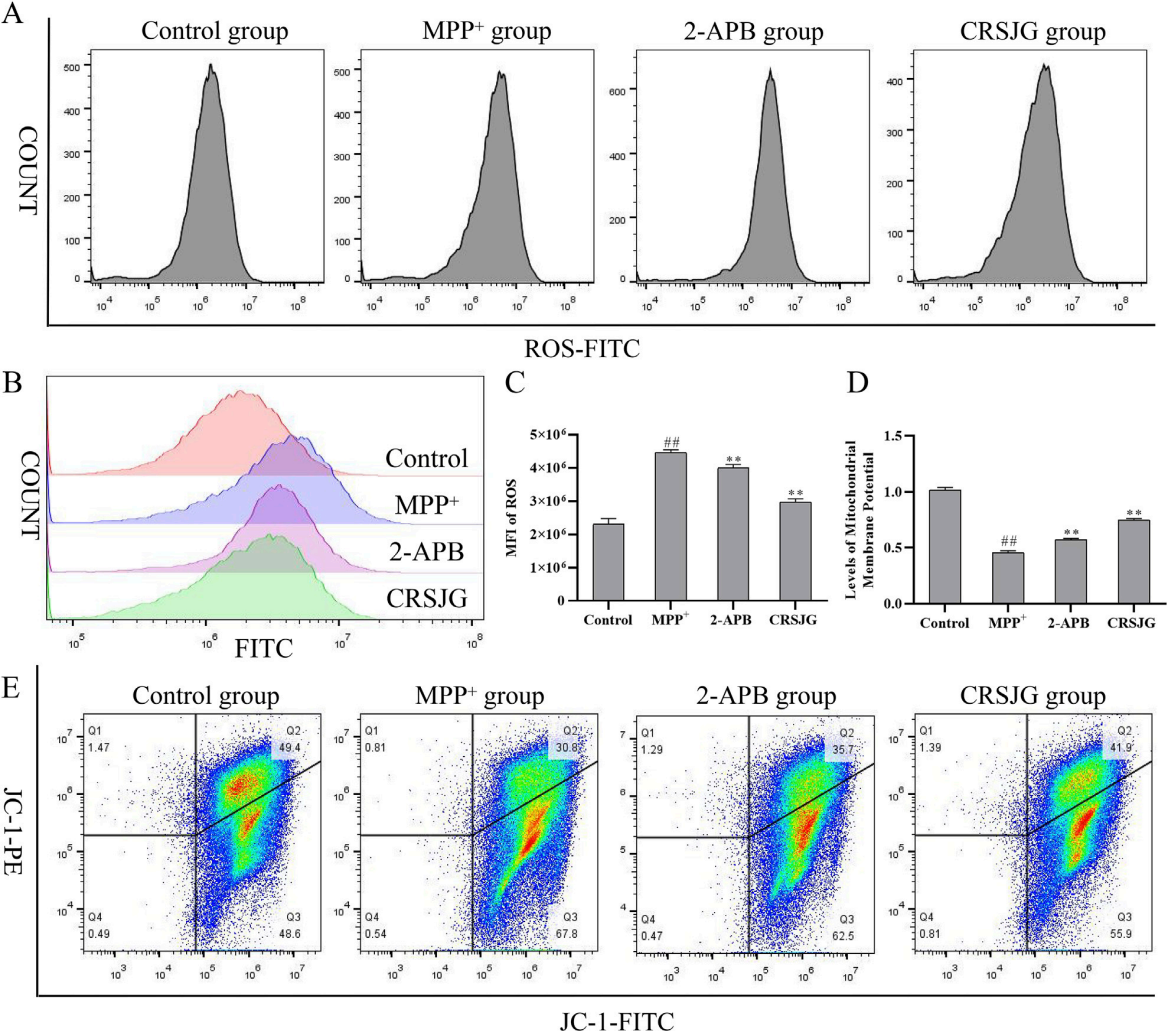
CRSJG improved the mitochondrial membrane potential reduction and ROS increase caused by MPP^+^. After treating with 1,000 μmol/L MPP^+^ -induced SH-SY5Y cells with 2.5% CRSJG-medicated serum or 100 μmol/L 2-APB for 24 h, **(A–C)** Flow cytometry was used to detect ROS levels, and the quantitative results of MFI; **(D,E)** Flow cytometry was used to detect MMP. Results are showed as mean ± standard deviation (SD), of which all results are from four independent experiments. M: ##*P* < 0.01 vs. control group; * *P* < 0.05, ** *P* < 0.01 vs. MPP^+^ group.

We assessed ROS level and MMP in MPP^+^ induced nerve cells using flow cytometry. ROS level in these cells were significantly elevated due to MPP^+^ induction but were effectively normalized upon CRSJG treatment (Figures 5A–C). Concurrently, both CRSJG and 2-APB significantly mitigated the reduction in MMP caused by MPP^+^ (Figures 5D,E).

### CRSJG protects nerve cells from MPP^+^ induced apoptosis

**FIGURE 6:**
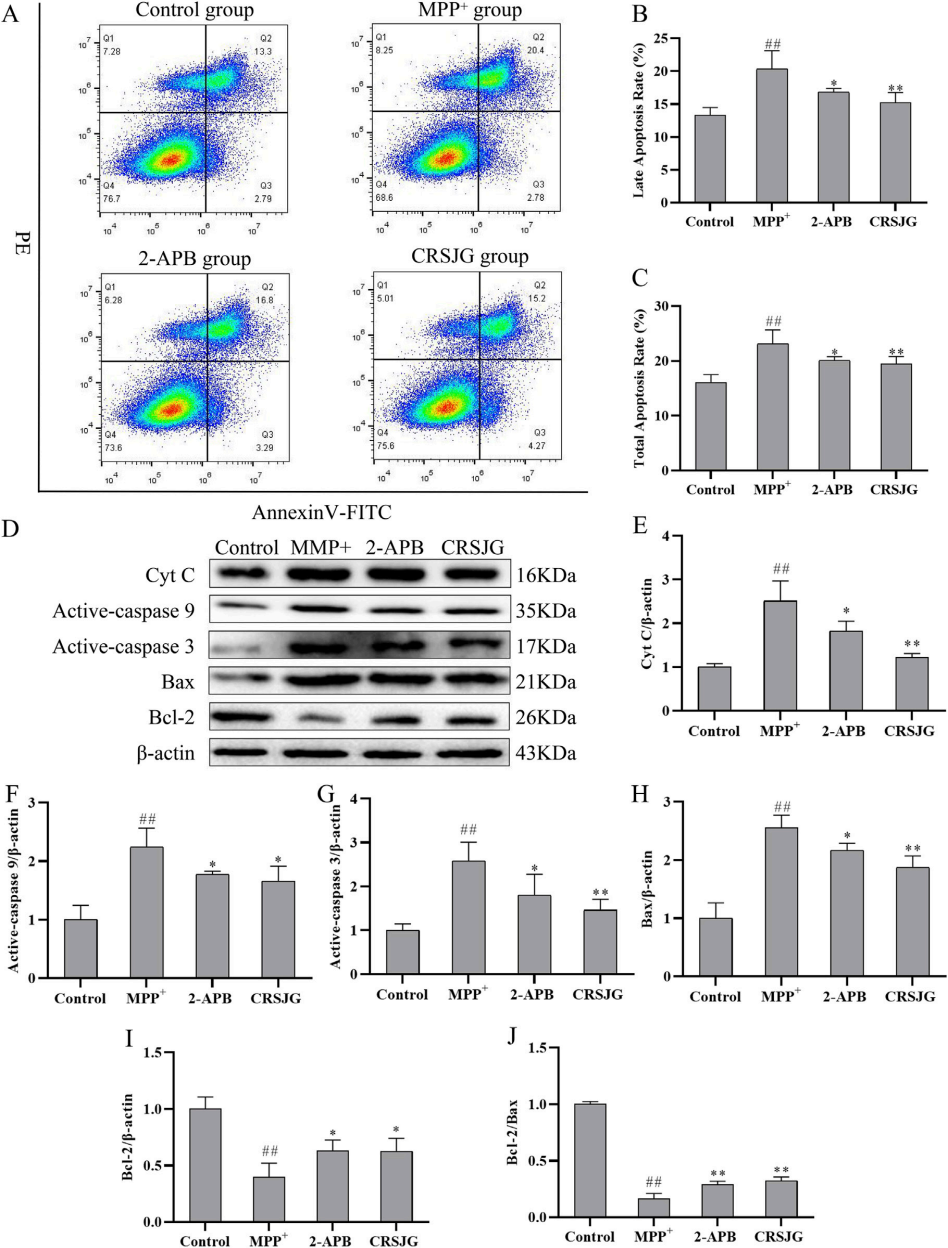
CRSJG can effectively protect neurons from apoptosis induced by MPP^+^. After treating with 1,000 μmol/L MPP^+^ -induced SH-SY5Y cells with 2.5% CRSJG-medicated serum or 100 μmol/L 2-APB for 24 h, **(A)** Flow cytometry was used to detect nerve cell apoptosis; **(B)** Late apoptosis rate; **(C)** Total cell apoptosis rate; **(D)** Western blot was used to detect the expression levels of apoptosis-related proteins; **(E)** Cyt C; **(F)** Active-caspase 9; **(G)** Active-caspase 3; **(H)** Bax; **(I)** Bcl-2; **(J)** Bcl-2/Bax. Results are showed as mean ± standard deviation (SD), of which **(A–C)** results are from four independent experiments, and **(D–J)** results are from three independent experiments. M: ##*P* < 0.01 vs. control group; * *P* < 0.05, ** *P* < 0.01 vs. MPP^+^ group.

The rates of late-apoptosis and total-apoptosis in MPP^+^ induced nerve cells were significantly elevated, with interventions using CRSJG and 2-APB effectively mitigating this MPP^+^ induced apoptosis (Figures 6A–C). Additionally, Western blot analyses indicated that CRSJG and 2-APB substantially decreased the MPP^+^ induced upregulation of apoptosis-related proteins (Figures 6D–J). These findings demonstrate that CRSJG possesses a protective effect against MPP^+^ induced neuronal apoptosis.

### CRSJG protect the structure and function of MAMs in MPP^+^-induced nerve cell damage

**FIGURE 7:**
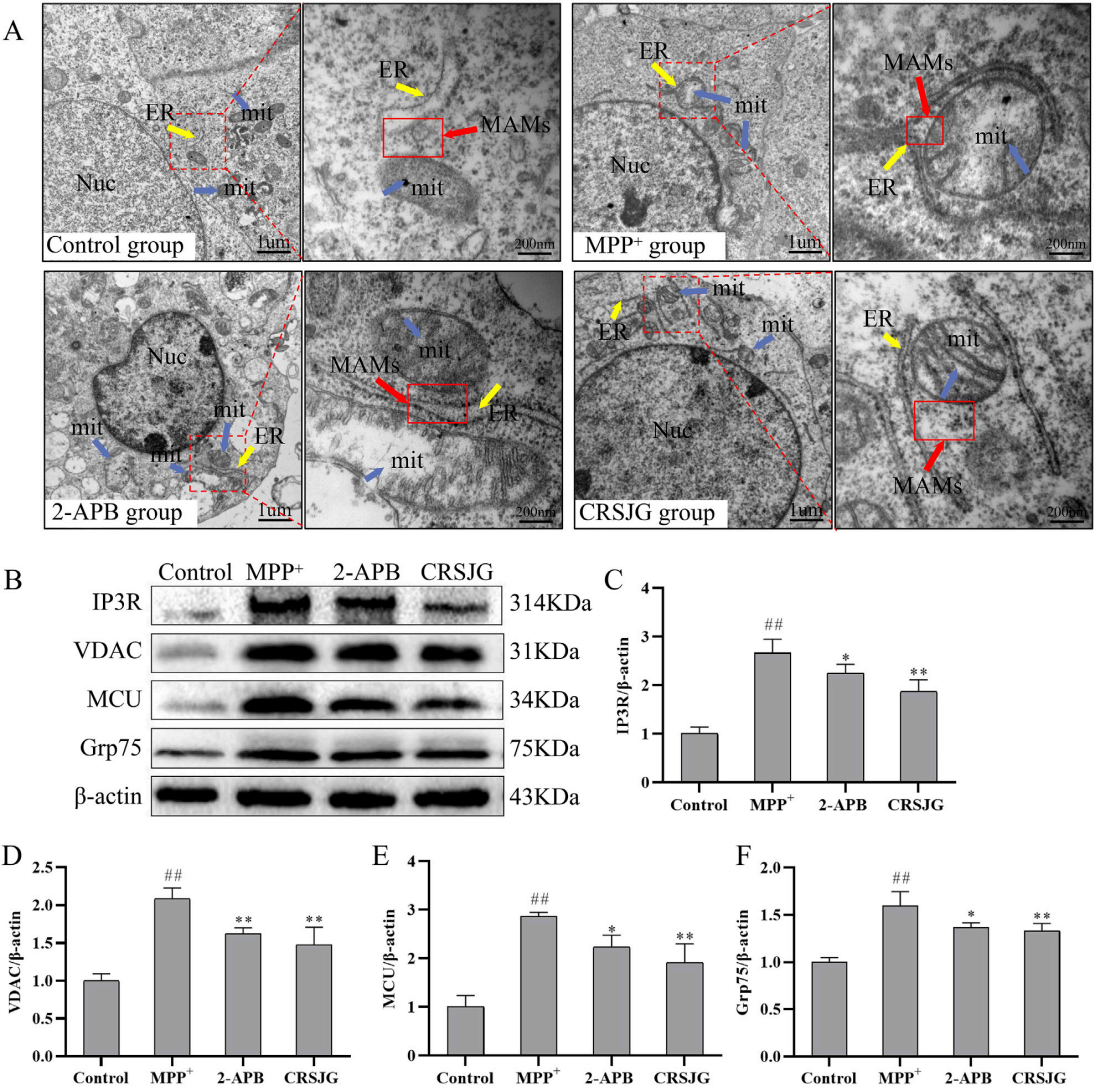
CRSJG protects the structure and function of MAMs in MPP^+^ induced nerve cells. After treating with 1,000 μmol/L MPP^+^ -induced SH-SY5Y cells with 2.5% CRSJG-medicated serum or 100 μmol/L 2-APB for 24 h, **(A)** The microscopic structure of MAMs was observed by transmission electron microscope, magnification (×20,000), magnification of red rectangle box (×,100,000); **(B)** Western blot was used to detect the expression of major proteins in the Ca^2+^ transport complex; **(C)** IP3R; **(D)** VDAC; **(E)** MCU; **(F)** Grp75. Results are from three independent experiments. M: # *P* < 0.05, ##*P* < 0.01 vs. control group; * *P* < 0.05, ** *P* < 0.01 vs. MPP^+^ group.

Utilizing transmission electron microscopy, we examined the microstructure of nerve cells to observe alterations in MAMs. MPP^+^ induced substantial damage to MAMs structure within nerve cells, characterized predominantly by mitochondrial swelling, fragmentation or complete loss of mitochondrial cristae, and densely surrounded mitochondria by the endoplasmic reticulum, leading to tighter connections within MAMs. Intervention with CRSJG and 2-APB partially restored the mitochondrial and endoplasmic reticulum structures, and significantly expanded the MAMs (Figure 7A).

To further elucidate the functional changes in MAMs, Western blot analysis was employed to assess the expression of key proteins within the Ca^2+^ transport complex. Expressions of IP_3_R, VDAC, MCU, and Grp75 were notably elevated in the MPP^+^ group, whereas interventions with CRSJG and 2-APB reduced these protein levels (Figures 7B–F). These findings suggest that CRSJG effectively ameliorates both the structure and function of MAMs in MPP^+^ induced nerve cell damage.

## Discussion

PD is among the most rapidly expanding neurological disorders, characterized by high prevalence and poor prognosis, thereby placing considerable burdens on families and society (Ben-Shlomo et al., 2024). Consequently, the discovery of effective treatments for PD remains essential. In this context, traditional Chinese medicine (TCM) has shown significant potential (Rabiei et al., 2019). Our research demonstrates that CRSJG-medicated serum effectively reduces nerve cell damage caused by MPP^+^, repairs MAMs, alleviates Ca^2+^ overload, and provides neuroprotection.

The progression of PD is characterized by a gradual disruption in Ca^2+^ homeostasis, notably mito-Ca^2+^ overload, which contributes to the death of nigral dopaminergic neurons (Rendón-Ochoa et al., 2022; Xu et al., 2022). MPP^+^, a neurotoxic metabolite of MPTP produced by monoamine oxidase B in glial cells, infiltrates DA neurons via the dopamine transporter. Once inside, MPP^+^ inhibits mitochondrial respiratory complex I, leading to neuronal damage (Chia et al., 2020). In experimental contexts, employing MPTP in animal models or MPP^+^ in cell models induces mitochondrial dysfunction, motor symptoms, dopaminergic neuron loss, and other hallmark features of PD. These models are crucial for studying mitochondria-related intracellular calcium signaling (Dovonou et al., 2023).

The study of TCM in cellular models faces several challenges. To more accurately simulate the *in vivo* metabolic state of drugs, we employed a serum pharmacology approach by using drug-medicated serum from CRSJG-fed animals rather than applying CRSJG directly to cells. Many components of CRSJG are complex herbal molecules that undergo extensive biotransformation-including digestion, absorption, and hepatic metabolism-before becoming pharmacologically active (Ma et al., 2017). By using serum from treated animals, we introduced the bioavailable, metabolized forms of CRSJG into the cell culture system, thereby achieving closer physiological relevance compared to the direct application of crude extracts. UPLC-MS/MS analysis of CRSJG-medicated serum confirmed the presence of various bioactive components derived from TCM. Specifically, echinacoside and acteoside are the main active constituents of *Cistanche deserticola*; paeoniflorin is the principal component of *Paeonia lactiflora*; and salvianolic acid B and tanshinone IIa are the key active compounds of *Salvia miltiorrhiza*. These constituents exhibit regulatory effects on oxidative stress, mitochondrial dysfunction, and Ca^2+^ homeostasis in PD (Wu et al., 2019; Subedi and Gaire, 2021; Li et al., 2022; Gong et al., 2024; Han et al., 2024).

The active components in CRSJG-medicated serum play a crucial role in regulating calcium signaling. Echinacoside has a high content in CRSJG-medicated serum, and previous studies have also shown that echinacoside has excellent PD efficacy. Experimental studies suggest that echinacoside exerts multiple neuroprotective effects, including mitochondrial protection, antioxidant and anti-inflammatory activity, suppression of endoplasmic reticulum stress, and autophagy regulation (Li et al., 2022). Several studies have also confirmed its regulatory effects on intracellular calcium levels (Lu et al., 2016; Haddish and Yun, 2023; Zhao et al., 2024). Paeoniflorin has similarly been shown to modulate intracellular Ca^2+^ and ROS levels, offering therapeutic potential for various neurodegenerative diseases (Zhu et al., 2018; Peng et al., 2022). Other major components of CRSJG-salvianolic acid B, acteoside, and tanshinone IIa-also contribute to the regulation of intracellular Ca^2+^ homeostasis (Song et al., 2015; Chen et al., 2017; Zhang et al., 2018). Our study observed significantly lower AUC values for acteoside and tanshinone IIa, which may be attributed to their chemical structures, physicochemical properties, and factors influencing drug absorption and distribution.

Our study revealed that the MFI of mito-Ca^2+^ in nerve cells induced by MPP^+^ was significantly elevated, indicating mito-Ca^2+^ overload. Concurrently, an increase in cyto-Ca^2+^ concentration was observed. Ca^2+^ is crucial in regulating mitochondrial function, and sustained elevation of cyto-Ca^2+^ may lead to mito-Ca^2+^ overload, ultimately resulting in mitochondrial dysfunction (Xu et al., 2022). Elevated mito-Ca^2+^ concentrations induce MMP breakdown and ROS release into the cytoplasm, serving as principal sensitizing signals within the intrinsic apoptotic pathway (Fan and Simmen, 2019). This cascade of stress responses, consistent with findings from other studies, was evident in our experiments (Wang et al., 2021; Park et al., 2022). Notably, alleviation of Ca^2+^ overload, paralleling the effect seen with the positive control group 2-APB, a classic antagonist of inositol 1,4,5-trisphosphate that inhibits endoplasmic reticulum Ca^2+^ from entering the cytoplasm, was paramount (Hsu et al., 2021). Furthermore, CRSJG treatment effectively restored the decreased MMP caused by mito-Ca^2+^ overload, inhibited ROS activation, and significantly reduced the apoptosis rate in nerve cells. These findings suggest that CRSJG-medicated serum may ameliorate Ca^2+^ overload induced by MPP^+^ and protect nerve cells from MPP^+^ induced damage.

Given that MAMs function as dynamic platforms for regulating cyto-Ca^2+^ and mito-Ca^2+^ (Marchi et al., 2018), our study focused on elucidating the impact of CRSJG-medicated serum on the structure and function of MAMs. We employed transmission electron microscopy to investigate the microstructure of the MAMs region in nerve cells. In nerve cells induced by MPP^+^, mitochondria displayed swelling, disruption of mitochondrial cristae, or even their disappearance. Additionally, the distance between the endoplasmic reticulum and mitochondrial membrane in the MAMs region was reduced, tightly encircling the mitochondria. These structural changes in the MAMs region suggest compromised cellular function, reduced Ca^2+^ buffering capacity, increased Ca^2+^ exchange between the endoplasmic reticulum and mitochondria, and a heightened risk of calcium overload (Hedskog et al., 2013; Wilson and Metzakopian, 2021). Concurrently, protein analysis showed a marked increase in the expression of the IP_3_R-VDAC-MCU complex, a key protein assembly in Ca^2+^ transport within MAMs, critical for heightened intracellular Ca^2+^ transport (Boulay et al., 1999; Dia et al., 2020; Allen and Tessem, 2022). The IP_3_R-VDAC-MCU complex actively transports cyto-Ca^2+^ into mitochondria, contributing to mito-Ca^2+^ overload and functional impairment (Dia et al., 2020). Treatment with CRSJG-medicated serum effectively corrected the abnormal morphology of mitochondria, endoplasmic reticulum, and MAMs, distinctly separated MAMs, and decreased the expression of the IP_3_R-VDAC-MCU Ca^2+^ transporter, indicating that CRSJG-medicated serum provides a protective effect against structural and functional damage of MAMs induced by MPP^+^.

**FIGURE 8:**
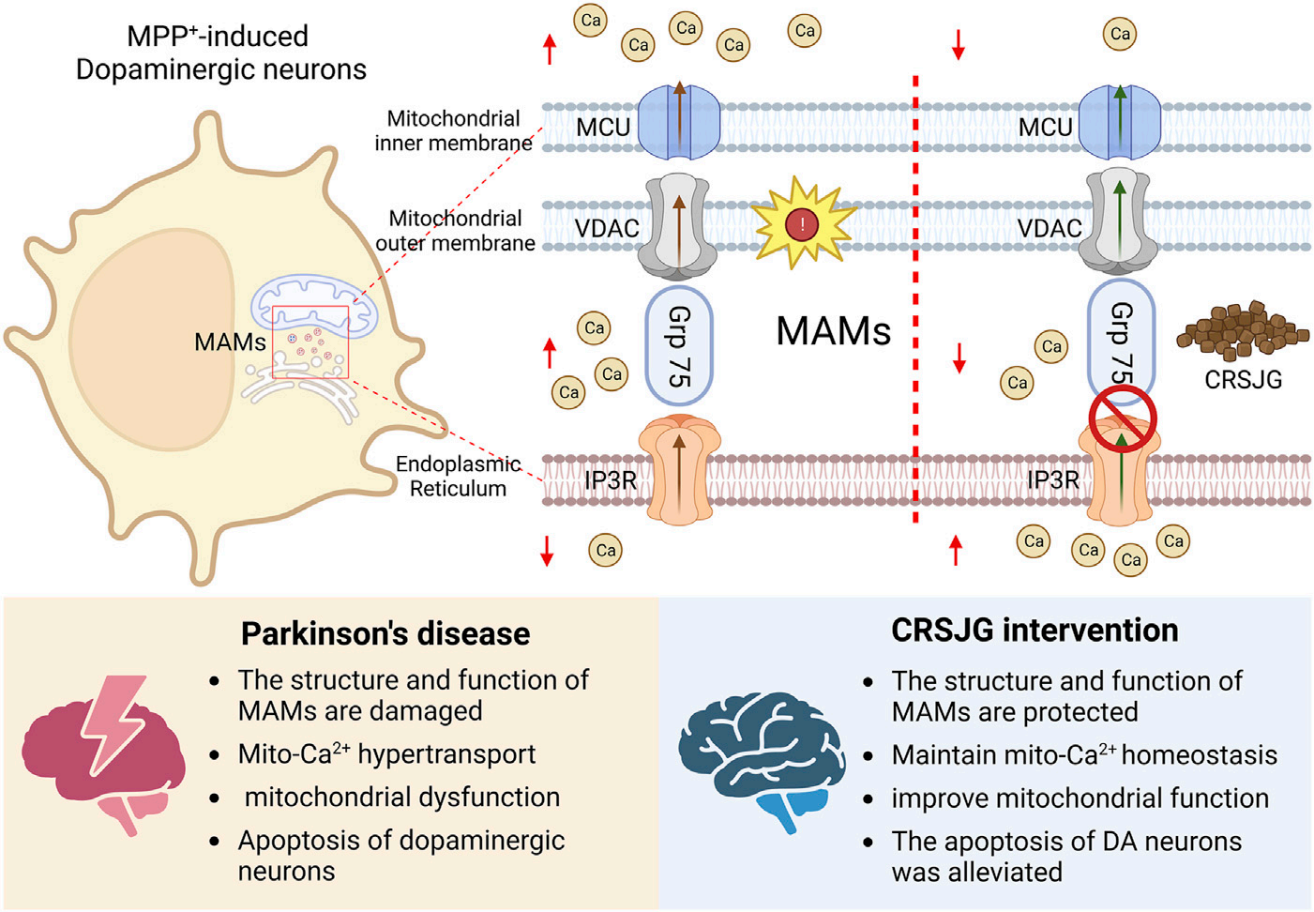
Neuroprotective effect of CRSJG-medicated serum. CRSJG-medicated serum can alleviate MPP^+^ induced neuronal apoptosis and improve the intracellular-Ca^2+^ disturbance induced by MPP^+^. The protective effect of CRSJG on nerve cells may be related to the improvement of the structure and function of MAMs.

Drug therapy targeting Ca^2+^ transporters in MAMs is a focal point in PD treatment research (Dey et al., 2020; Rajendran et al., 2022). CRSJG was developed by our team as part of *the Creation of Major New Drugs initiative within Major National Science and Technology Projects* (Grant No. 2019ZX09301154), specifically targeting clinical PD symptoms such as tremor and myotonia. Our *in vitro* studies on the traditional Chinese medicine CRSJG confirm that it effectively protects DA neurons in PD animal models, improves motor balance, and maintains mitochondrial integrity (Xu et al., 2020). Additionally, our findings indicate that CRSJG restores the structure and function of MAMs and mitigates Ca^2+^ overload, thereby contributing to its potential therapeutic efficacy against PD. These findings are detailed in Figure 8.

The precise mechanisms by which the various compounds in CRSJG protect MAMs and maintain intracellular Ca^2+^ balance remain unclear. Meanwhile, our previous studies have shown that even blank serum still has certain inevitable effects on cells. Future research should focus on validating the findings of this study in PD animal models and elucidating the actions of CRSJG’s major compounds. Notably, previous studies have demonstrated that CRSJG can alleviate endoplasmic reticulum stress and enhance dopaminergic neuron survival in PD rat models, suggesting potential pathways for its neuroprotective effects.

## Conclusion

Our study confirmed that CRSJG-medicated serum can effectively protect MPP^+^-induced PD cell models, enhance the structure and function of MAMs, and maintain intracellular Ca^2+^ homeostasis. These findings provide a theoretical and experimental basis for the clinical application of CRSJG in treating PD.

## Data Availability

The original contributions presented in the study are included in the article/Supplementary Material, further inquiries can be directed to the corresponding author.
