# Supplementary material for: Congrong Shujing Granules ameliorates mitochondrial associated membranes to against MPP+-induced neurological damage in the cellular model of Parkinson’s disease
Source: Front Pharmacol. 2025 May 30;16:1509317. doi: 10.3389/fphar.2025.1509317 (PMC12162334; doi:10.3389/fphar.2025.1509317)
Supplement: Supplementary file 1 [file DataSheet2.pdf]

Western blot was used to detect the expression levels of apoptosis-related proteins

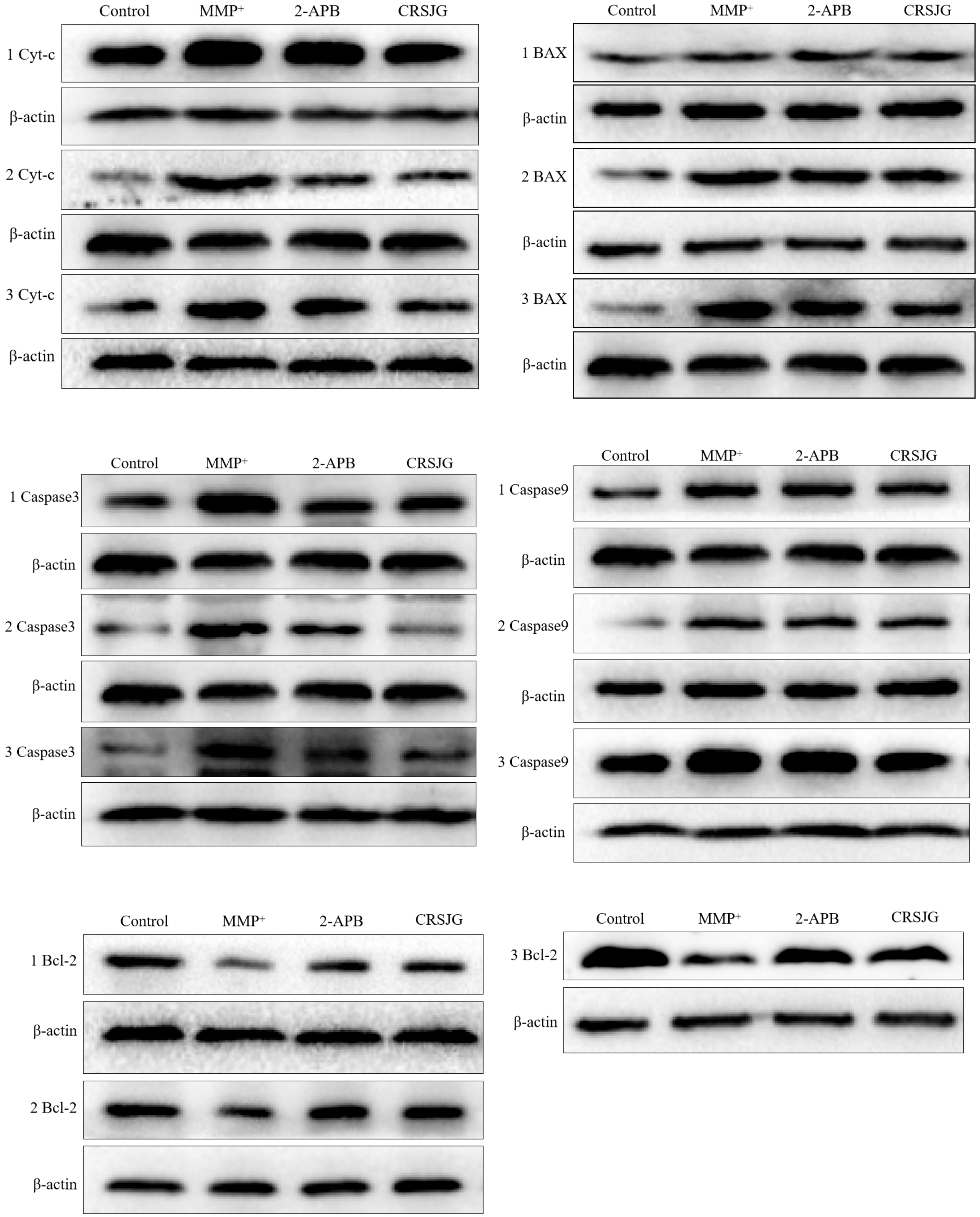

	Target protein（Gray value）			β-actin（Gray value）			ratio		
	sample 1	sample 2	sample 3	sample 1	sample 2	sample 3	sample 1	sample 2	sample 3
Cyt c	4092725	2756936	2541462	7538710	5150400	4867065	0.542894607	0.535285803	0.493449441
	8587600	5862624	6866607	8292063	4349960	5343520	1.03564095	1.347742048	1.57854486
	8015000	3948080	5069592	7623168	4804454	5072315	1.051400153	0.821754147	0.999463164
	5097680	3257415	2971980	7852935	4953528	5005455	0.649143282	0.657594951	0.599972383
Bax	1752978	3802638	1988040	2730566	6023710	5150400	0.641983384	0.631278398	0.385997204
	4104240	10199610	6124720	3157308	6646252	4349960	1.299917525	1.534640877	1.407994556
	3724920	8296214	5401225	3081312	6589336	4804454	1.208874661	1.259036419	1.124212033
	3064950	7201643	5543718	3361824	6687695	4953528	0.911692581	1.076849797	1.119145385
Bcl-2	6500772	7090710	7867600	4867065	6501088	6023710	1.335665745	1.090695896	1.306105374
	2442325	4749657	2416655	5343520	7172235	6646252	0.457062947	0.662228301	0.363611702
	4095503	6187512	4304765	5012315	7019516	6589336	0.817088112	0.88147274	0.653292684
	3964213	6680296	4221566	5005455	7314109	6687695	0.791978551	0.913343785	0.631243799
Active-caspase 3	3045910	3798240	3502680	5150400	5150400	6149880	0.591392902	0.737465051	0.569552577
	6725950	8417556	9947664	4349960	4349960	6940720	1.54620962	1.935088139	1.433232287
	4180295	5226372	8731200	4804454	4804454	5962732	0.870087423	1.087818095	1.464295226
	3713045	4917564	7434984	4953528	4953528	7198503	0.749575858	0.99273972	1.032851414
Active-caspase 9	5467506	1901133	2356868	5150400	2730566	2071994	1.061569199	0.696241365	1.13748785
	9100134	5984388	5232294	4349960	3157308	2090370	2.092004064	1.895408367	2.503046829
	8491577	5261984	3802491	4804454	3081312	2272816	1.767438506	1.707708924	1.673030725
	7634836	4648611	3833600	4953528	3361824	2046646	1.54129259	1.382764535	1.873113377

Western blot was used to detect the expression of major proteins in the Ca2+ transport complex

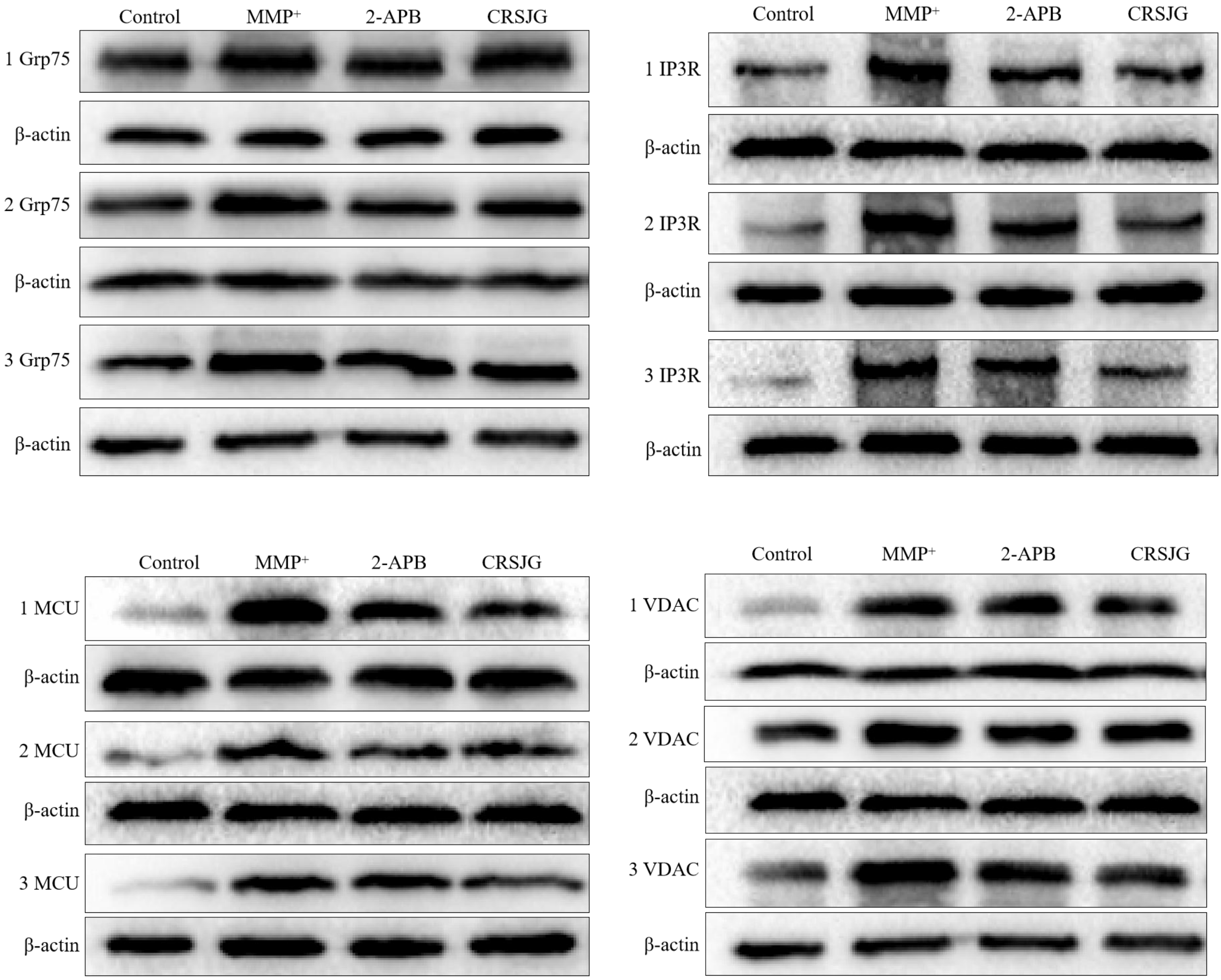

	Target protein（Gray value）			β-actin（Gray value）			ratio		
	sample 1	sample 2	sample 3	sample 1	sample 2	sample 3	sample 1	sample 2	sample 3
IP3R	3303020	1801780	1144550	4867065	2730566	1482532	0.678647193	0.659855869	0.772023808
	12222720	4681596	3073710	5343520	3157308	1584266	2.287391083	1.482780901	1.940147677
	7900928	4217670	2799300	5012315	3081312	1512930	1.576303165	1.368790308	1.850250838
	6794928	3268980	2551050	5005455	3361824	1483552	1.357504563	0.972382849	1.719555499
VDAC	3677990	4244157	5452800	6501088	4867065	6023710	0.565749918	0.872015681	0.905222861
	9692440	8253376	12956346	7172235	5343520	6646252	1.351383495	1.544557894	1.949421418
	7463932	6596512	9079190	7019516	5012315	6589336	1.063311488	1.316060942	1.377861138
	7115622	6677024	7212896	7314109	5005455	6687695	0.97286245	1.333949461	1.078532439
MCU	3133140	1815345	1354848	5150400	4867065	2730566	0.60832945	0.372985567	0.496178448
	6261760	5246774	6254115	4349960	5343520	3157308	1.439498294	0.981894706	1.98083779
	5198668	3558485	5254831	4804454	5012315	3081312	1.082051779	0.709948397	1.70538751
	3933300	3254170	5234500	4953528	5005455	3361824	0.794040127	0.650124714	1.557041654
GRP75	6648480	6381052	5784603	6501088	7538710	6023710	1.022671897	0.846438184	0.960305692
	10130452	11635144	11312828	7172235	8292063	6646252	1.412453998	1.403166377	1.702136482
	9003715	9522903	8765337	7019516	7623168	6589336	1.282668919	1.249205448	1.330230694
	9478560	9763304	8120220	7314109	7852935	6687695	1.295928185	1.243268154	1.2142031
