# Supplementary material for: Congrong Shujing Granules ameliorates mitochondrial associated membranes to against MPP+-induced neurological damage in the cellular model of Parkinson’s disease
Source: Front Pharmacol. 2025 May 30;16:1509317. doi: 10.3389/fphar.2025.1509317 (PMC12162334; doi:10.3389/fphar.2025.1509317)
Supplement: Supplementary file 8 [file DataSheet3.pdf]

Gating strategy of mito/cyto-Ca^{2+} analysis

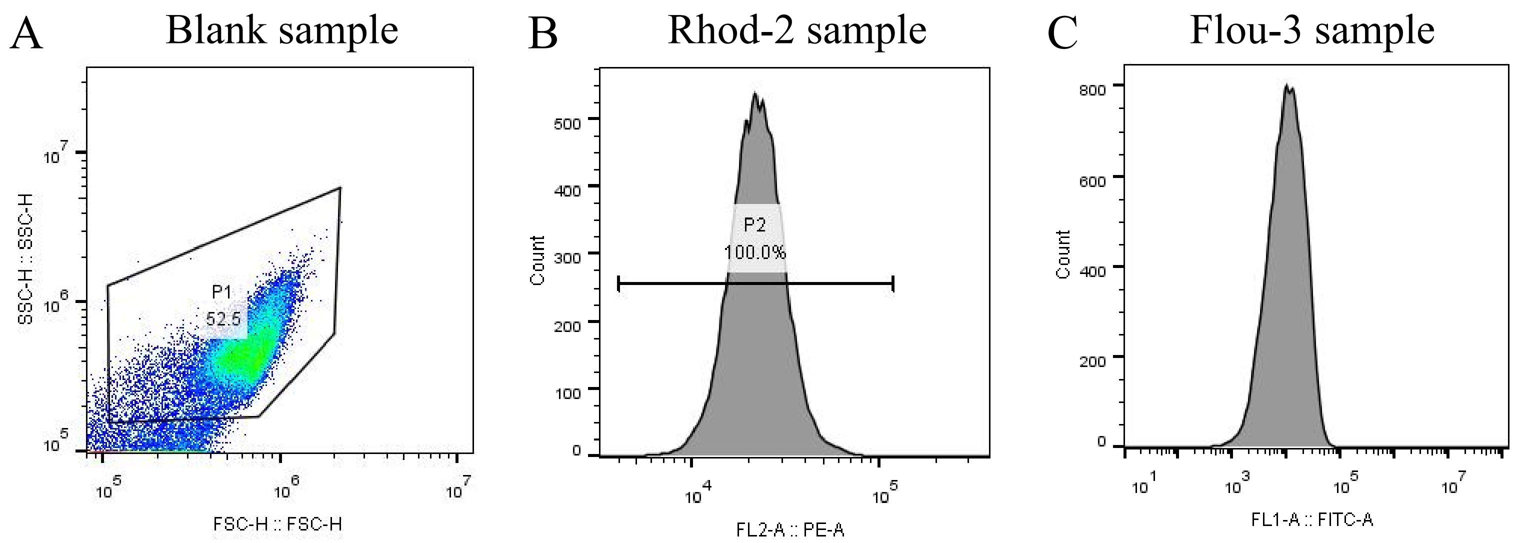

In the analysis of intracellular Ca^{2+} stained by Rhod-2 and Flou-3, we adopted the following gating strategy:

A: In the blank sample, we used SSA-H and FSC-H to select the aggregated cells.

B: In the Rhod-2 stained sample, we used the PE-A histogram to determine the fluorescence intensity of Rhod-2.

C: In Flou-3 stained samples, we used the FITC-A histogram to determine the fluorescence value intensity of Flou-3.

In the experiment, there were significant differences in Ca^{2+} staining of the samples in each group. We used the MFI of PE-A for late statistical analysis of mito-Ca^{2+} levels and the MFI of FITC-A for late statistical analysis of cyto-Ca^{2+} levels.

Gating strategy of MMP analysis

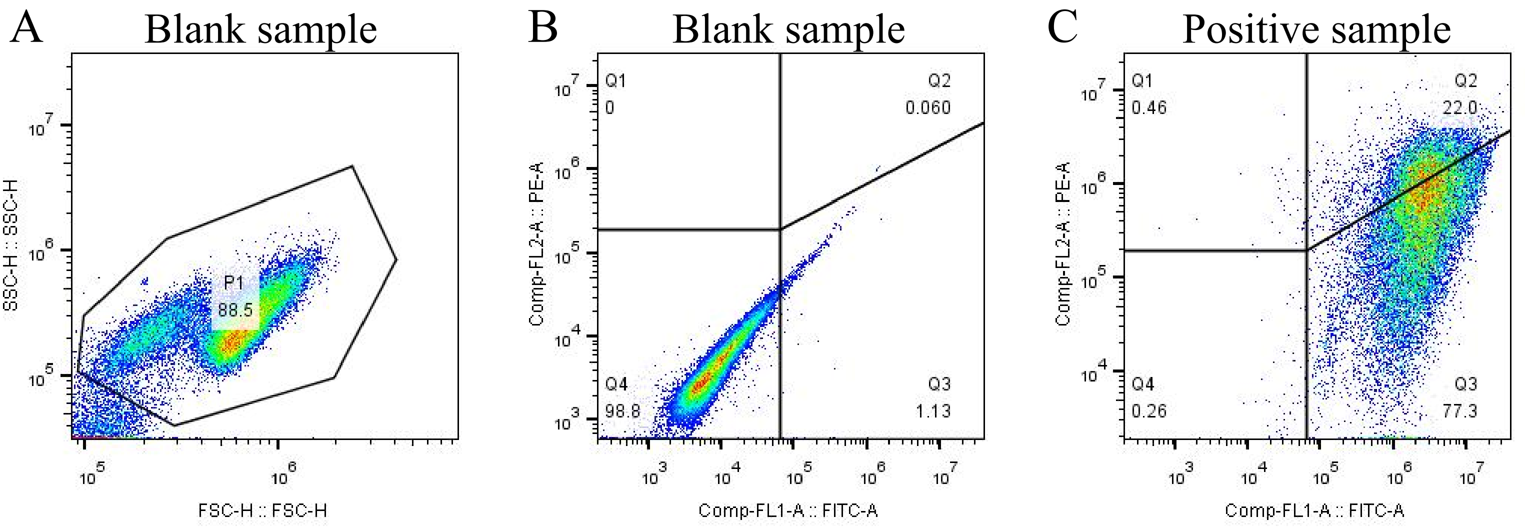

In the analysis of intracellular MMP levels, we adopted the following gating strategy:

A: In the blank sample, we used SSA-H and FSC-H to select the aggregated cells.

B: In the blank sample, we placed all the negative cells in the Q4 region of the blank sample.

C: In the positive sample, our principle is to classify the positive cells as far as possible into the Q3 region. However, since after JC-1 staining, there was a clear grouping of high-MMP cells and low-MMP cells in each group, we adjusted the division of Q2 and Q3 regions according to the actual situation, and divided them at the midpoint of the grouping.

Our gating strategy was carried out in accordance with the ***enhanced mitochondrial membrane potential assay kit with JC-1*** specification, and in consultation with Beyotime's flow analysis technical consultants. In the data analysis, we calculated the proportion of Q2 and Q3 regions , and used Q2/Q3 as the standard to evaluate the MMP levels.

Gating strategy of apoptosis analysis

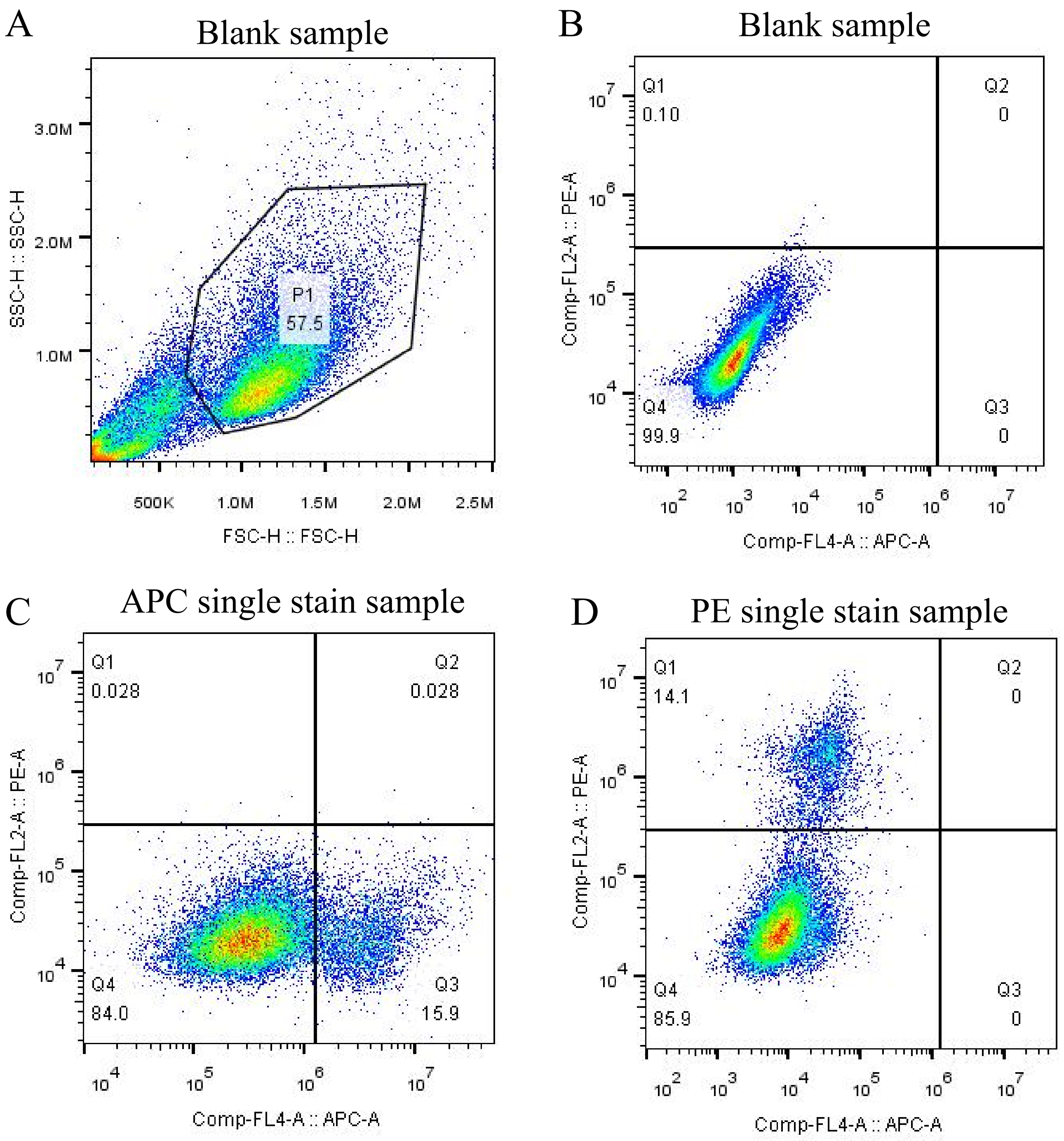

In the analysis of apoptosis cells, we adopted the following gating strategy:

A: In the blank sample, we used SSA-H and FSC-H to select the aggregated cells.

B: In the blank sample, we placed all the negative cells in the Q4 region of the blank sample.

C: In the ANNEXIN-APC monochromic samples, we used ANNEXIN-APC to divide the cells into two distinct clusters that are clearly spaced apart, and we distinguished the APC^- and APC^+ cells at the midpoint of the cluster.

D: In the PI-PE monochromatic sample, we use PI-PE to divide the cells into two distinct groups that are clearly separated from each other, and we distinguish PE^- and PE^+ cells at the midpoint of the group.

Our gating strategy was carried out in accordance with the *ABBkine ANNEXIN V-647 APOPTOSIS Detection KIT* specification, and in consultation with Beyotime's flow analysis technical consultants. In the experiment, we adopted a control strategy for fluorescence Minus One (FMO), which can effectively help us distinguish between negative cells and positive signals from two different dyes. In the results of the experiment, the test cells were clearly divided into three distinct groups, APC^-/PE^- cell group (Q4 region), APC^+/PE^- cell group (Q3 region), and APC^+/PE^+ cell group (Q2 region). In the data analysis, we calculated the proportion of Q2 and Q3 regions.
