# Supplementary material for: Congrong Shujing Granules ameliorates mitochondrial associated membranes to against MPP+-induced neurological damage in the cellular model of Parkinson’s disease
Source: Front Pharmacol. 2025 May 30;16:1509317. doi: 10.3389/fphar.2025.1509317 (PMC12162334; doi:10.3389/fphar.2025.1509317)
Supplement: Supplementary file 11 [file DataSheet1.pdf]

Figure 1 related data

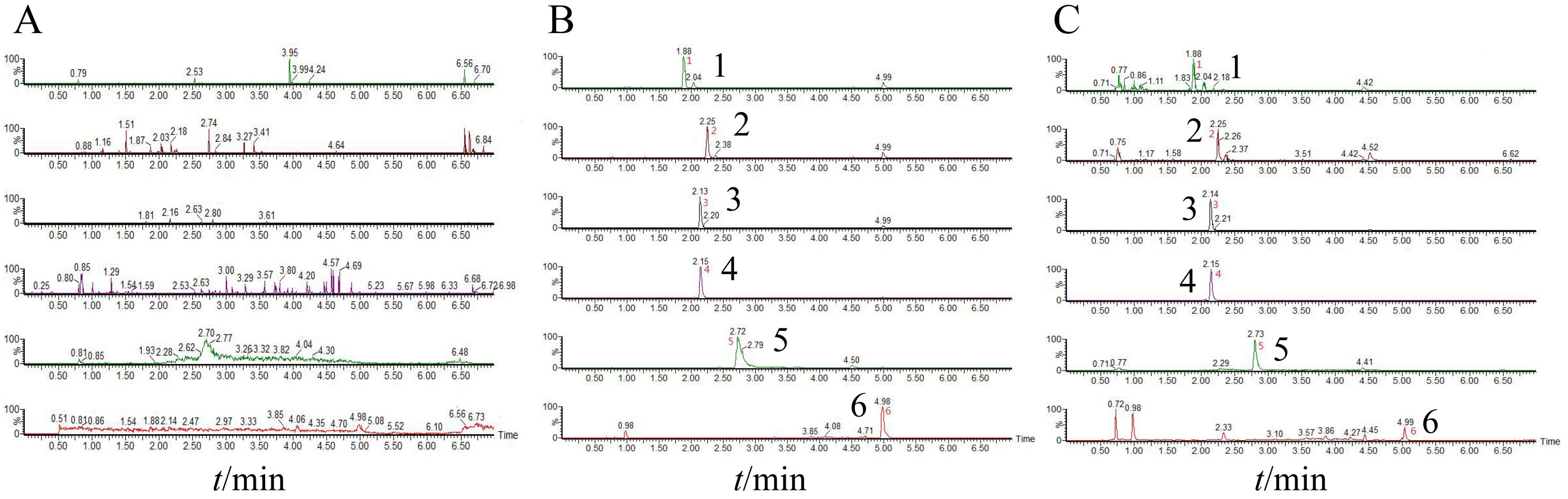

D

The results of recovery assay, extraction recovery assay and matrix effects

Component	Theory concentration / ng·mL^{-1}	recovery assay（n=5） Average/%	recovery assay（n=5） RSD/%	extraction recovery assay（n=5） Average/%	extraction recovery assay（n=5） RSD/%	matrix effects（n=5） Average/%	matrix effects（n=5） RSD/%
Echinacoside	422.730	99.695	3.114	93.705	7.670	91.20	2.93
	4227.300	95.977	2.881	90.503	3.887	91.49	6.15
	42273.000	99.372	2.598	91.061	4.166	92.07	4.89
Paeoniflorin	8.858	90.321	10.903	95.749	6.007	99.39	4.49
	88.575	108.130	2.191	89.668	2.629	96.88	1.81
	885.750	105.326	4.380	97.150	6.370	97.44	4.41
Salvianolic acid B	8.542	93.320	1.252	96.431	6.477	96.43	7.13
	85.417	95.277	4.295	97.822	4.549	100.6	2.39
	854.167	105.179	1.477	94.812	4.813	99.79	6.69
Acteoside	2.012	95.963	3.380	91.091	5.606	94.40	5.58
	20.115	97.389	6.376	87.237	2.940	97.39	2.93
	201.150	98.118	3.979	90.580	6.830	98.24	2.38
Tanshinone IIA	0.457	113.253	2.267	87.760	4.514	93.37	7.71
	4.570	106.659	1.905	90.733	6.846	99.51	10.33
	45.700	104.828	3.291	91.969	2.817	98.24	7.55

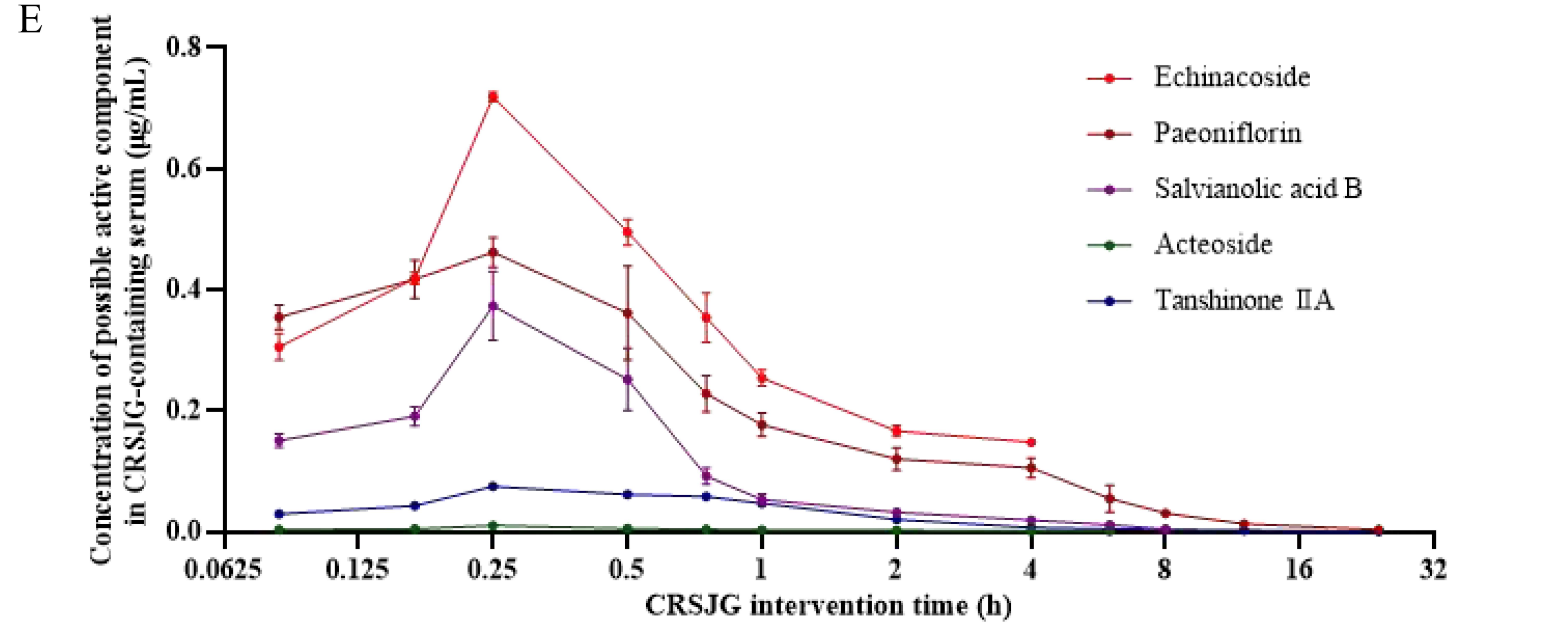

Figure 1. MRM analysis of the main components in CRSJG-containing serum.

(A-C) Specificity test of the main components of CRSJG-containing serum and rutin, (A) blank serum; (B) Mixed standard solution of six components in blank serum; (C) CRSJG-containing serum; 1. echinacoside; 2. acteoside; 3. internal standard (rutin); 4. paeoniflorin; 5. salvianolic acid B; 6. tanshinone II A. (D) The results of recovery assay, extraction recovery assay and matrix effects; (E) Pharmacokinetics of main components of CRSJG-containing serum.

The results of recovery assay, extraction recovery assay and matrix effects

Recovery assay（n=5）								
Compound	Theory concentration ($ng \times mL^{-1}$)	1	2	3	4	5	Average recovery (%)	RSD (%)
Echinacoside	422.7	97.78	98.57	97.62	105.1	99.40	99.69	3.11
	4227	97.99	91.32	98.02	96.77	95.79	95.98	2.88
	42273	99.13	102.7	95.49	99.64	99.87	99.37	2.60
Paeoniflorin	8.858	107.56	89.13	85.63	83.41	85.88	90.32	10.90
	88.58	106.1	107.4	112.2	108.1	106.8	108.1	2.19
	885.8	107.3	99.1	110.0	108.3	101.9	105.3	4.38
Salvianolic acid B	8.542	91.47	92.93	93.91	94.44	93.85	93.32	1.25
	85.42	92.36	91.20	101.63	96.40	94.79	95.28	4.29
	854.2	105.3	104.8	103.5	107.7	104.5	105.2	1.48
Acteoside	2.012	90.45	96.71	97.49	96.26	98.91	95.96	3.38
	20.12	95.35	93.26	108.1	96.95	93.24	97.39	6.38
	201.2	103.1	100.0	94.23	99.23	94.06	98.12	3.98
Tanshinone IIA	0.4570	115.5	113.3	116.0	111.6	109.9	113.3	2.27
	4.570	109.3	105.4	105.5	108.4	104.7	106.7	1.90
	45.70	106.9	103.5	103.1	101.0	109.7	104.8	3.29

Extraction recovery assay（n=5）								
Compound	Theory concentration ($ng \times mL^{-1}$)	1	2	3	4	5	Average recovery (%)	RSD (%)
Echinacoside	422.7	95.42	91.97	105.13	89.61	86.39	93.70	7.67
	4227	96.09	91.11	87.19	90.25	87.88	90.50	3.89
	42273	86.44	93.49	90.47	88.84	96.07	91.06	4.17
Paeoniflorin	8.858	96.14	93.13	87.56	102.35	99.57	95.75	6.01
	88.58	92.01	90.01	85.82	91.00	89.51	89.67	2.63
	885.8	90.29	103.0	90.62	100.2	101.7	97.15	6.37
Salvianolic acid B	8.542	93.84	92.50	104.46	89.84	101.52	96.43	6.48
	85.42	95.68	93.51	98.12	96.59	105.20	97.82	4.55
	854.2	99.58	94.38	99.30	91.20	89.60	94.81	4.81
Acteoside	2.012	90.29	83.22	90.70	96.12	95.12	91.09	5.61
	20.12	89.39	86.73	90.28	84.14	85.64	87.24	2.94
	201.2	97.47	96.37	88.08	88.15	82.83	90.58	6.83
Tanshinone IIA	0.4570	87.63	89.15	81.29	92.01	88.72	87.76	4.51
	4.570	86.97	100.24	86.75	93.87	85.84	90.73	6.85
	45.70	91.75	89.50	89.51	95.37	93.73	91.97	2.82

Matrix effect assay（n=5）								
Compound	Theory concentration ($ng \times mL^{-1}$)	1	2	3	4	5	Average recovery (%)	RSD (%)
Echinacoside	422.7	94.94	91.04	92.31	89.90	87.79	91.20	2.93
	4227	86.30	85.68	93.61	92.61	99.23	91.49	6.15
	42273	92.12	91.60	92.20	98.57	85.87	92.07	4.89
Paeoniflorin	8.858	105.8	93.78	97.85	101.3	98.24	99.39	4.49
	88.58	94.83	98.29	95.13	98.40	97.75	96.88	1.81
	885.8	98.97	89.81	99.31	98.9	100.2	97.44	4.41
Salvianolic acid B	8.542	93.62	89.34	91.74	104.9	102.5	96.43	7.13
	85.42	101.5	99.45	104.1	99.92	97.77	100.6	2.39
	854.2	101.7	109.2	90.73	97.97	99.29	99.79	6.69
Acteoside	2.012	89.56	103.3	92.97	94.31	91.84	94.40	5.58
	20.12	94.16	101.9	96.59	97.72	96.56	97.39	2.93
	201.2	95.69	97.23	97.71	98.58	102.0	98.24	2.38
Tanshinone IIA	0.4570	96.19	101.9	91.00	95.29	82.51	93.37	7.71
	4.570	102.3	95.63	101.2	113.27	85.10	99.51	10.33
	45.70	90.08	109.61	97.91	99.93	93.65	98.24	7.55

Stability test of CRSJG-containing serum

Serum stability test at indoor temperature（n=5）

Compound	Theory concentration	Relative error (%)					Mean (%)
		1	2	3	4	5	
Echinacoside	422.7	-4.10	-4.07	0.18	-5.59	1.01	-2.51
	4227	-2.60	-3.67	0.70	-1.87	3.55	-0.78
	42273	-0.59	-1.96	-1.34	3.48	4.18	0.76
Paeoniflorin	8.858	2.54	-3.90	-0.38	-1.08	-0.77	-0.72
	88.58	1.29	0.53	1.35	2.12	2.71	1.60
	885.8	1.28	0.37	0.74	0.16	-2.11	0.09
Salvianolic acid B	8.542	-0.88	-0.59	-6.12	-2.94	-7.33	-3.57
	85.42	-2.65	-4.62	3.12	-4.66	-7.83	-3.33
	854.2	2.38	2.76	-4.54	-2.66	-7.85	-1.98
Acteoside	2.012	-5.60	-8.68	-1.78	-3.34	-4.36	-4.75
	20.12	-1.82	-2.76	2.43	-0.80	6.30	0.67
	201.2	3.82	1.15	1.21	3.17	-7.21	0.43
Tanshinone IIA	0.4570	-3.83	0.66	-0.40	-6.62	-3.03	-2.65
	4.570	-6.99	-1.21	2.88	-7.80	2.53	-2.12
	45.70	-2.94	4.07	-7.50	-4.75	-1.35	-2.49

Serum freeze-thaw stability test（n=5）

Compound	Theory concentration	Relative error (%)					Mean (%)
		1	2	3	4	5	
Echinacoside	422.7	-4.32	-1.45	-5.11	-3.88	0.27	-2.90
	4227	-6.56	-4.29	-8.50	0.98	2.72	-3.13
	42273	0.95	7.02	-1.52	-5.86	-0.30	0.06
Paeoniflorin	8.858	-0.74	-3.93	1.61	1.43	-0.80	-0.49
	88.58	0.27	-7.37	1.45	-0.79	0.03	-1.28
	885.8	-1.20	-0.63	0.60	-0.20	-1.12	-0.51
Salvianolic acid B	8.542	-6.60	0.84	-4.99	-1.14	-8.29	-4.04
	85.42	-1.98	-4.52	1.69	2.91	-1.93	-0.77
	854.2	-7.75	-4.02	-0.91	0.84	-1.07	-2.58
Acteoside	2.012	4.67	-1.29	7.88	4.24	-3.89	2.32
	20.12	-4.09	-8.16	2.64	-2.66	-9.65	-4.38
	201.2	-1.27	3.63	2.35	0.88	-1.37	0.84
Tanshinone IIA	0.4570	3.19	-1.54	3.96	2.60	-10.05	-0.37
	4.570	-8.34	-7.88	0.22	-7.28	-6.43	-5.94
	45.70	3.11	-0.14	-4.52	-5.53	-3.82	-2.18

Long-term freezing stability test of serum（n=5）

Compound	Theory concentration	Relative error (%)					Mean (%)
		1	2	3	4	5	
Echinacoside	422.7	-0.14	-6.33	-6.36	-9.27	-4.25	-5.27
	4227	-8.32	-4.70	-5.74	-2.12	-0.33	-4.24
	42273	-1.22	2.47	-3.80	-2.33	1.28	-0.72
Paeoniflorin	8.858	1.60	-5.63	2.37	-5.99	-3.03	-2.14
	88.58	0.45	-0.52	-4.61	-2.56	3.22	-0.80
	885.8	1.27	-1.00	0.54	-1.29	-1.54	-0.41
Salvianolic acid B	8.542	-6.47	0.55	-1.35	0.76	-4.51	-2.20
	85.42	-0.64	2.47	-2.70	-4.28	3.37	-0.35
	854.2	2.90	-1.99	-7.40	0.41	-0.87	-1.39
Acteoside	2.012	2.76	-13.68	2.39	-4.75	-9.87	-4.63
	20.12	-11.32	-6.24	-0.95	-10.20	-1.60	-6.06
	201.2	-2.77	3.57	-8.55	-0.31	-8.67	-3.35
Tanshinone IIA	0.4570	2.57	-3.87	-2.73	1.36	-2.95	-1.12
	4.570	2.26	-9.31	0.67	-5.76	0.69	-2.29
	45.70	0.98	4.58	-13.84	0.53	-6.76	-2.90

Pharmacokinetic standard curve

Echinacoside		Paeoniflorin		Salvianolic acid B		Acteoside		Tanshinone IIA	
Concentration（ug/mL）	Peak area ratio	Concentration（ug/mL）	Peak area ratio	Concentration（ug/mL）	Peak area ratio	Concentration（ug/mL）	Peak area ratio	Concentration（ug/mL）	Peak area ratio
0.1409	0.001583	2.953	0.03540	2.847	0.003593	0.6705	0.000692258	0.1523	0.06606
0.4227	0.01602	8.858	0.09129	8.542	0.1774	2.012	0.036276866	0.4570	0.1945
2.114	0.08426	44.29	0.4428	42.71	1.242	10.06	0.251657437	2.285	0.7851
4.227	0.1929	88.58	1.048	85.42	2.766	20.12	0.529674208	4.570	1.5613
21.14	1.044	442.9	4.490	427.1	15.08	100.6	2.827688425	22.85	6.4265
42.27	2.035	885.8	9.844	854.2	31.56	201.2	5.543049782	45.70	14.7330
126.8	7.960	2657	25.41	2563	105.0	603.5	20.60860304	137.1	36.2086

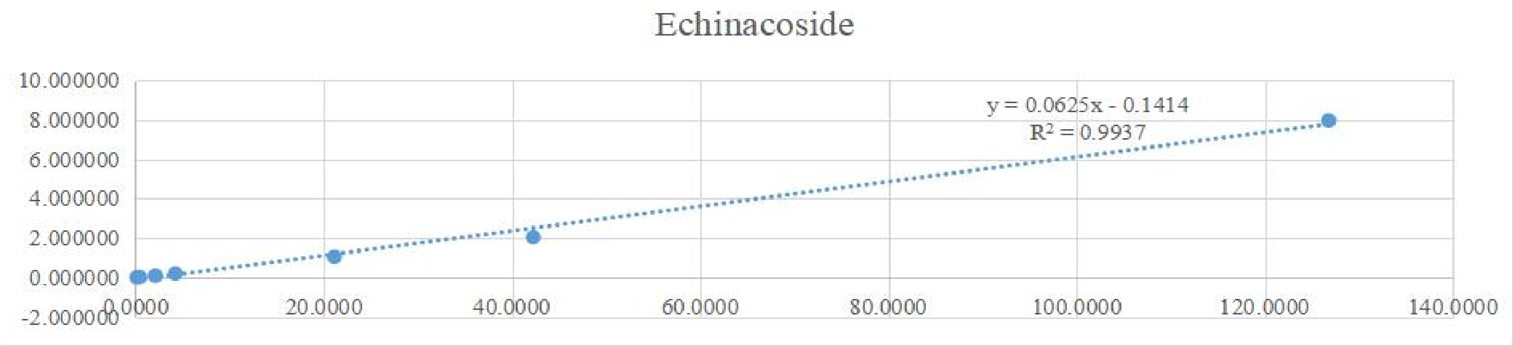

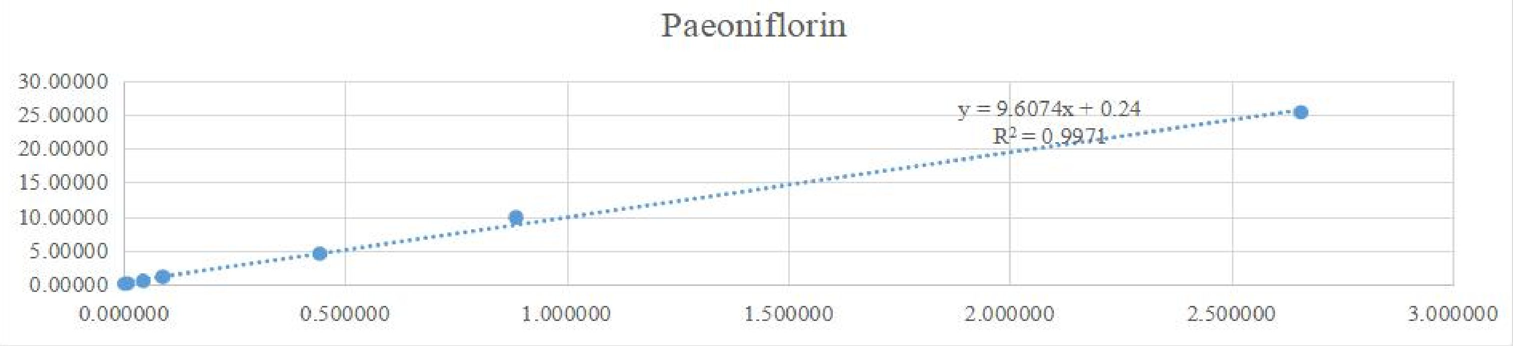

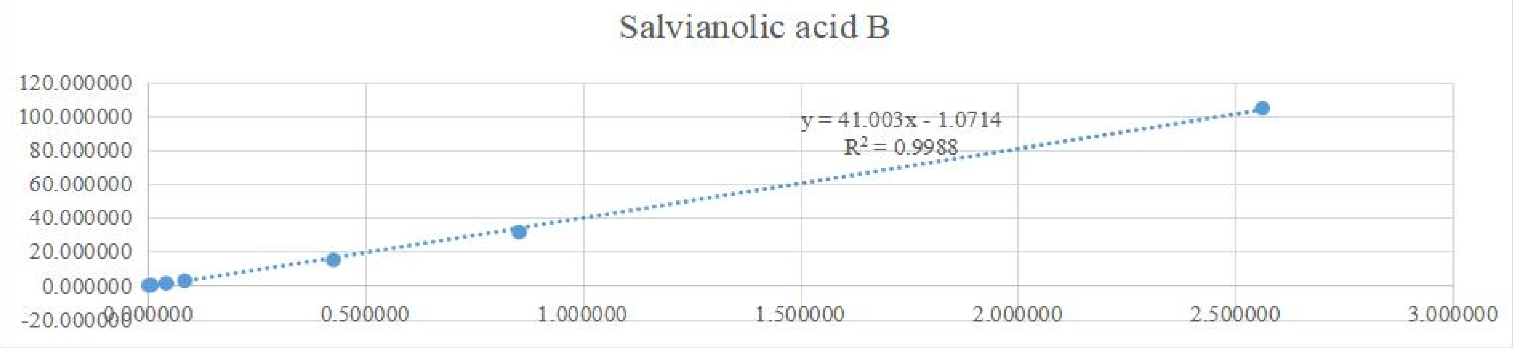

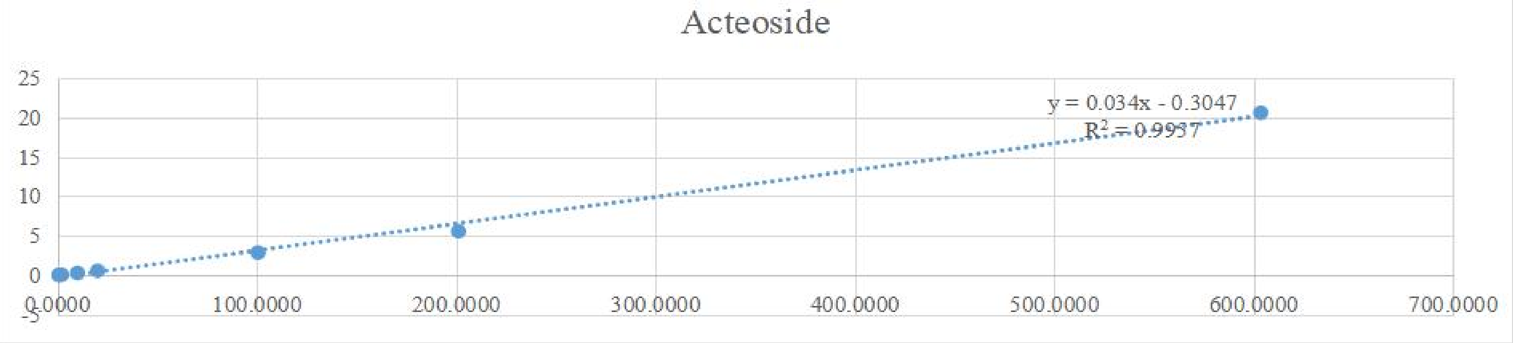

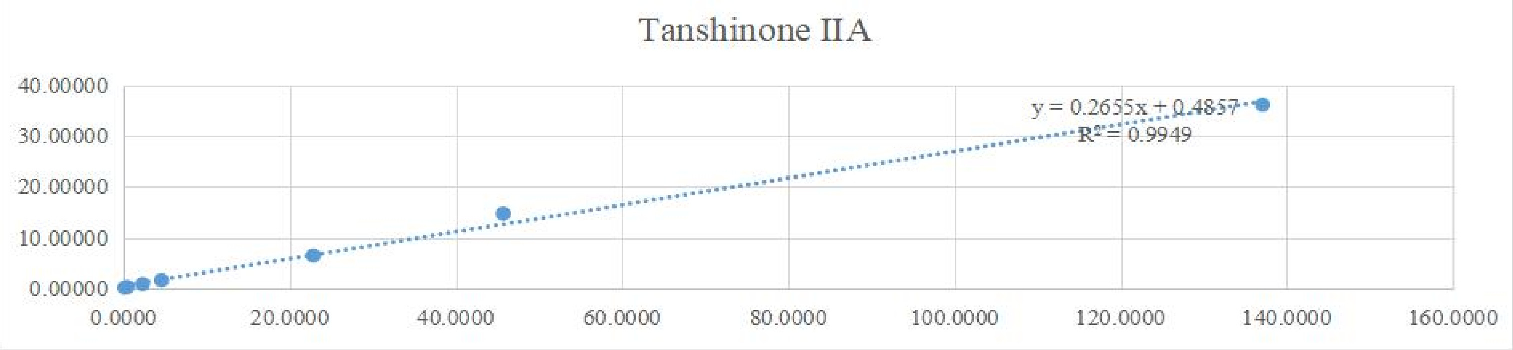

Pharmacokinetics of main components of CRSJG-containing serum

Concentrations of main compounds in CRSJG-containing serum（n=6）

Component	Time/h	Concentration（ng•mL^{-1}）						Mean /ng•mL^{-1}	RSD	SD
		1	2	3	4	5	6			
Echinacoside	0.083	294.2	332.3	326.7	273.7	310.9	296.7	305.8	7.18	21.95
	0.167	428.1	401.4	421.9	424.1	415.6	423.9	419.2	2.29	9.61
	0.25	716.5	717.4	717.8	705.9	731.0	723.7	718.7	1.16	8.33
	0.5	500.6	464.9	528.4	480.5	501.9	496.9	495.5	4.34	21.51
	0.75	397.9	312.5	355.4	297.1	380.0	381.3	354.0	11.52	40.79
	1	262.4	244.2	252.6	234.4	261.3	271.5	254.4	5.31	13.50
	2	175.4	157.9	152.4	171.2	171.1	171.9	166.7	5.53	9.22
	4	155.7	145.8	147.3	144.4	150.4	144.4	148.0	2.96	4.38
	6	ND	ND	ND	ND	ND	ND	—	—	—
Paeoniflorin	0.083	326.5	360.9	337.9	351.8	369.1	382.2	354.7	5.76	20.43
	0.167	375.2	450.6	436.6	388.3	446.6	406.7	417.3	7.63	31.83
	0.25	465.9	437.0	438.3	453.9	476.0	500.6	462.0	5.26	24.32
	0.5	304.7	287.1	316.4	345.0	447.4	468.9	361.6	21.41	77.43
	0.75	235.6	222.2	227.8	193.0	210.7	281.9	228.6	13.15	30.06
	1	166.2	167.8	171.7	173.1	165.8	216.3	176.8	11.07	19.57
	2	137.1	122.7	137.8	95.8	99.0	132.0	120.7	15.64	18.89
	4	119.4	95.0	132.9	91.6	97.3	99.9	106.0	15.50	16.43
	6	44.71	38.55	86.31	44.01	34.75	80.31	54.77	41.05	22.49
	8	29.23	33.51	29.05	27.37	24.16	40.29	30.60	18.40	5.63
	12	12.93	13.13	11.31	12.15	14.96	15.61	13.35	12.33	1.65
	24	3.112	3.160	3.575	3.695	3.081	4.332	3.492	13.89	0.49
	36	ND	ND	ND	ND	ND	ND	—	—	—
Salvianolic acid B	0.083	143.7	136.0	150.7	148.6	169.6	156.9	150.9	7.65	11.54
	0.167	188.7	183.9	212.2	166.3	198.7	197.1	191.2	8.15	15.57
	0.25	391.1	352.9	454.9	281.9	392.3	367.8	373.5	15.21	56.80
	0.5	351.1	219.9	240.6	261.5	220.3	217.8	251.9	20.43	51.45
	0.75	117.5	96.88	82.52	90.88	79.76	84.96	92.07	15.07	13.87
	1	60.84	50.18	48.51	44.20	47.31	67.52	53.09	17.06	9.06
	2	36.91	32.19	36.06	23.93	34.35	30.06	32.25	14.84	4.79
	4	18.33	16.27	21.04	19.72	20.89	19.31	19.26	9.24	1.78
	6	12.39	10.54	8.47	12.58	14.51	12.47	11.83	17.50	2.07
	8	3.771	3.784	3.955	5.025	3.134	5.398	4.178	20.50	0.86
	12	ND	ND	ND	ND	ND	ND	—	—	—
Acteoside	0.083	3.325	3.219	3.045	4.014	4.167	3.292	3.510	13.17	0.46
	0.167	5.312	4.499	5.005	4.753	4.690	5.051	4.885	6.00	0.29
	0.25	10.59	10.02	10.98	10.63	10.75	10.66	10.61	2.98	0.32
	0.5	5.133	5.042	5.112	5.181	5.633	5.417	5.253	4.30	0.23
	0.75	4.302	4.247	4.211	4.418	4.572	4.189	4.323	3.40	0.15
	1	3.390	3.090	3.531	3.237	3.306	3.150	3.284	4.93	0.16
	2	2.544	2.432	2.377	2.171	2.325	2.366	2.369	5.20	0.12
	4	1.445	1.490	1.654	1.577	1.892	1.710	1.628	9.98	0.16
	6	1.135	1.176	1.309	1.412	1.264	1.314	1.268	7.92	0.10
	8	0.6925	0.6917	0.7031	0.7341	0.6981	0.7058	0.7042	2.23	0.02
	12	ND	ND	ND	ND	ND	ND	—	—	—
Tanshinone II A	0.083	28.68	29.91	27.91	29.27	30.57	30.22	29.43	3.41	1.00
	0.167	43.87	42.17	42.16	42.54	43.22	43.79	42.96	1.81	0.78
	0.25	76.35	74.77	74.60	75.41	75.15	75.17	75.24	0.82	0.62
	0.5	62.32	61.48	60.60	62.20	61.50	61.55	61.61	1.00	0.62
	0.75	57.06	56.91	58.57	59.72	58.17	58.72	58.19	1.83	1.07
	1	45.01	43.65	47.14	49.10	49.90	47.53	47.06	5.06	2.38
	2	18.67	20.12	20.96	19.73	22.07	21.68	20.54	6.21	1.28
	4	7.335	6.373	7.598	6.841	6.737	7.532	7.069	6.96	0.49
	6	4.260	3.820	5.420	4.324	4.967	5.470	4.710	14.37	0.68
	8	4.120	3.993	4.088	3.832	3.535	4.029	3.933	5.57	0.22
	12	2.705	3.143	2.488	2.019	2.219	2.554	2.521	15.52	0.39
	24	0.6933	0.7666	0.5351	0.7634	0.3847	0.4698	0.6021	26.84	0.16
	36	ND	ND	ND	ND	ND	ND	—	—	—

Figure 2 related data

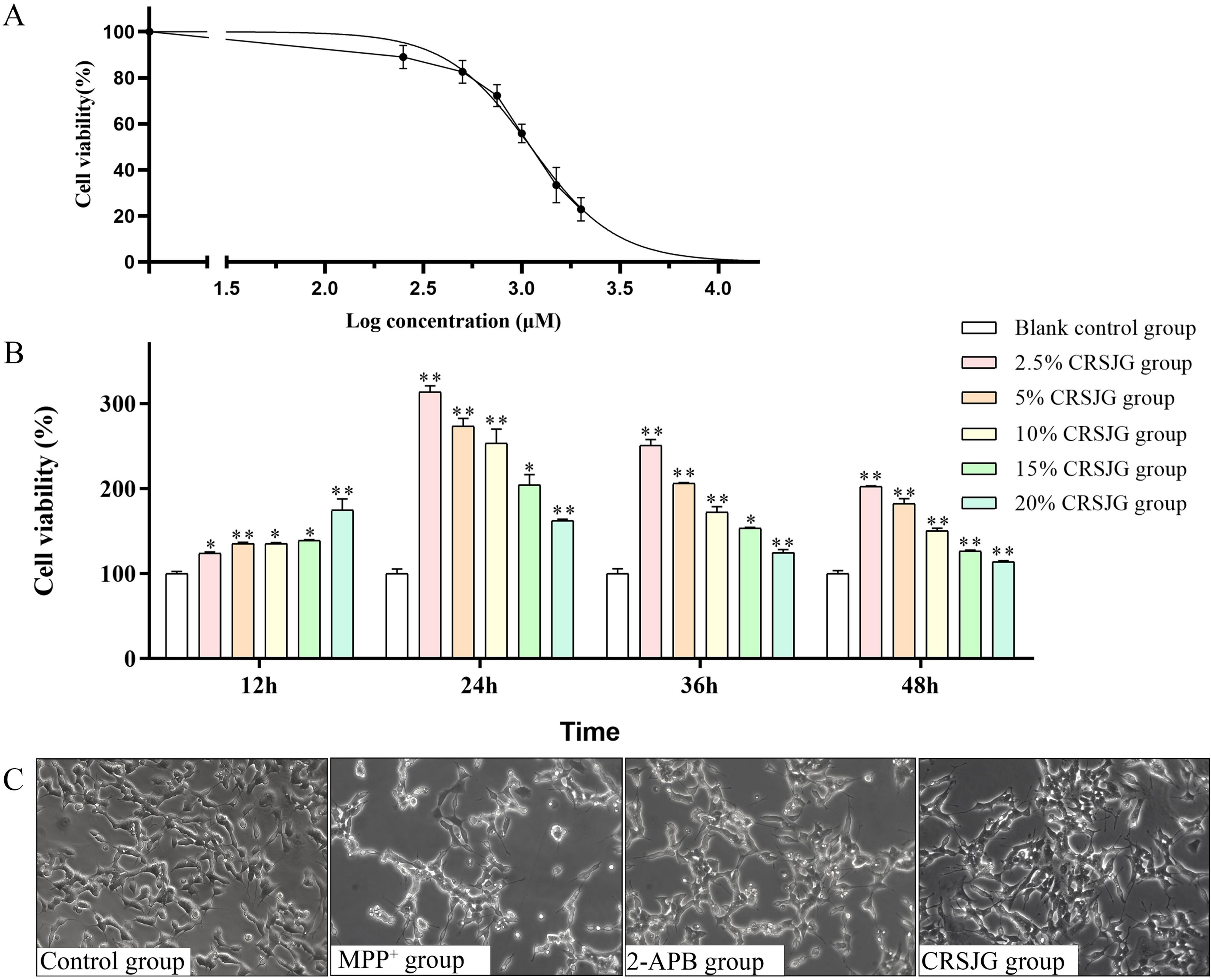

Figure 2. CRSJG drug-containing serum prevented MMP+-induced cell death.
(A) SH-SY5Y Cells were exposed to different concentration gradients of MMP+ in the range of 0-2000 μM, and MMP+ EC50 was calculated using GraphPad Prism 8.0 software by nonlinear regression of dose-response inhibition; (B)Protective effect of different concentrations of CRSJG containing serum on MMP+-induced cells for multiple times; (C) The cell morphology was observed by inverted fluorescence microscope after 24h intervention with 2.5% CRSJG drug-containing serum(×200). Results are showed as mean ± standard deviation (SD) from three independent experiments. M: * $P < 0.05$, ** $P < 0.01$ vs. 0 μM group or Blank control group.

Flow cytometry was used to detect the cyto-Ca^{2+} levels

mpp+ intervention concentration screening

Blank Group	mpp+ concentration 0uM	100μM	250μM	500μM	750μM	1000μM	1500μM	2000μM
0.29828	0.5223	0.52396	0.50493	0.47274	0.46921	0.41274	0.37756	0.35155
0.29861	0.51668	0.51789	0.50259	0.48107	0.46628	0.42107	0.40089	0.36238
0.29747	0.52874	0.52343	0.50557	0.49002	0.4718	0.44002	0.37091	0.34182
0.30546	0.53544	0.54386	0.50319	0.49465	0.4576	0.42465	0.37983	0.37079
0.29164	0.53645	0.51538	0.51647	0.51442	0.45507	0.43442	0.35028	0.33773
0.30241	0.53839	0.51075	0.49219	0.48465	0.47244	0.43465	0.37327	0.34397

Screening of intervention concentration of CRSJG-containing serum

Time	CRSJG-containing serum concentration 0%			2.50%			5%		
12h	0.77709	0.8113	0.73773	0.98007	0.94031	0.96026	1.038	1.0714	1.0433
24h	0.60846	0.58544	0.69657	1.8865	2.0001	2.0399	1.8098	1.7424	1.6142
36h	0.6298	0.74982	0.63384	1.6906	1.6009	1.7596	1.3666	1.3944	1.384
48h	0.7208	0.81263	0.77125	1.5668	1.5497	1.5479	1.3448	1.3754	1.4859
**Time**	**CRSJG-containing serum concentration 10%**			**15%**			**20%**		
12h	1.0551	1.05891	1.03915	1.0766	1.0705	1.08801	1.1521	1.456	1.456
24h	1.7479	1.6457	1.3979	1.4364	1.2295	1.1942	1.0447	1.0043	1.0149
36h	1.0725	1.215	1.1829	1.026	1.0398	1.0244	0.85901	0.86487	0.79176
48h	1.1159	1.1693	1.1876	0.9794	0.9831	0.9569	0.85832	0.88702	0.8812

Control group

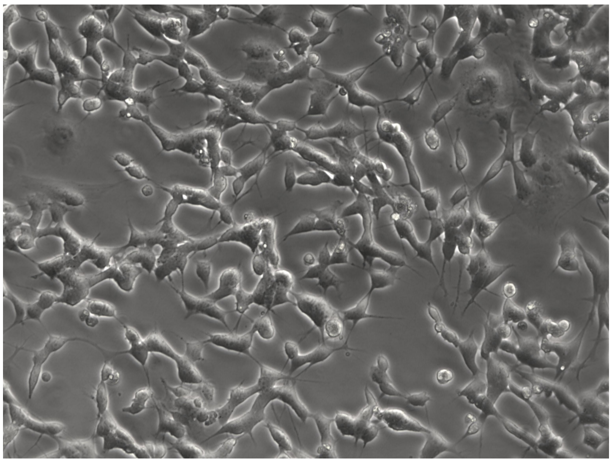

MMP+ group

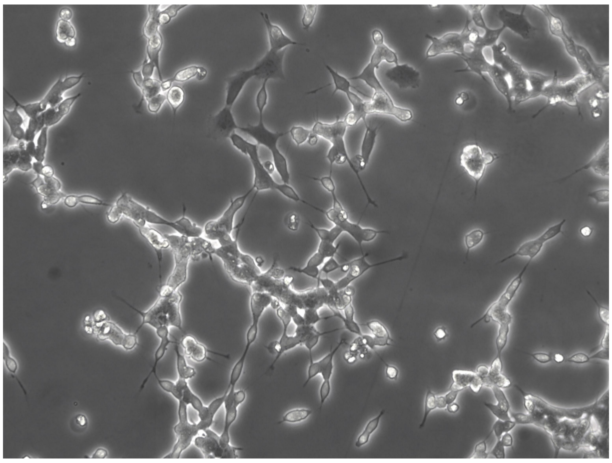

2-APB group

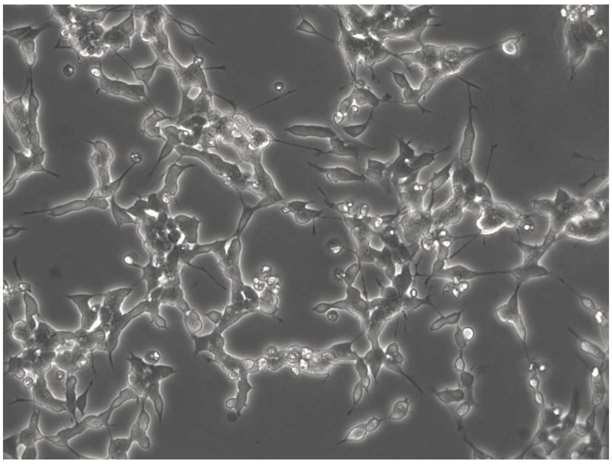

CRSJG group

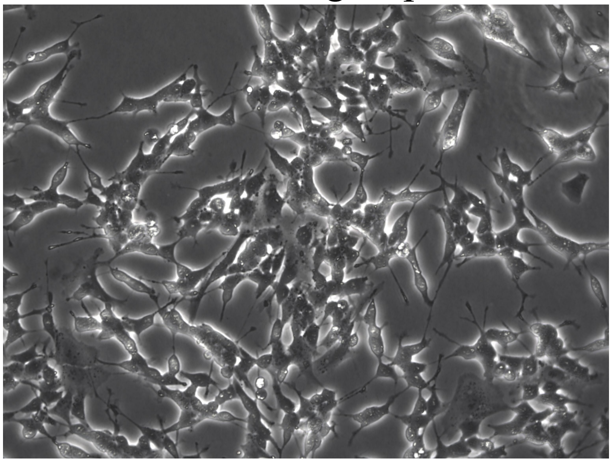

Figure 3 related data

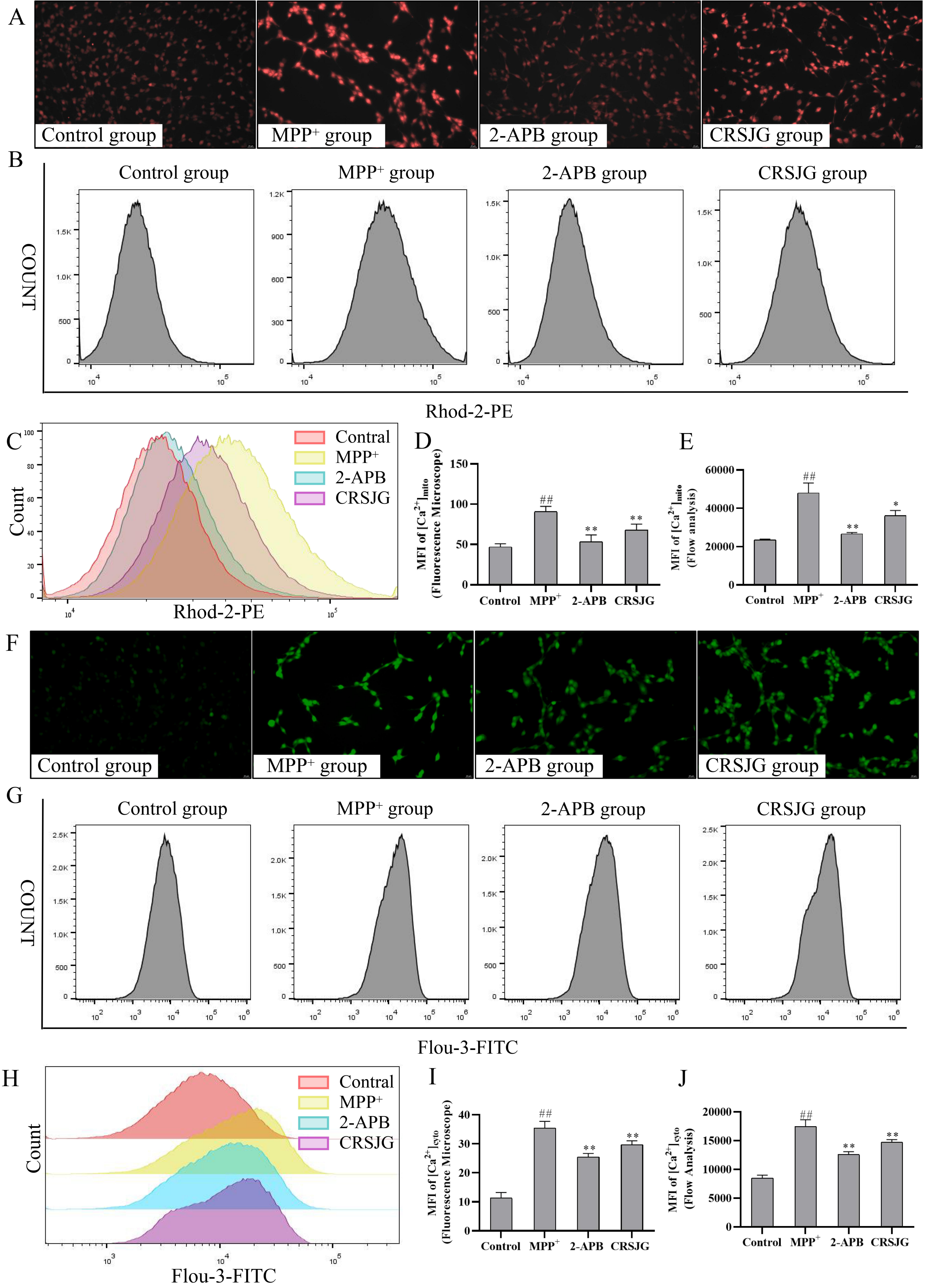

Figure 3. CRSJG regulates intracellular Ca^{2+} homeostasis to relieve mitochondrial Ca^{2+} overload. (A, D) Rhod-2 fluorescence probe was used to detect the mito-Ca^{2+} levels in each group, and the quantitative results of MFI; (B, C, E) Flow cytometry was used to detect the mito-Ca^{2+} levels, and the quantitative results of MFI; (F, I) Flou-3 fluorescence probe was used to detect the cyto-Ca^{2+} levels in each group, and the quantitative results of MFI ; (G, H, F) Flow cytometry was used to detect the cyto-Ca^{2+} levels, and the quantitative results of MFI. Results are showed as mean ± standard deviation (SD), of which all results are from four independent experiments. M: # $P < 0.05$, ##$P < 0.01$ vs. control group; * $P < 0.05$, ** $P < 0.01$ vs. MMP^+ group.

Gating strategy of mito/cyto-Ca^{2+} analysis

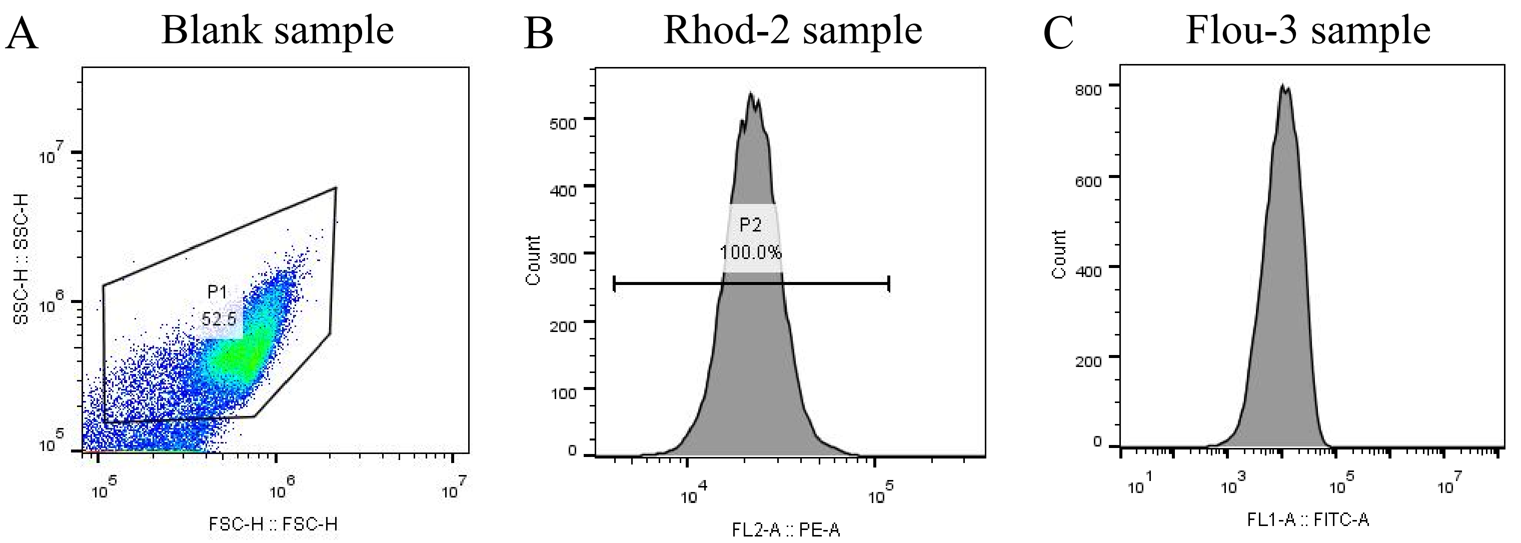

In the analysis of intracellular Ca^{2+} stained by Rhod-2 and Flou-3, we adopted the following gating strategy:

A: In the blank sample, we used SSA-H and FSC-H to select the aggregated cells.

B: In the Rhod-2 stained sample, we used the PE-A histogram to determine the fluorescence intensity of Rhod-2.

C: In Flou-3 stained samples, we used the FITC-A histogram to determine the fluorescence value intensity of Flou-3.

In the experiment, there were significant differences in Ca^{2+} staining of the samples in each group. We used the MFI of PE-A for late statistical analysis of mito-Ca^{2+} levels and the MFI of FITC-A for late statistical analysis of cyto-Ca^{2+} levels.

Rhod-2 fluorescence probe was used to detect the mito-Ca^{2+} levels

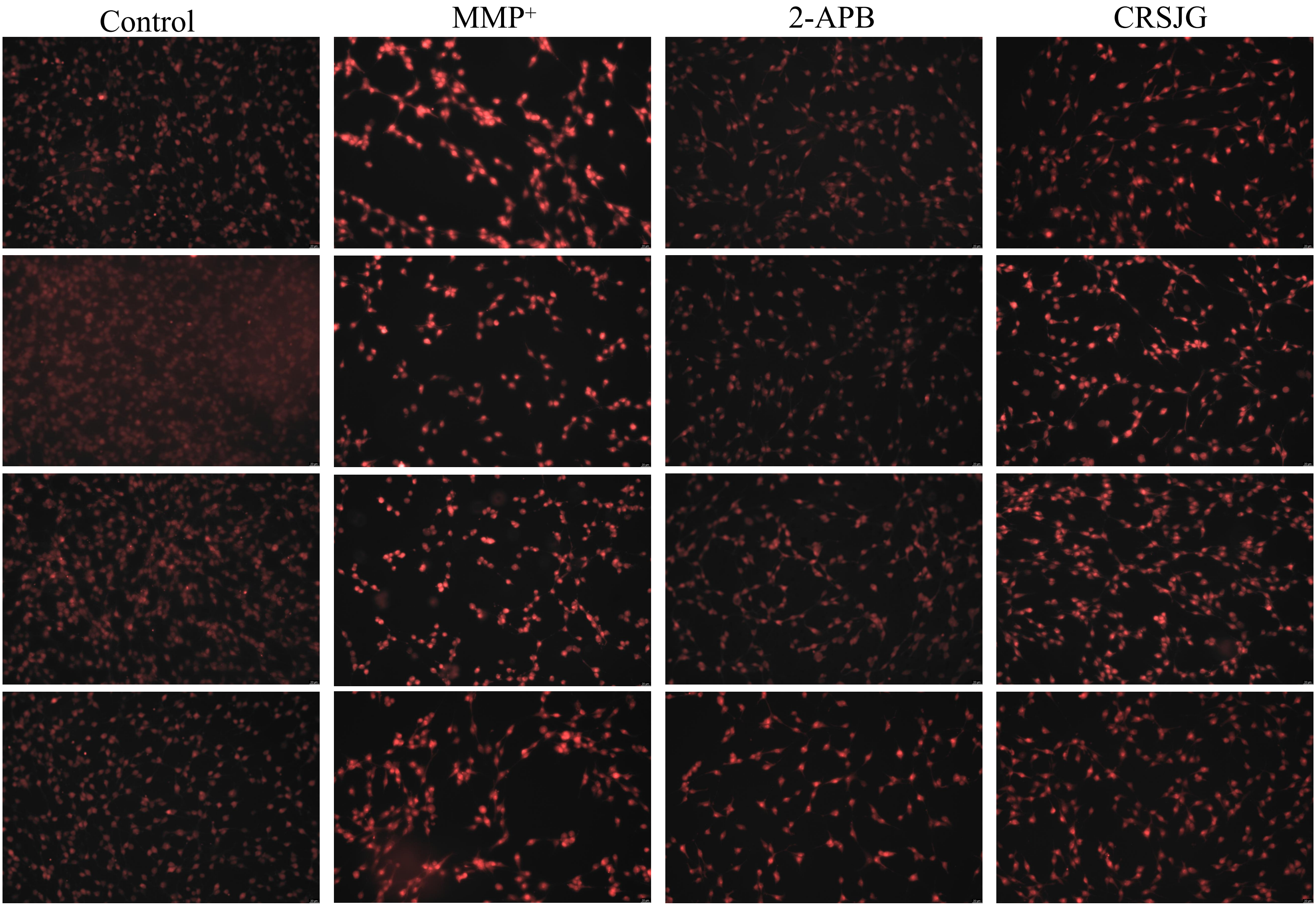

	Area	Mean	StdDev	IntDen	Median	%Area	RawIntDen	MinThr	MaxThr
Control-1	779848	47.588	7.693	37111401	46	15.627	37111401	43	255
Control-2	780563	51.739	11.732	40385278	49	15.641	40385278	41	255
Control-3	1032975	42.352	13.506	43748426	40	20.699	43748426	27	255
Control-4	924087	46.545	12.977	43011899	43	18.517	43011899	33	255
MPP+-1	510366	100.214	26.344	51146060	95	10.227	51146060	65	255
MPP+-2	329478	84.95	27.859	27989108	78	6.602	27989108	53	255
MPP+-3	329283	87.718	24.194	28884055	82	6.598	28884055	59	255
MPP+-4	256778	89.862	20.505	23074659	84	5.145	23074659	68	255
2-APB-1	496574	46.397	13.661	23039527	43	9.95	23039527	33	255
2-APB-2	556011	50.625	13.805	28147821	47	11.141	28147821	37	255
2-APB-3	594203	51.117	14.333	30373792	48	11.907	30373792	36	255
2-APB-4	338324	65.631	20.985	22204505	61	6.779	22204505	42	255
CRSJG-1	433381	63.779	21.501	27640665	58	8.684	27640665	41	255
CRSJG-2	517422	76.063	26.408	39356420	70	10.368	39356420	43	255
CRSJG-3	679207	71.581	20.858	48618007	67	13.61	48618007	47	255
CRSJG-4	556735	59.601	18.331	33182094	55	11.156	33182094	40	255

Flow cytometry was used to detect the mito-Ca^{2+} levels

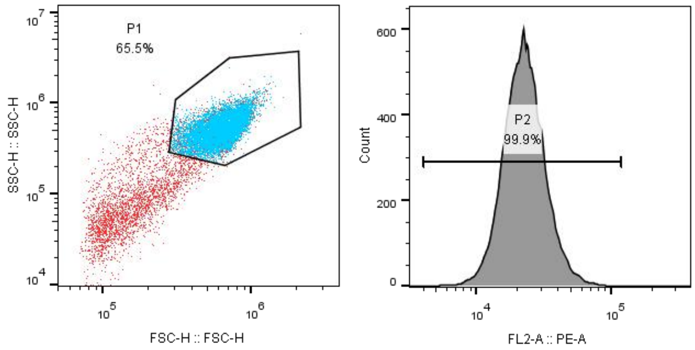

	样本名称	亚群名称	Count
	Z-5.fcs	P1	26181
	Z-5.fcs	未设门的	40000

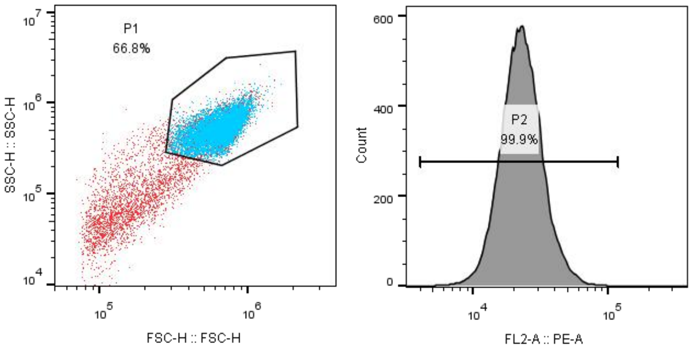

	样本名称	亚群名称	Count
	Z-4.fcs	P1	26705
	Z-4.fcs	未设门的	40000

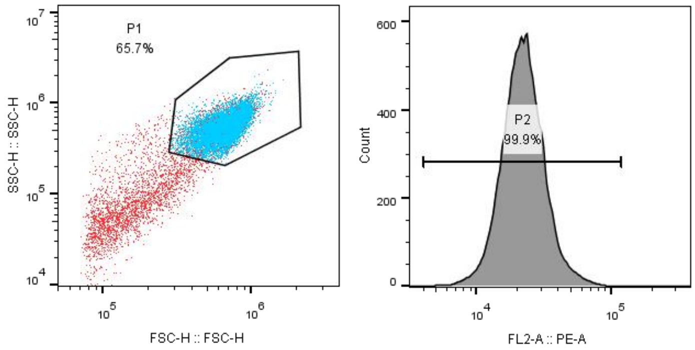

	样本名称	亚群名称	Count
	Z-3.fcs	P1	26274
	Z-3.fcs	未设门的	40000

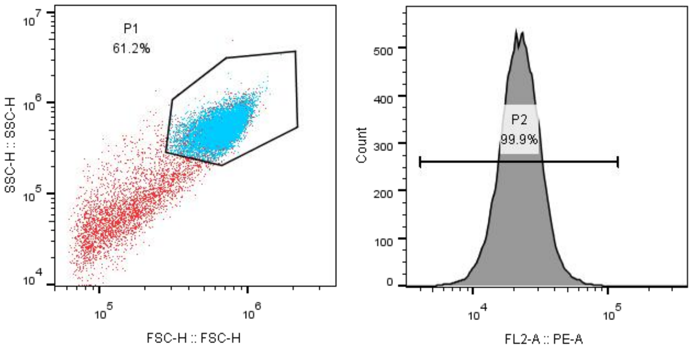

	样本名称	亚群名称	Count
	Z-2.fcs	P1	24493
	Z-2.fcs	未设门的	40000

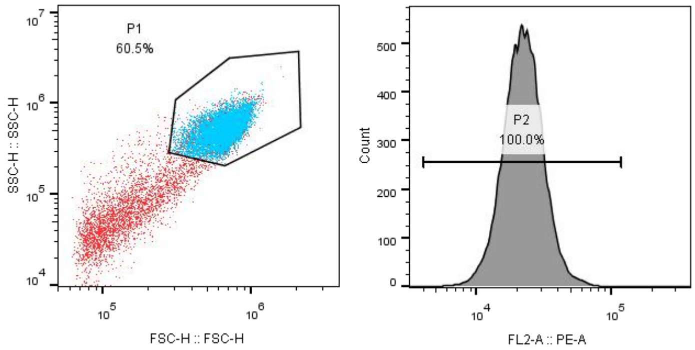

	样本名称	亚群名称	Count
	Z-1.fcs	P1	24192
	Z-1.fcs	未设门的	40000

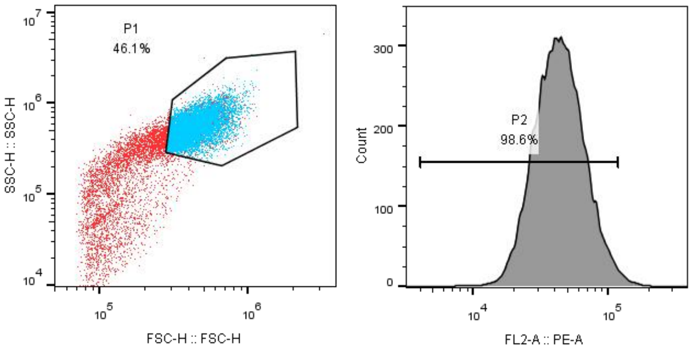

	样本名称	亚群名称	Count
	M-5.fcs	P1	18459
	M-5.fcs	未设门的	40000

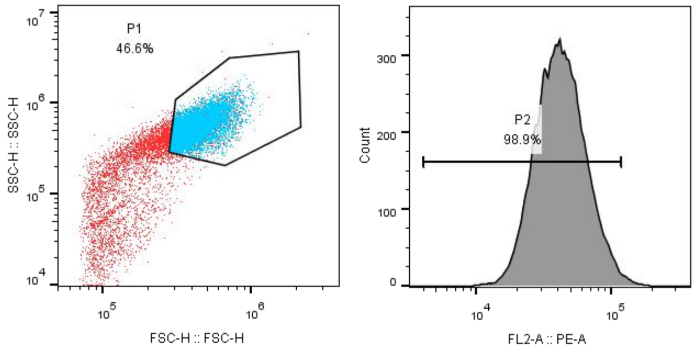

	样本名称	亚群名称	Count
	M-4.fcs	P1	18645
	M-4.fcs	未设门的	40000

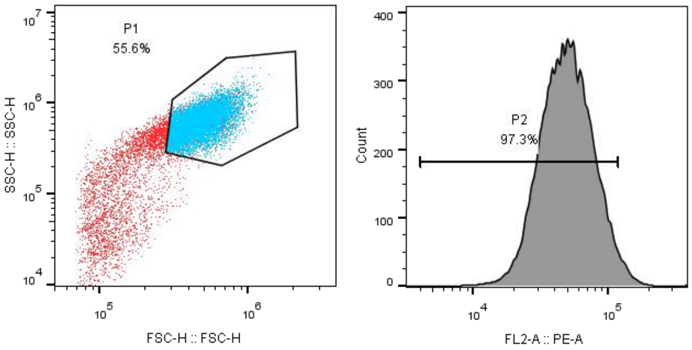

	样本名称	亚群名称	Count
	M-3.fcs	P1	22256
	M-3.fcs	未设门的	40000

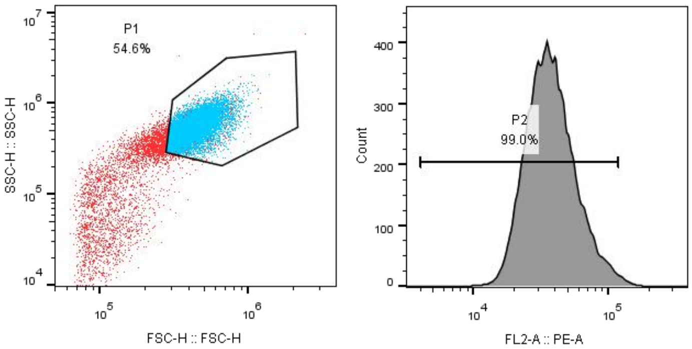

	样本名称	亚群名称	Count
	M-2.fcs	P1	21821
	M-2.fcs	未设门的	40000

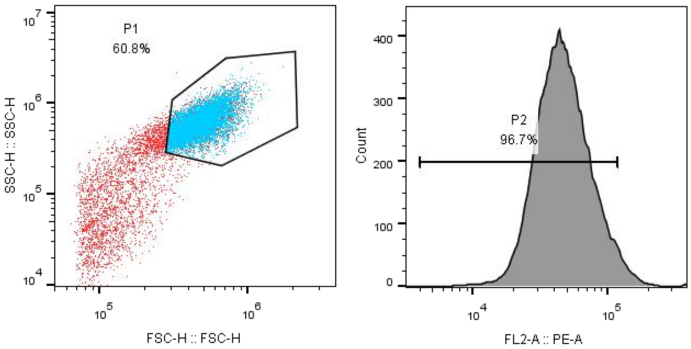

	样本名称	亚群名称	Count
	M-1.fcs	P1	24309
	M-1.fcs	未设门的	40000

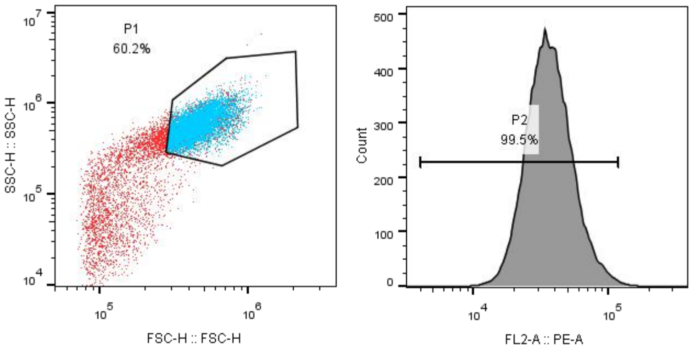

	样本名称	亚群名称	Count
	C-5.fcs	P1	24089
	C-5.fcs	未设门的	40000

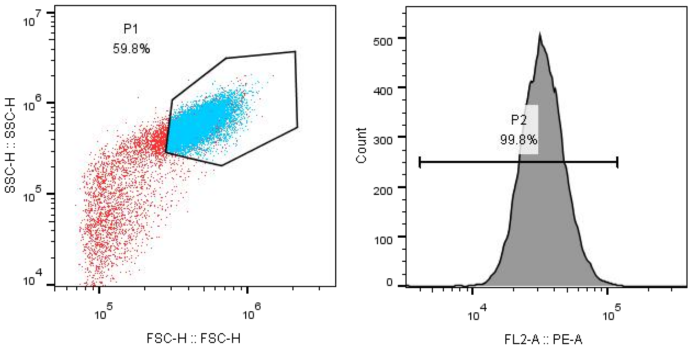

	样本名称	亚群名称	Count
	C-4.fcs	P1	23913
	C-4.fcs	未设门的	40000

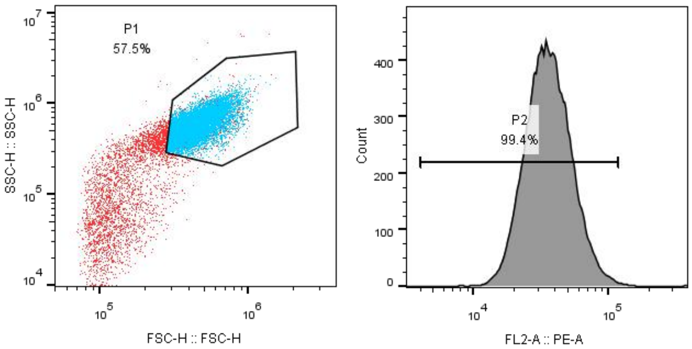

	样本名称	亚群名称	Count
	C-3.fcs	P1	23010
	C-3.fcs	未设门的	40000

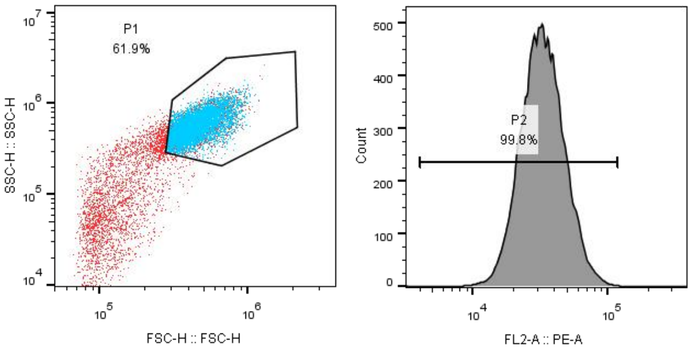

	样本名称	亚群名称	Count
	C-2.fcs	P1	24765
	C-2.fcs	未设门的	40000

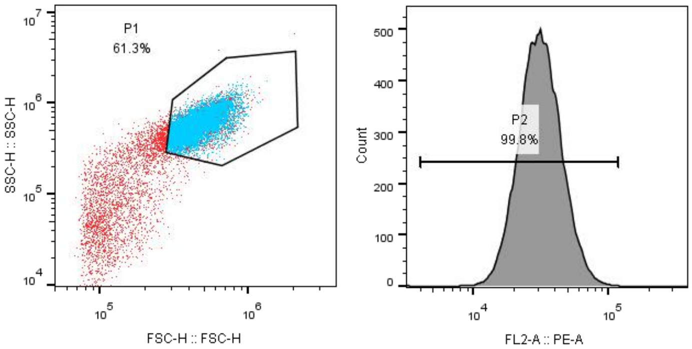

	样本名称	亚群名称	Count
	C-1.fcs	P1	24500
	C-1.fcs	未设门的	40000

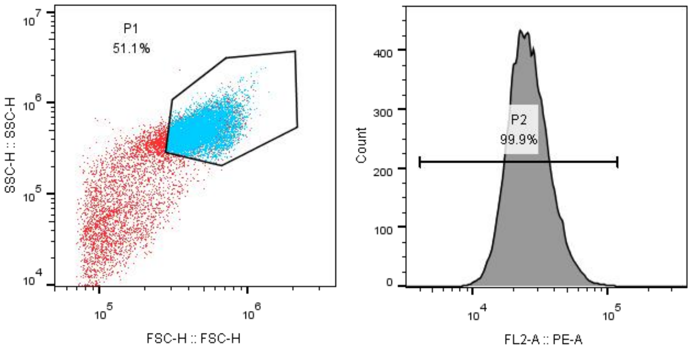

	样本名称	亚群名称	Count
	2-APB-5.fcs	P1	20426
	2-APB-5.fcs	未设门的	40000

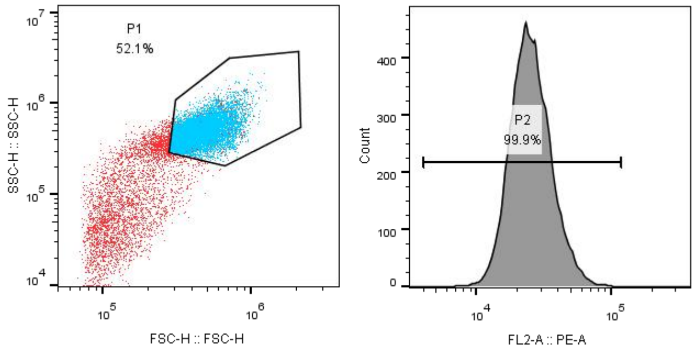

	样本名称	亚群名称	Count
	2-APB-4.fcs	P1	20824
	2-APB-4.fcs	未设门的	40000

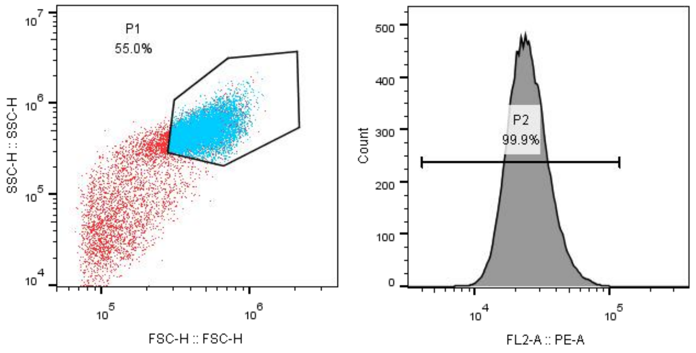

	样本名称	亚群名称	Count
	2-APB-3.fcs	P1	22014
	2-APB-3.fcs	未设门的	40000

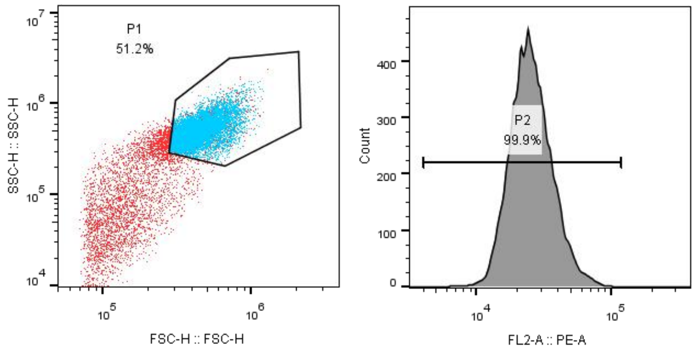

	样本名称	亚群名称	Count
	2-APB-2.fcs	P1	20460
	2-APB-2.fcs	未设门的	40000

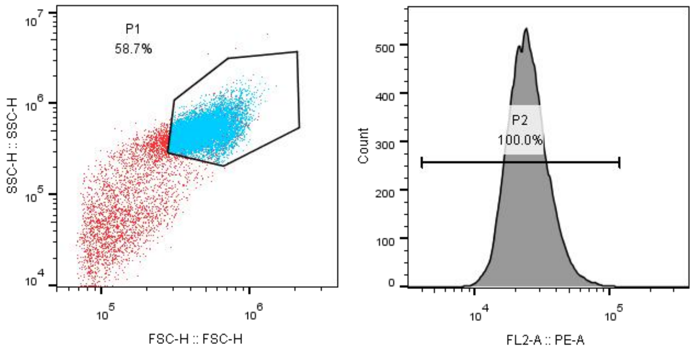

	样本名称	亚群名称	Count
	2-APB-1.fcs	P1	23479
	2-APB-1.fcs	未设门的	40000

Ancestry Subset Statistic For	未设门的 Count	未设门的 Freq. of Total	P1 Count	P1 Freq. of Total	P1 Freq. of Parent	P1 Mean PE-A	P1 Median PE-A
Z-5.fcs	40000	100%	26181	65.5%	65.5%	23853	22114
Z-4.fcs	40000	100%	26705	66.8%	66.8%	24090	22400
Z-3.fcs	40000	100%	26274	65.7%	65.7%	23482	21730
Z-2.fcs	40000	100%	24493	61.2%	61.2%	23365	21934
Z-1.fcs	40000	100%	24192	60.5%	60.5%	23191	21832
M-5.fcs	40000	100%	18459	46.1%	46.1%	47365	42702
M-4.fcs	40000	100%	18645	46.6%	46.6%	45481	40943
M-3.fcs	40000	100%	22256	55.6%	55.6%	54030	48675
M-2.fcs	40000	100%	21821	54.6%	54.6%	40862	35919
M-1.fcs	40000	100%	24309	60.8%	60.8%	52114	45272
C-5.fcs	40000	100%	24089	60.2%	60.2%	39134	35502
C-4.fcs	40000	100%	23913	59.8%	59.8%	34977	32295
C-3.fcs	40000	100%	23010	57.5%	57.5%	38830	35130
C-2.fcs	40000	100%	24765	61.9%	61.9%	34752	32182
C-1.fcs	40000	100%	24500	61.3%	61.3%	32950	30604
2-APB-5.fcs	40000	100%	20426	51.1%	51.1%	27527	25060
2-APB-4.fcs	40000	100%	20824	52.1%	52.1%	26839	24538
2-APB-3.fcs	40000	100%	22014	55.0%	55.0%	26167	23971
2-APB-2.fcs	40000	100%	20460	51.2%	51.2%	26964	24596
2-APB-1.fcs	40000	100%	23479	58.7%	58.7%	26022	23860
Mean	*40000*	*100%*	*23041*	*57.6%*	*57.6%*	*33600*	*30563*
SD	*0*	*0*	*2382*	*5.96*	*5.96*	*10119*	*8695*

Flou-3 fluorescence probe was used to detect the cyto-Ca^{2+} levels

Control MMP+ 2-APB CRSJG

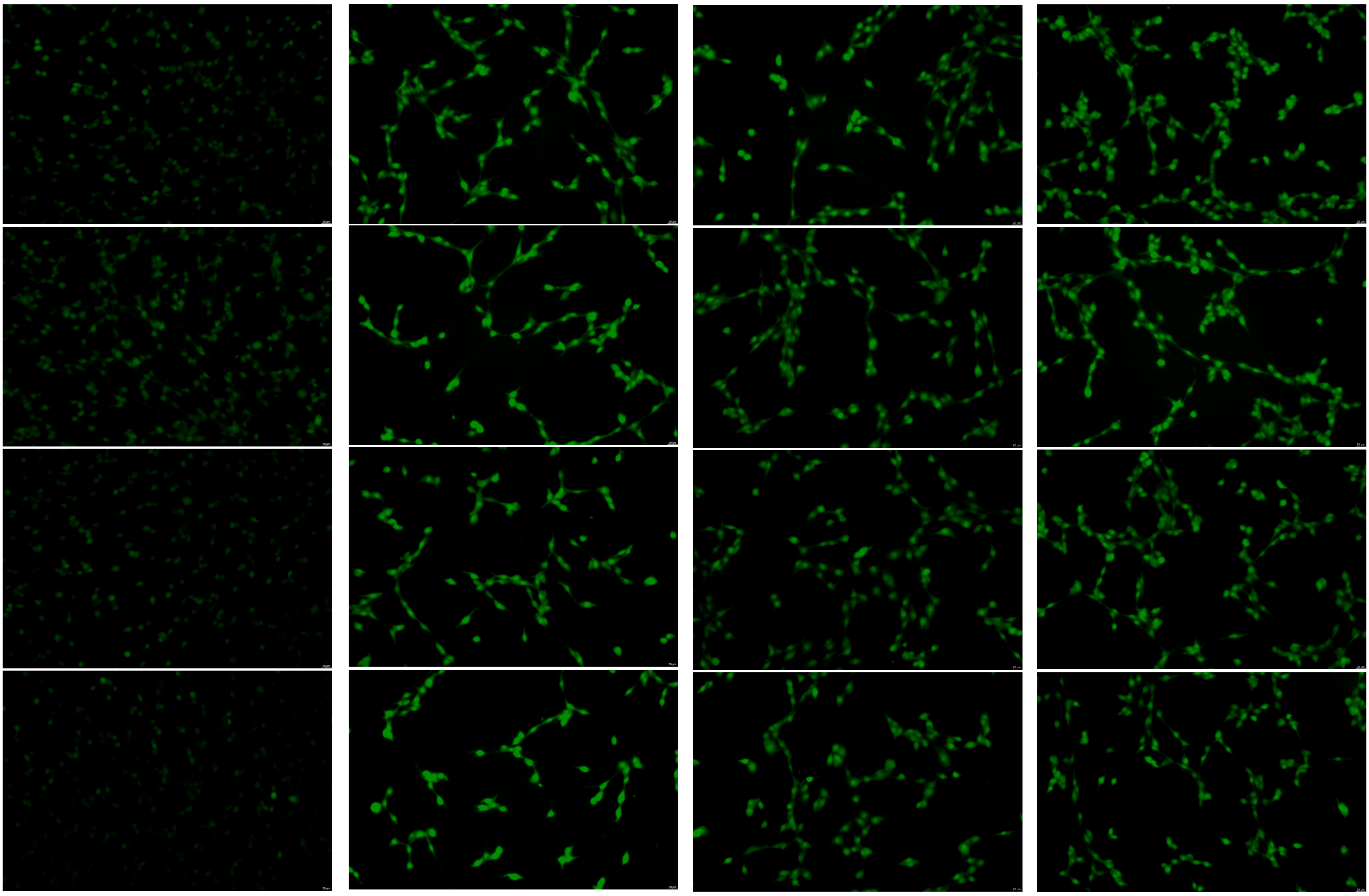

	Area	Mean	StdDev	IntDen	Median	%Area	RawIntDer	MinThr	MaxThr
Control-1	341812	12. 631	9. 874	4317562	11	6. 849	4317562	8	255
Control-2	624782	12. 91	7. 788	8066185	12	12. 52	8066185	8	255
Control-3	264412	10. 994	10. 759	2906835	9	5. 298	2906835	7	255
Control-4	227286	8. 774	11. 773	1994171	7	4. 554	1994171	5	255
MPP+-1	289386	33. 182	12. 102	9602329	31	5. 799	9602329	21	255
MPP+-2	268993	38. 408	12. 884	10331552	40	5. 39	10331552	21	255
MPP+-3	334555	34. 415	11. 224	11513582	33	6. 704	11513582	23	255
MPP+-4	338176	35. 841	12. 395	12120726	35	6. 776	12120726	20	255
2-APB-1	400358	25. 82	10. 617	10337224	24	8. 022	10337224	16	255
2-APB-2	343233	24. 168	11. 261	8295269	22	6. 878	8295269	15	255
2-APB-3	331894	27. 007	11. 322	8963614	25	6. 651	8963614	17	255
2-APB-4	534530	24. 736	12. 77	13222290	22	10. 711	13222290	11	255
CRSJG-1	514558	30. 565	11. 578	15727423	29	10. 311	15727423	17	255
CRSJG-2	405326	28. 433	10. 921	11524520	27	8. 122	11524520	18	255
CRSJG-3	320950	28. 707	11. 657	9213610	27	6. 431	9213610	18	255
CRSJG-4	528218	31. 071	10. 184	16412080	30	10. 585	16412080	20	255

Flow cytometry was used to detect the cyto-Ca^{2+} levels

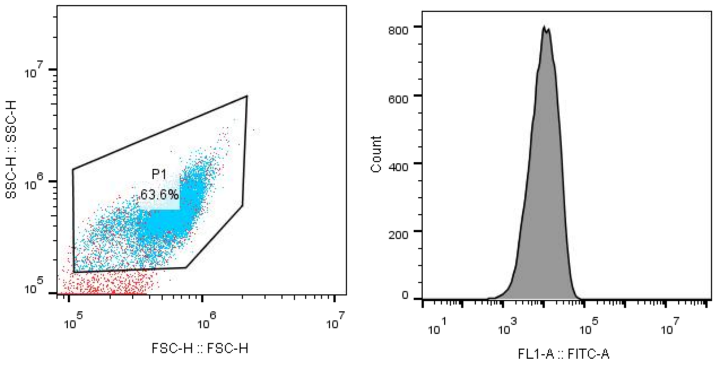

	样本名称	亚群名称	Count
	2-APB-1.fcs	P1	25429
	2-APB-1.fcs	未设门的	40000

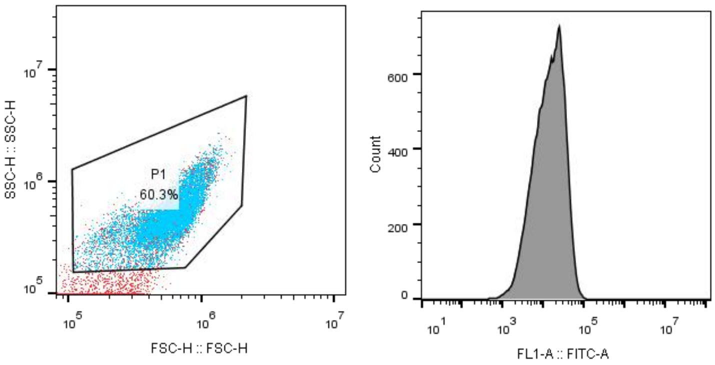

	样本名称	亚群名称	Count
	2-APB-2.fcs	P1	24129
	2-APB-2.fcs	未设门的	40000

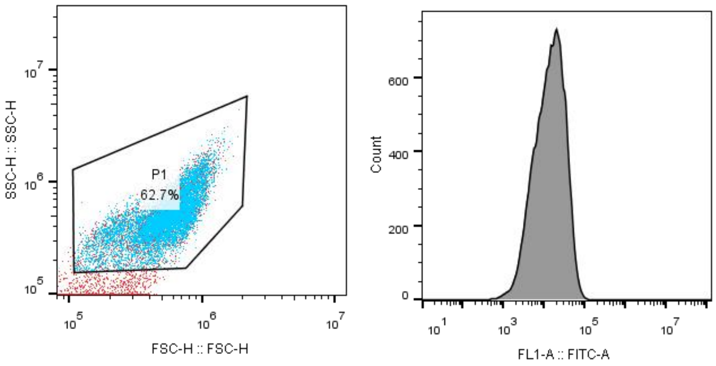

	样本名称	亚群名称	Count
	2-APB-3.fcs	P1	25073
	2-APB-3.fcs	未设门的	40000

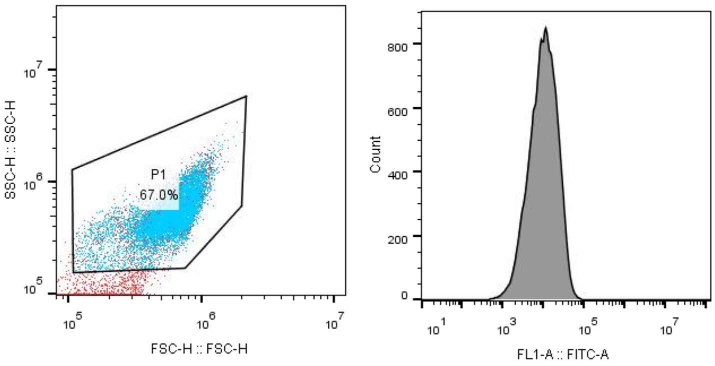

	样本名称	亚群名称	Count
	2-APB-4.fcs	P1	26815
	2-APB-4.fcs	未设门的	40000

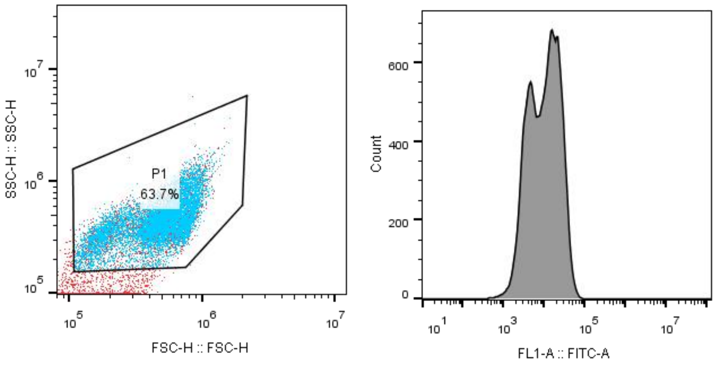

	样本名称	亚群名称	Count
	2-APB-5.fcs	P1	25469
	2-APB-5.fcs	未设门的	40000

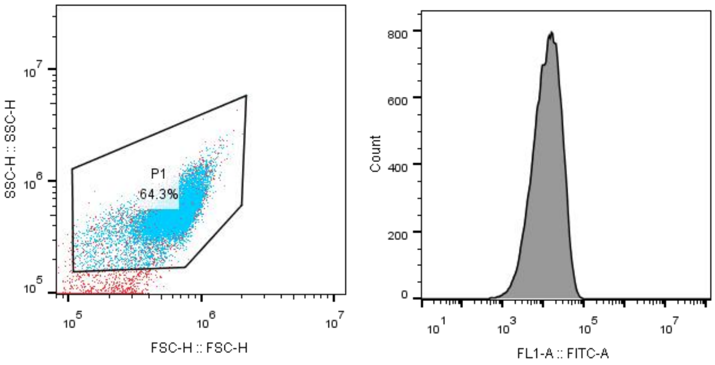

	样本名称	亚群名称	Count
	C-1.fcs	P1	25705
	C-1.fcs	未设门的	40000

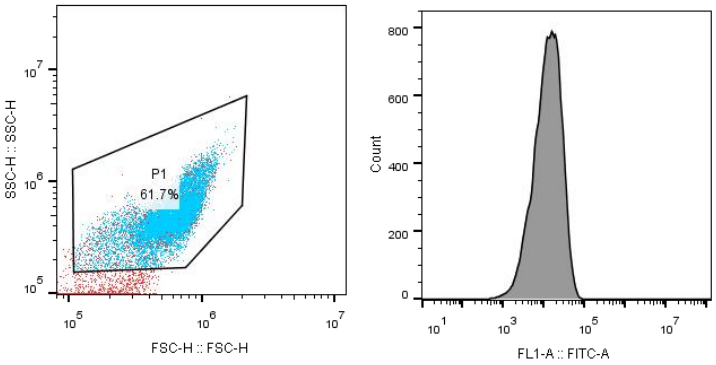

	样本名称	亚群名称	Count
	C-2.fcs	P1	24681
	C-2.fcs	未设门的	40000

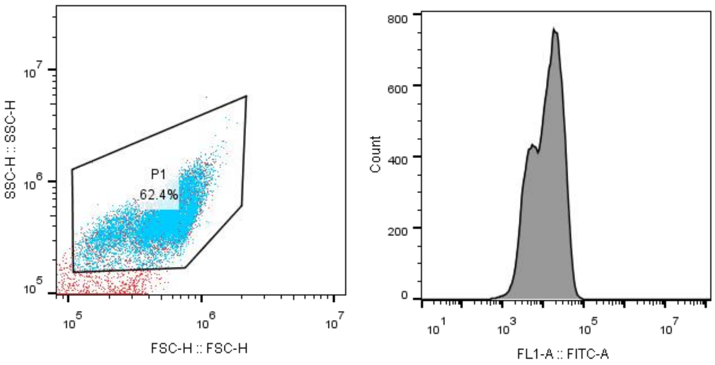

	样本名称	亚群名称	Count
	C-3.fcs	P1	24955
	C-3.fcs	未设门的	40000

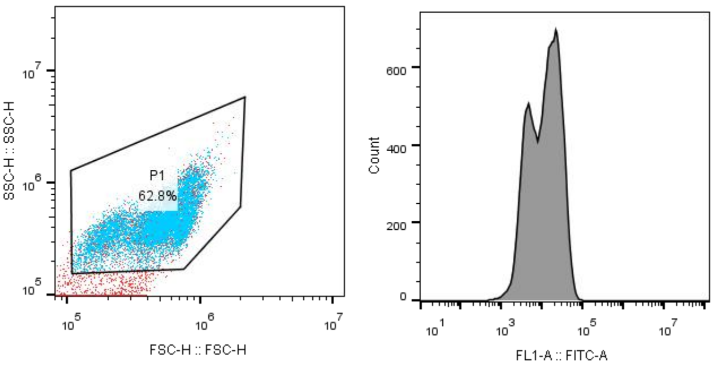

	样本名称	亚群名称	Count
	C-4.fcs	P1	25105
	C-4.fcs	未设门的	40000

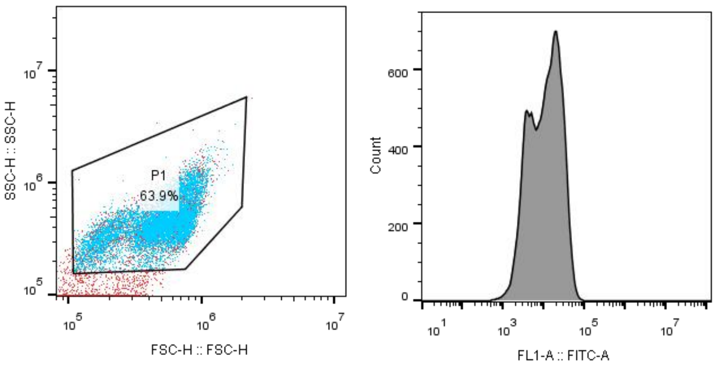

	样本名称	亚群名称	Count
	C-5.fcs	P1	25544
	C-5.fcs	未设门的	40000

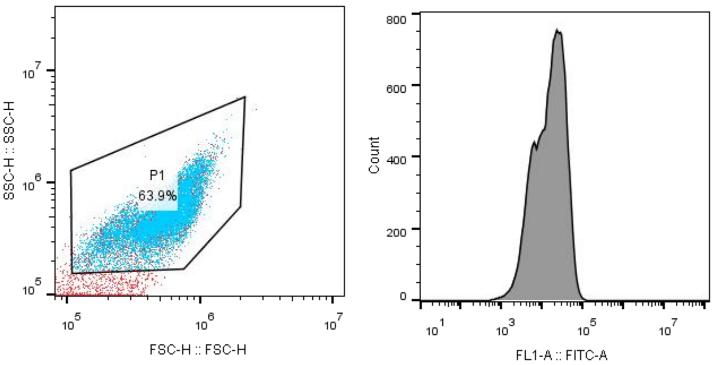

	样本名称	亚群名称	Count
	M-1.fcs	P1	25560
	M-1.fcs	未设门的	40000

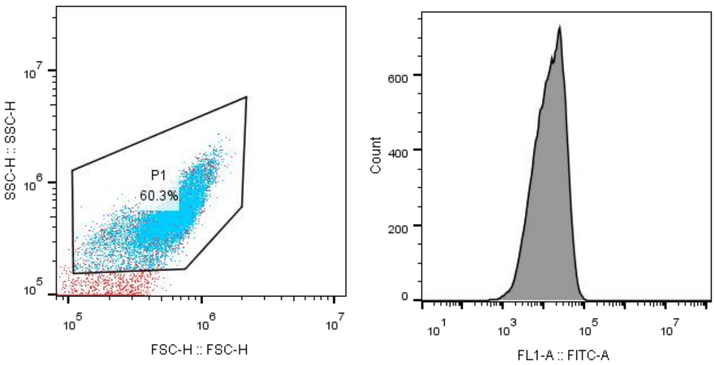

	样本名称	亚群名称	Count
	M-2.fcs	P1	24129
	M-2.fcs	未设门的	40000

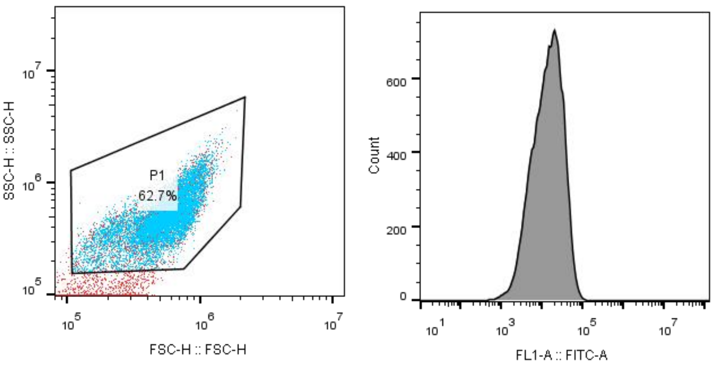

	样本名称	亚群名称	Count
	M-3.fcs	P1	25073
	M-3.fcs	未设门的	40000

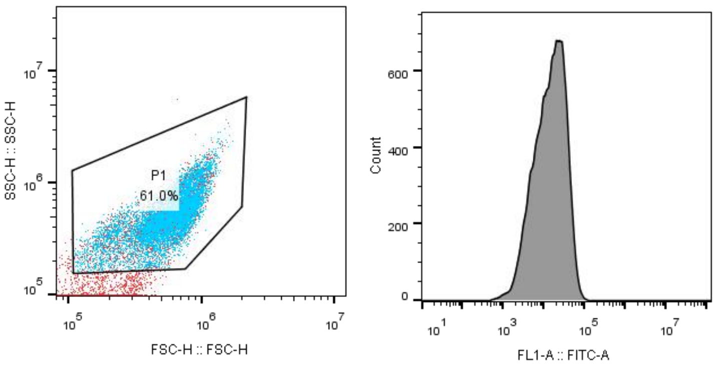

	样本名称	亚群名称	Count
	M-4.fcs	P1	24385
	M-4.fcs	未设门的	40000

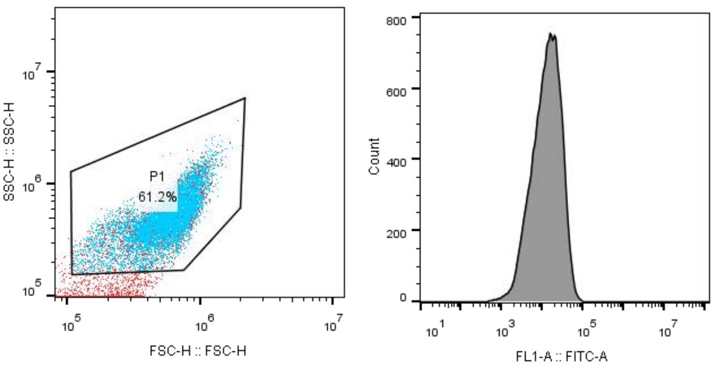

	样本名称	亚群名称	Count
	M-5.fcs	P1	24478
	M-5.fcs	未设门的	40000

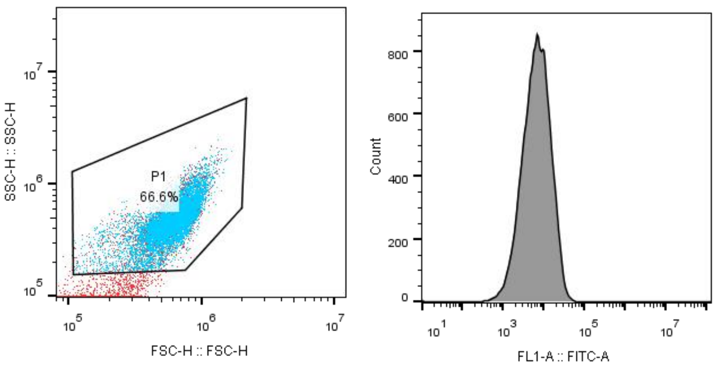

	样本名称	亚群名称	Count
	Z-1.fcs	P1	26639
	Z-1.fcs	未设门的	40000

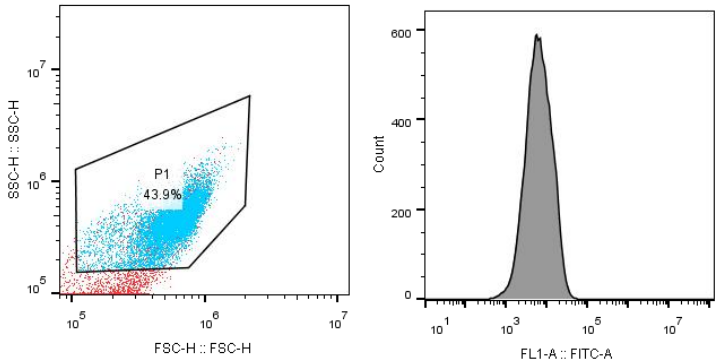

	样本名称	亚群名称	Count
	Z-2.fcs	P1	17570
	Z-2.fcs	未设门的	40000

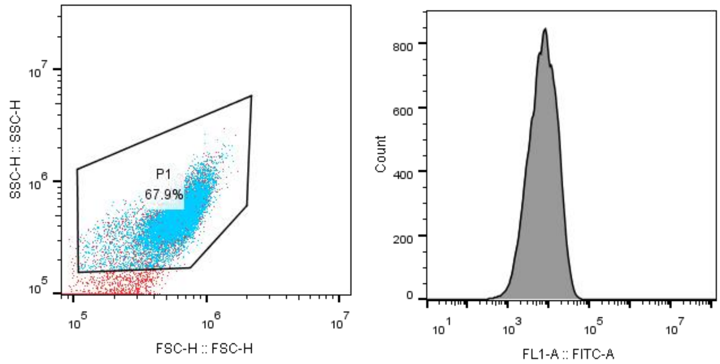

	样本名称	亚群名称	Count
	Z-3.fcs	P1	27145
	Z-3.fcs	未设门的	40000

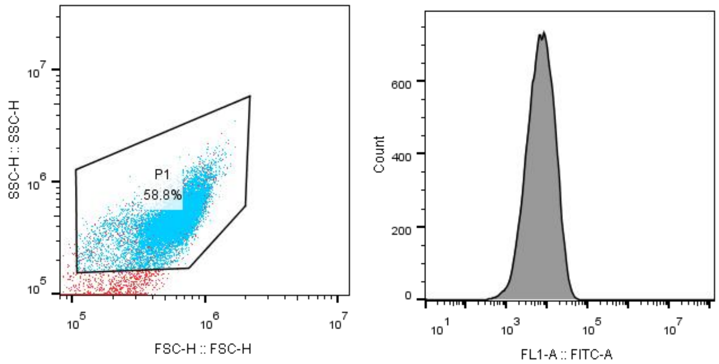

	样本名称	亚群名称	Count
	Z-4.fcs	P1	23534
	Z-4.fcs	未设门的	40000

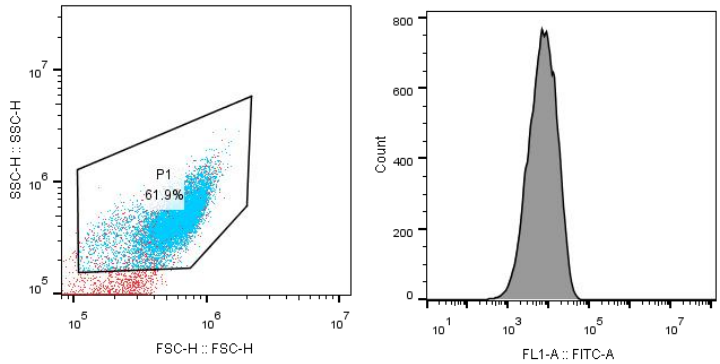

	样本名称	亚群名称	Count
	Z-5.fcs	P1	24766
	Z-5.fcs	未设门的	40000

			P1 *Count*	P1 *Freq. of Total*	P1 *Freq. of Parent*	P1 *Mean FITC-A*	P1 *Median FITC-A*
Control-1	40000	100	26639	66.6	66.6	8178	6537
Control-2	40000	100	17570	43.9	43.9	7719	6087
Control-3	40000	100	27145	67.9	67.9	8955	7102
Control-4	40000	100	23534	58.8	58.8	8644	6863
Control-5	40000	100	23534	58.8	58.8	8644	6863
MPP+-1	40000	100	25560	63.9	63.9	19080	15979
MPP+-2	40000	100	24129	60.3	60.3	17250	14012
MPP+-3	40000	100	25073	62.7	62.7	17228	13853
MPP+-4	40000	100	24385	61	61	17936	14542
MPP+-5	40000	100	24478	61.2	61.2	15893	13121
2-APB-1	40000	100	25429	63.6	63.6	12139	9890
2-APB-2	40000	100	26275	65.7	65.7	12375	9947
2-APB-3	40000	100	24164	60.4	60.4	13203	10744
2-APB-4	40000	100	26815	67	67	12301	9919
2-APB-5	40000	100	25469	63.7	63.7	13044	10235
CRSJG-1	40000	100	25705	64.3	64.3	15035	12358
CRSJG-2	40000	100	24681	61.7	61.7	14852	12428
CRSJG-3	40000	100	24955	62.4	62.4	15178	12679
CRSJG-4	40000	100	25105	62.8	62.8	14738	11840
CRSJG-5	40000	100	25544	63.9	63.9	14057	11024
Mean	40000	100	24871	62.2	62.2	13336	10813
SD	0	0	1960	4.91	4.91	3439	2922

Figure 4 related data

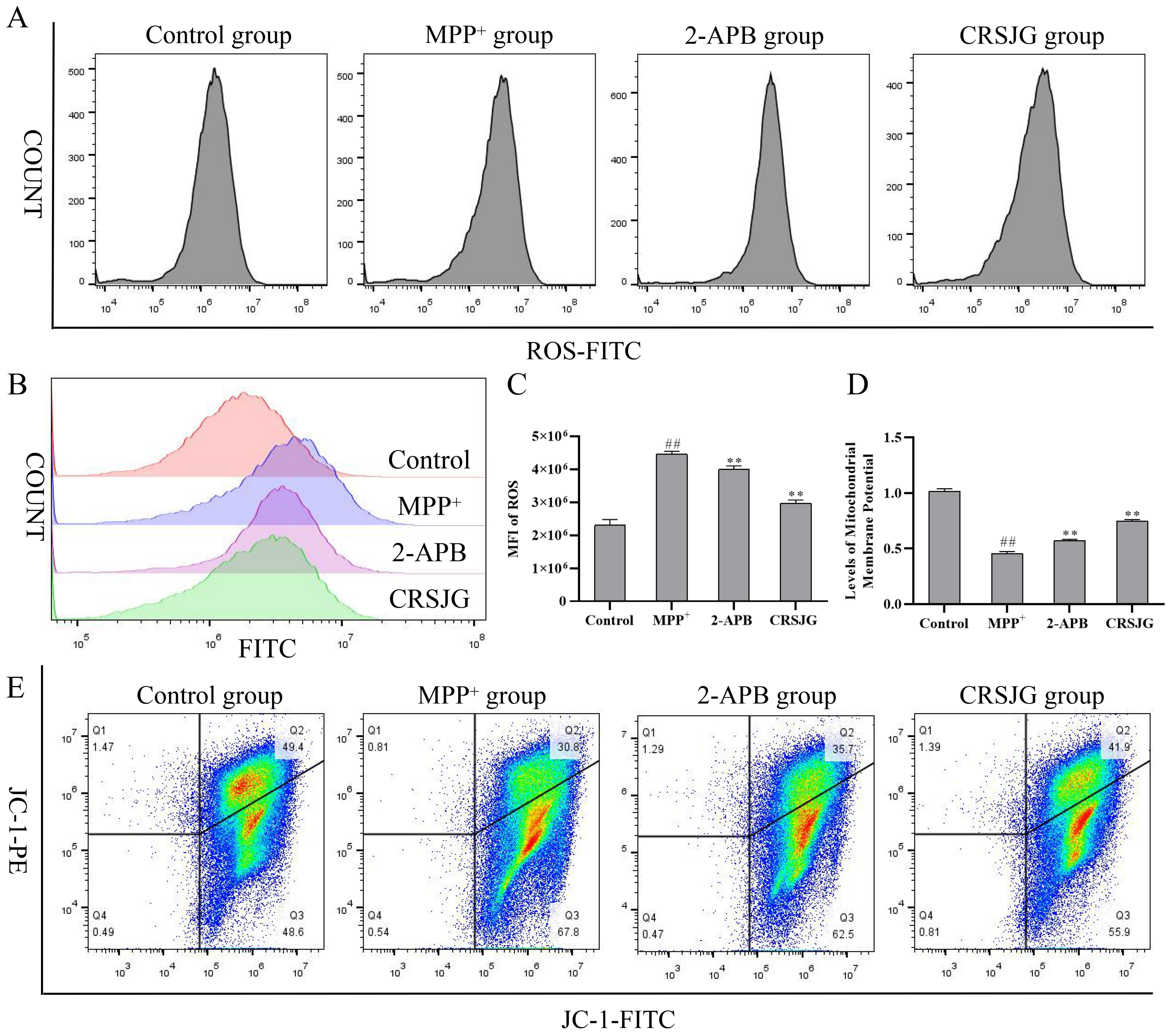

Figure 4. CRSJG improved the mitochondrial membrane potential reduction and ROS increase caused by MMP+. (A-C) Flow cytometry was used to detect ROS levels, and the quantitative results of MFI; (D, E) Flow cytometry was used to detect MMP. Results are showed as mean ± standard deviation (SD), of which all results are from four independent experiments. M: # $P < 0.05$, ##$P < 0.01$ vs. control group; * $P < 0.05$, ** $P < 0.01$ vs. MMP+ group.

Gating strategy of ROS analysis

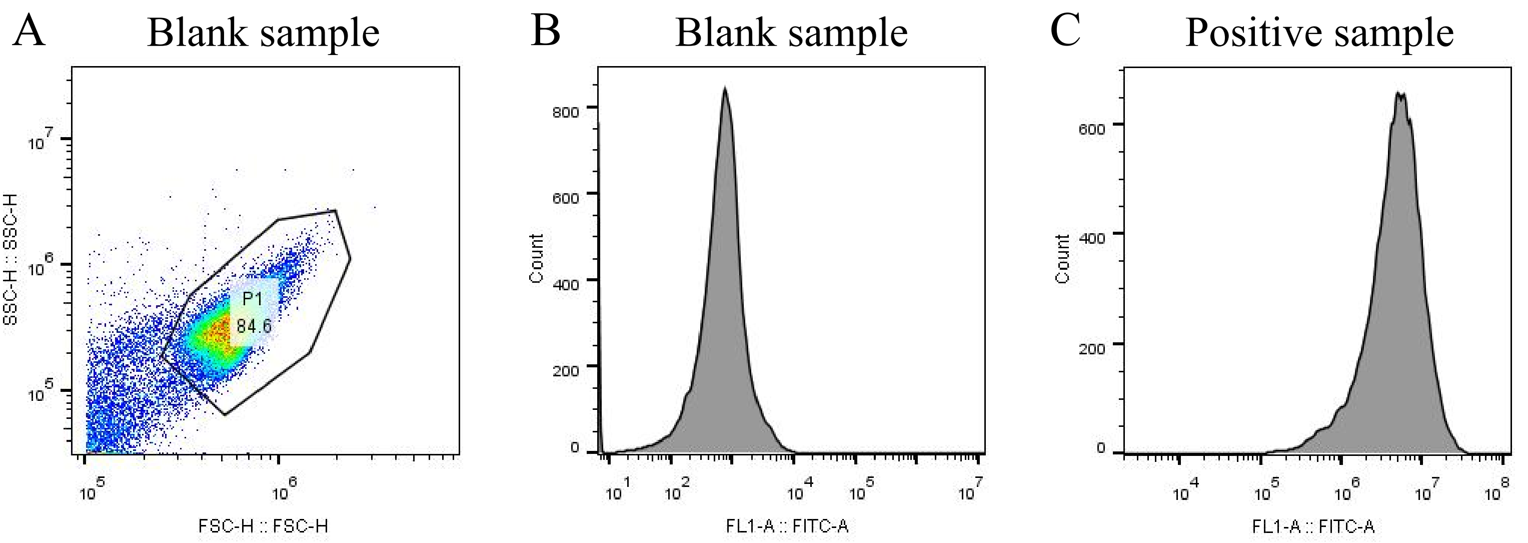

In the analysis of ROS levels, we adopted the following gating strategy:

A: In the blank sample, we used SSA-H and FSC-H to select the aggregated cells.

B: In the blank sample, we used the FITC-A histogram to determine the range of fluorescence values for ROS negative cells.

C: In ROS positive sample, we used the FITC-A histogram to determine the range of fluorescence values of ROS-positive cells, and continued to detect ROS expression levels in each group according to this gating strategy.

Our gating strategy follows the instructions for the Abbkine ANNEXIN V-647 APOPTOSIS Detection KIT. In the experiment, ROS of sample cells in each group had different degrees of activation, and MFI of FITC-A was used for later statistical analysis.

Flow cytometry was used to detect ROS levels

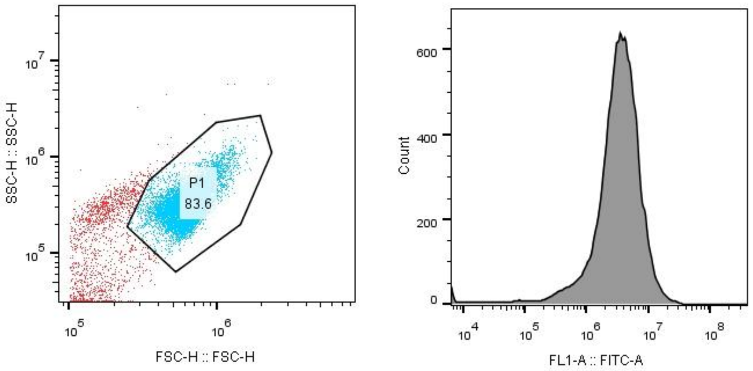

	Sample Name	Subset Name	Count
	2-APB-1.fcs	P1	25082
	2-APB-1.fcs	Ungated	30000

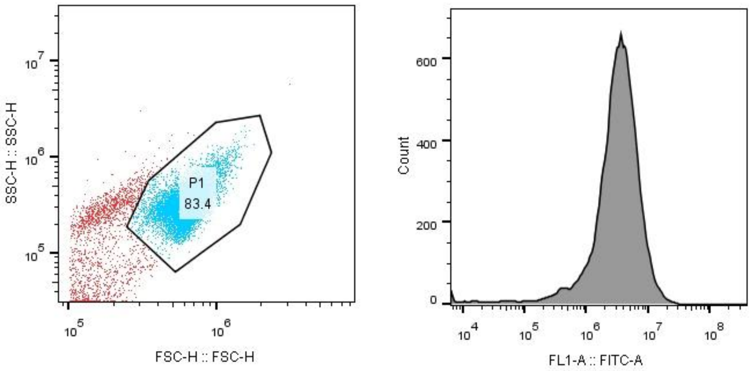

	Sample Name	Subset Name	Count
	2-APB-2.fcs	P1	25020
	2-APB-2.fcs	Ungated	30000

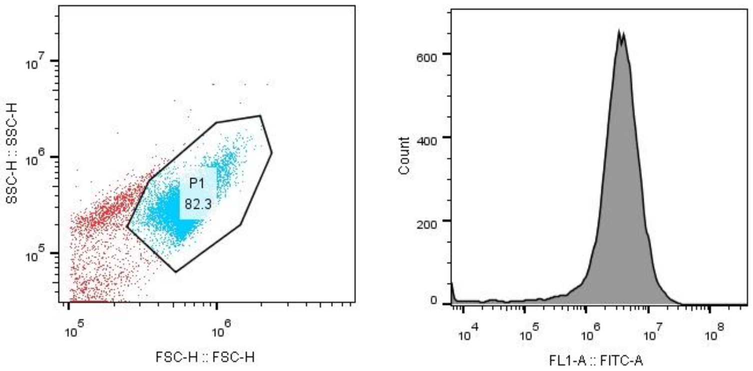

	Sample Name	Subset Name	Count
	2-APB-3.fcs	P1	24679
	2-APB-3.fcs	Ungated	30000

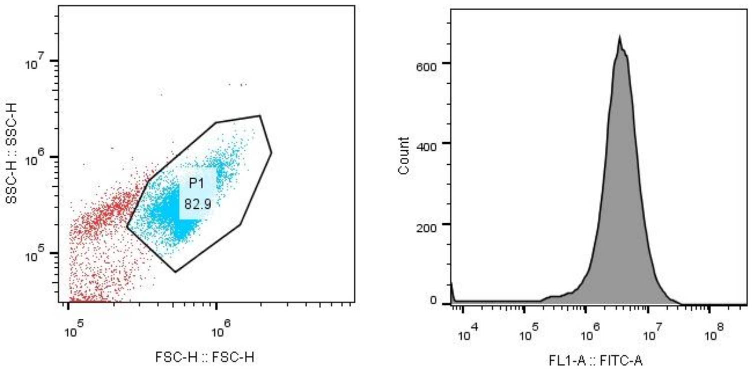

	Sample Name	Subset Name	Count
	2-APB-4.fcs	P1	24862
	2-APB-4.fcs	Ungated	30000

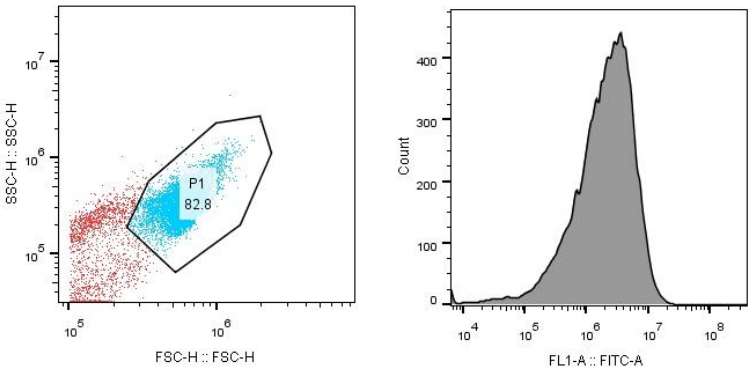

	Sample Name	Subset Name	Count
	CRSJG-1.fcs	P1	24837
	CRSJG-1.fcs	Ungated	30000

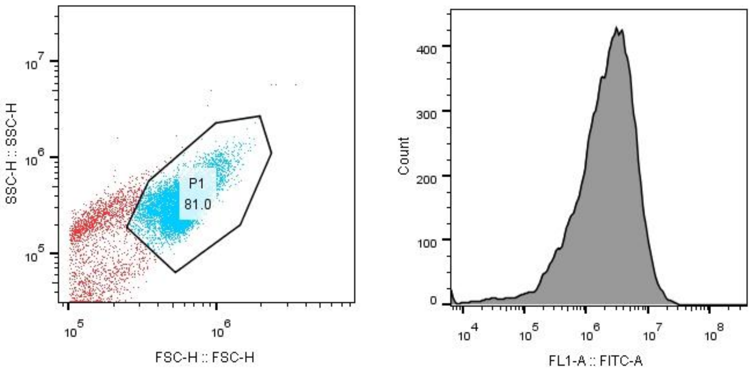

	Sample Name	Subset Name	Count
	CRSJG-2.fcs	P1	24292
	CRSJG-2.fcs	Ungated	30000

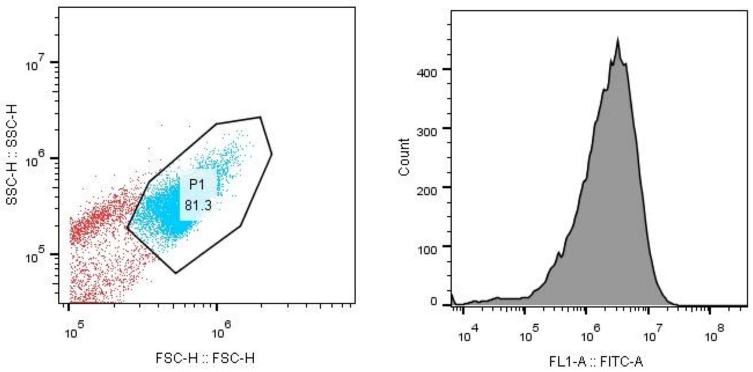

	Sample Name	Subset Name	Count
	CRSJG-3.fcs	P1	24400
	CRSJG-3.fcs	Ungated	30000

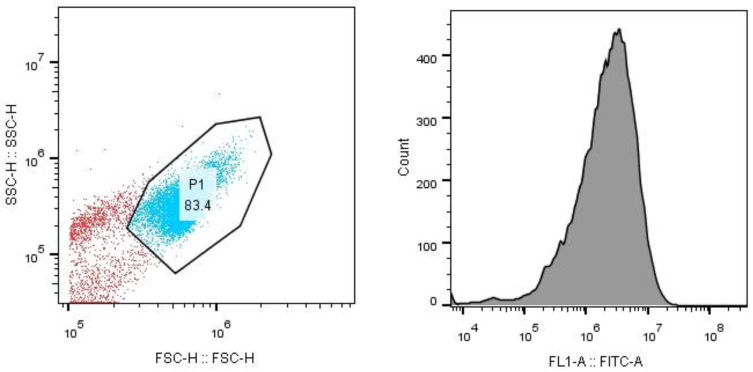

	Sample Name	Subset Name	Count
	CRSJG-4.fcs	P1	25016
	CRSJG-4.fcs	Ungated	30000

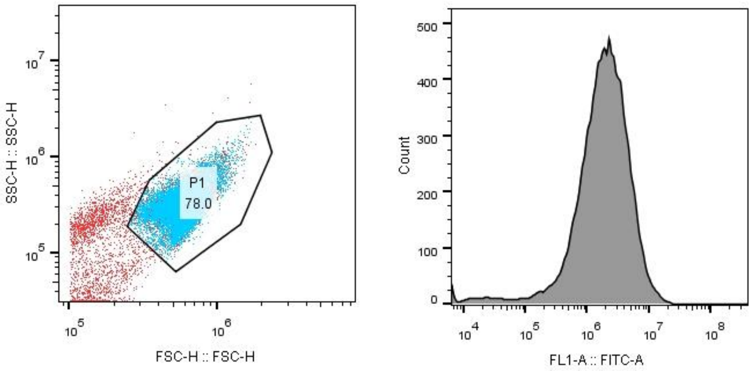

	Sample Name	Subset Name	Count
	Control-1.fcs	P1	23388
	Control-1.fcs	Ungated	30000

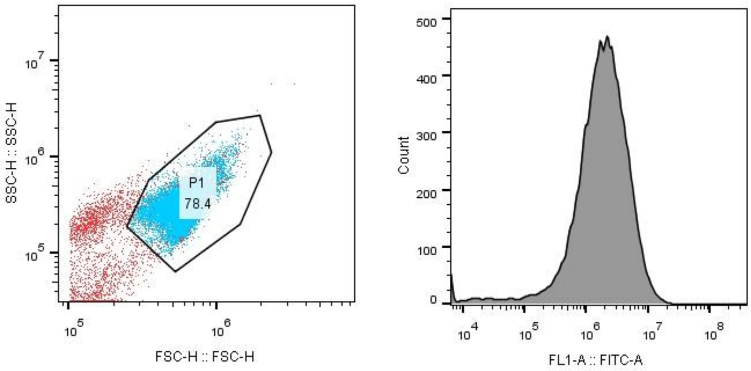

	Sample Name	Subset Name	Count
	Control-2.fcs	P1	23505
	Control-2.fcs	Ungated	30000

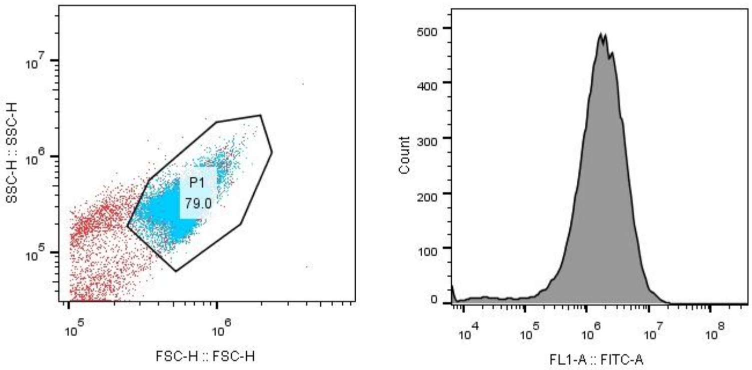

	Sample Name	Subset Name	Count
	Control-3.fcs	P1	23707
	Control-3.fcs	Ungated	30000

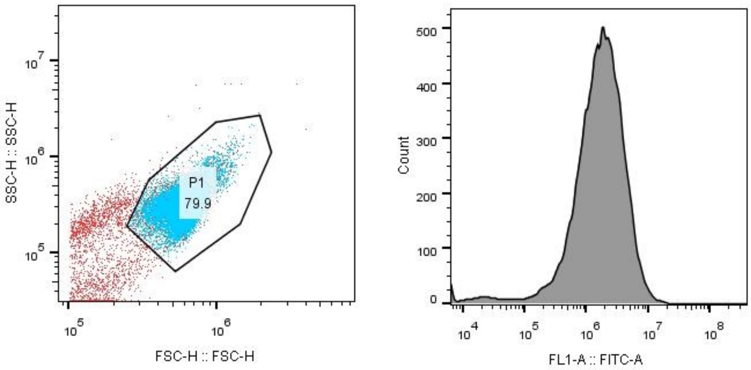

	Sample Name	Subset Name	Count
	Control-4.fcs	P1	23956
	Control-4.fcs	Ungated	30000

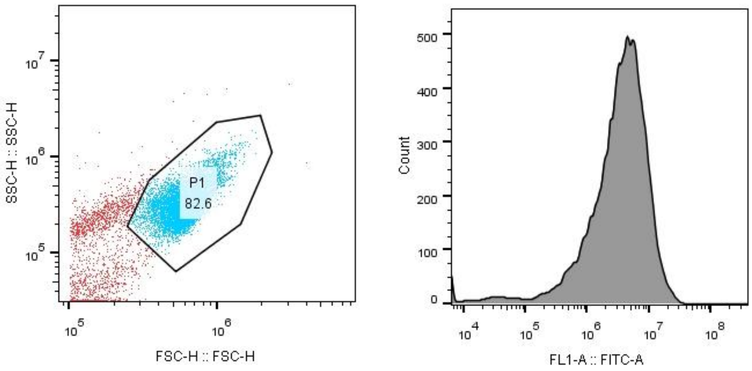

	Sample Name	Subset Name	Count
	MPP+-1.fcs	P1	24765
	MPP+-1.fcs	Ungated	30000

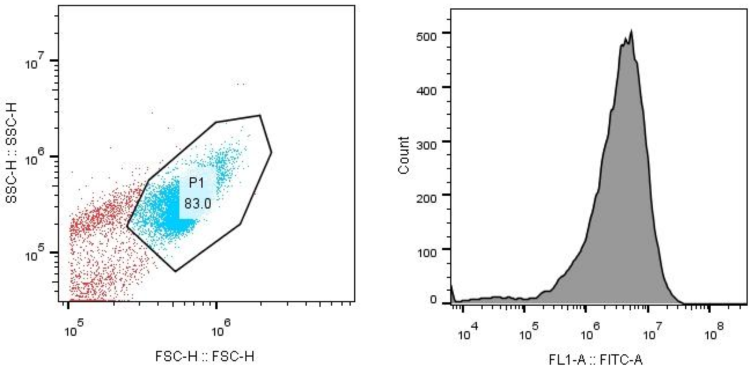

	Sample Name	Subset Name	Count
	MPP+-2.fcs	P1	24902
	MPP+-2.fcs	Ungated	30000

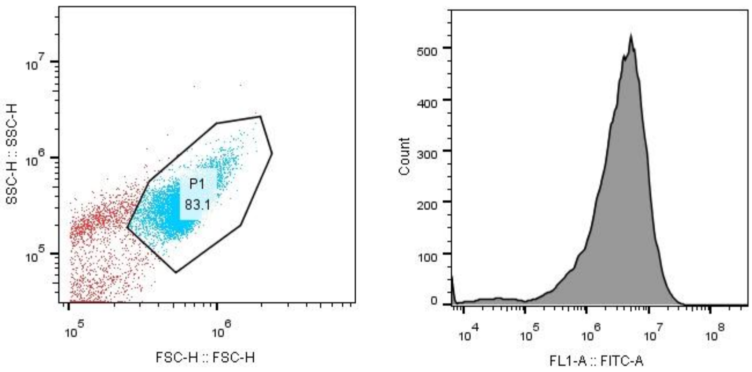

	Sample Name	Subset Name	Count
	MPP+-3.fcs	P1	24931
	MPP+-3.fcs	Ungated	30000

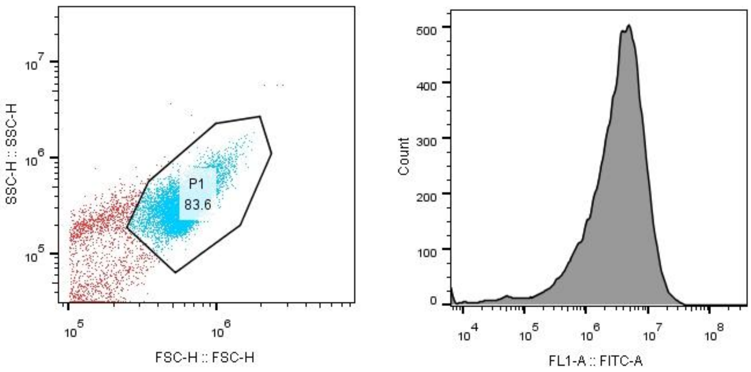

	Sample Name	Subset Name	Count
	MPP+-4.fcs	P1	25093
	MPP+-4.fcs	Ungated	30000

	Ungated<br>*Count*	Ungated<br>*Freq. of Total*	P1<br>*Count*	P1<br>*Freq. of Parent*	P1<br>*Freq. of Total*	P1<br>*Mean FITC-A*
Control-1.fcs	30000	100	23388	78	78	2470000.00
Control-2.fcs	30000	100	23505	78.4	78.4	2430000.00
Control-3.fcs	30000	100	23707	79	79	2200000.00
Control-4.fcs	30000	100	23956	79.9	79.9	2140000.00
MPP+-1.fcs	30000	100	24765	82.6	82.6	4520000.00
MPP+-2.fcs	30000	100	24902	83	83	4510000.00
MPP+-3.fcs	30000	100	24931	83.1	83.1	4460000.00
MPP+-4.fcs	30000	100	25093	83.6	83.6	4340000.00
2-APB-1.fcs	30000	100	25082	83.6	83.6	3980000.00
2-APB-2.fcs	30000	100	25020	83.4	83.4	3860000.00
2-APB-3.fcs	30000	100	24679	82.3	82.3	4080000.00
2-APB-4.fcs	30000	100	24862	82.9	82.9	4070000.00
CRSJG-1.fcs	30000	100	24837	82.8	82.8	2820000.00
CRSJG-2.fcs	30000	100	24292	81	81	3020000.00
CRSJG-3.fcs	30000	100	24400	81.3	81.3	3040000.00
CRSJG-4.fcs	30000	100	25016	83.4	83.4	2990000.00
Mean	**30000**	**100**	**24527**	**81.8**	**81.8**	**3430000.00**
SD	**0**	**0**	**584**	**1.93**	**1.93**	**877000.00**

Gating strategy of MMP analysis

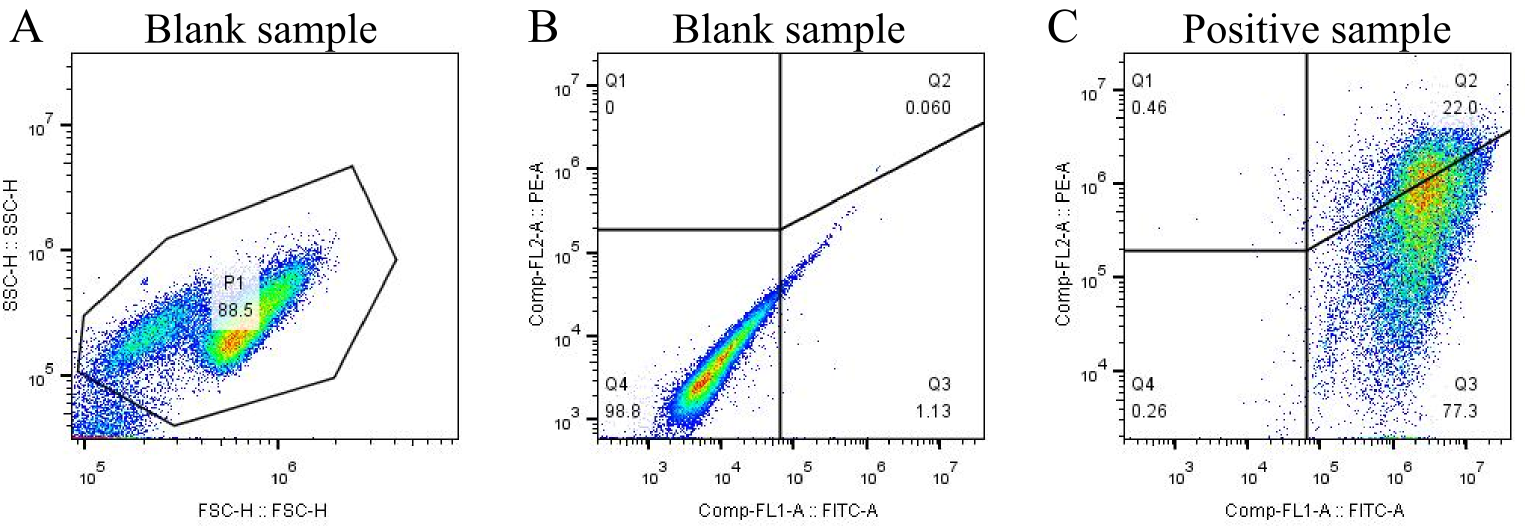

In the analysis of intracellular MMP levels, we adopted the following gating strategy:

A: In the blank sample, we used SSA-H and FSC-H to select the aggregated cells.

B: In the blank sample, we placed all the negative cells in the Q4 region of the blank sample.

C: In the positive sample, our principle is to classify the positive cells as far as possible into the Q3 region. However, since after JC-1 staining, there was a clear grouping of high-MMP cells and low-MMP cells in each group, we adjusted the division of Q2 and Q3 regions according to the actual situation, and divided them at the midpoint of the grouping.

Our gating strategy was carried out in accordance with the ***enhanced mitochondrial membrane potential assay kit with JC-1*** specification, and in consultation with Beyotime's flow analysis technical consultants. In the data analysis, we calculated the proportion of Q2 and Q3 regions , and used Q2/Q3 as the standard to evaluate the MMP levels.

Flow cytometry was used to detect the cyto-Ca^{2+} levels

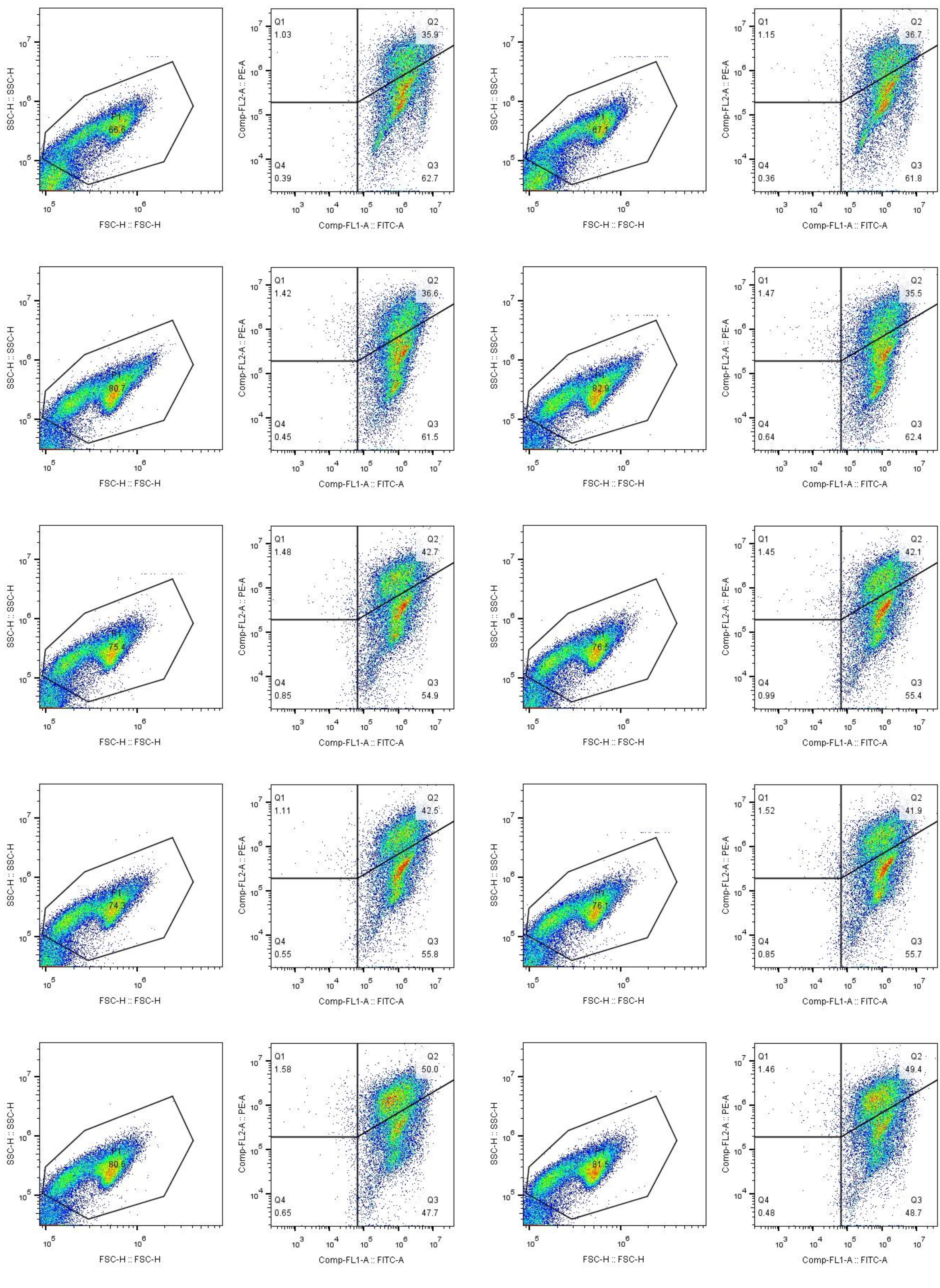

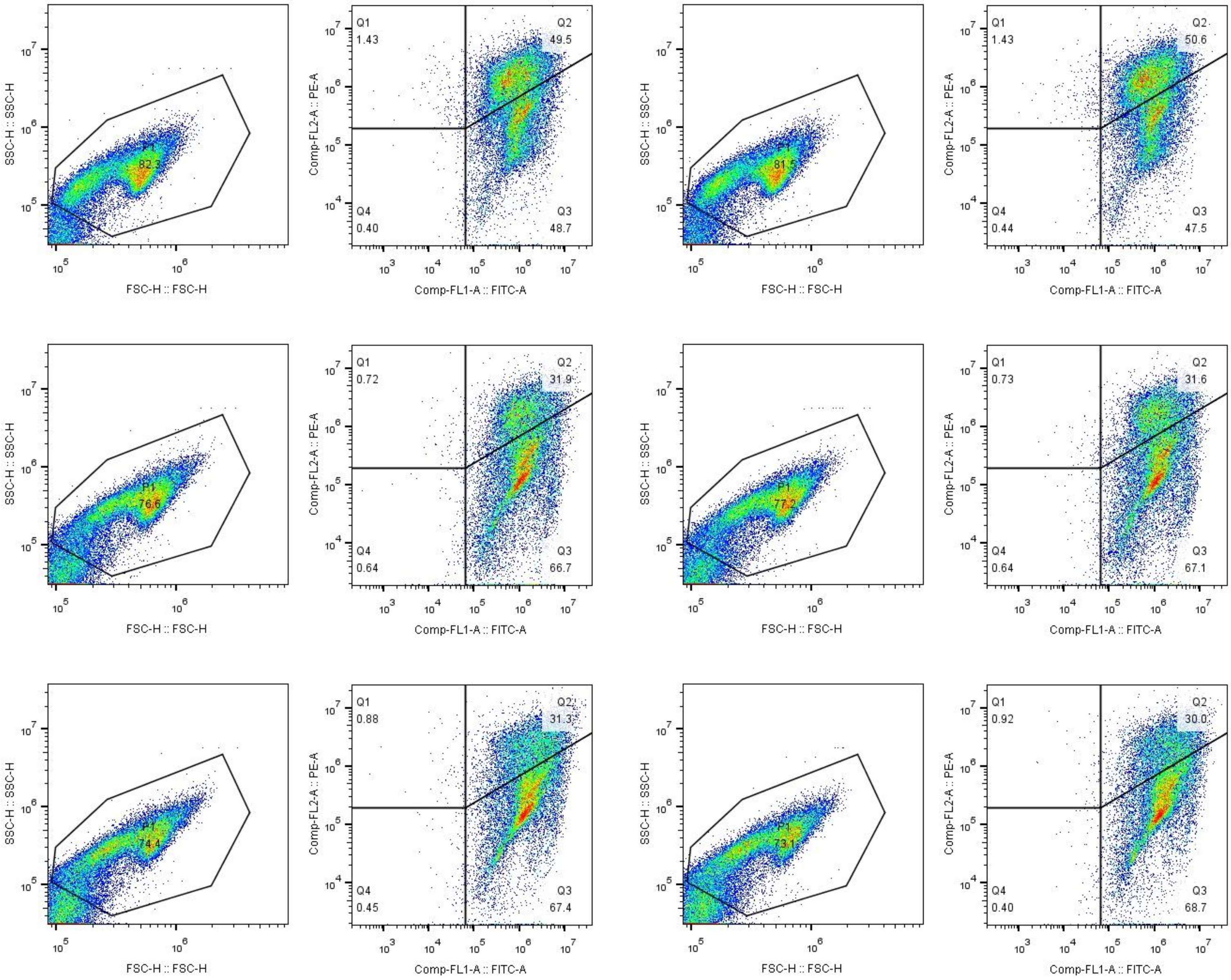
SSC-H :: SSC-H
FSC-H :: FSC-H
P1
82.3
Comp-FL2-A :: PE-A
Comp-FL1-A :: FITC-A
Q1 1.43
Q2 49.5
Q3 48.7
Q4 0.40
P1
81.5
Q1 1.43
Q2 50.6
Q3 47.5
Q4 0.44
P1
76.6
Q1 0.72
Q2 31.9
Q3 66.7
Q4 0.64
P1
77.2
Q1 0.73
Q2 31.6
Q3 67.1
Q4 0.64
P1
74.4
Q1 0.88
Q2 31.3
Q3 67.4
Q4 0.45
P1
73.1
Q1 0.92
Q2 30.0
Q3 68.7
Q4 0.40

	Ungated *Count*	Ungated *Freq. of Total*	Q1: FITC-A-, PE-A+ *Count*	Q1: FITC-A-, PE-A+ *Freq. of Parent*	Q1: FITC-A-, PE-A+ *Freq. of Total*	Q2: FITC-A+, PE-A+ *Count*	Q2: FITC-A+, PE-A+ *Freq. of Parent*	Q2: FITC-A+, PE-A+ *Freq. of Total*	Q3: FITC-A+, PE-A- *Count*	Q3: FITC-A+, PE-A- *Freq. of Parent*	Q3: FITC-A+, PE-A- *Freq. of Total*	Q4: FITC-A-, PE-A- *Count*	Q4: FITC-A-, PE-A- *Freq. of Parent*	Q4: FITC-A-, PE-A- *Freq. of Total*
2-APB-1.fcs	30000	100	206	1.03	0.69	7138	35.7	23.8	12568	62.9	41.9	76	0.38	0.25
2-APB-2.fcs	30000	100	232	1.15	0.77	7268	36.1	24.2	12550	62.4	41.8	73	0.36	0.24
2-APB-3.fcs	30000	100	344	1.42	1.15	8753	36.1	29.2	15039	62.1	50.1	109	0.45	0.36
2-APB-4.fcs	30000	100	366	1.47	1.22	8708	35	29	15663	63	52.2	159	0.64	0.53
CRSJG-1.fcs	30000	100	336	1.48	1.12	9592	42.4	32	12544	55.4	41.8	193	0.85	0.64
CRSJG-2.fcs	30000	100	333	1.45	1.11	9584	41.8	31.9	12819	55.9	42.7	226	0.99	0.75
CRSJG-3.fcs	30000	100	248	1.11	0.83	9385	42.1	31.3	12555	56.3	41.8	122	0.55	0.41
CRSJG-4.fcs	30000	100	346	1.52	1.15	9482	41.5	31.6	12822	56.2	42.7	195	0.85	0.65
Control-1.fcs	30000	100	382	1.58	1.27	11990	49.6	40	11666	48.3	38.9	156	0.65	0.52
Control-2.fcs	30000	100	358	1.46	1.19	11981	49	39.9	12008	49.1	40	118	0.48	0.39
Control-3.fcs	30000	100	352	1.43	1.17	12117	49.1	40.4	12149	49.2	40.5	99	0.4	0.33
Control-4.fcs	30000	100	349	1.43	1.16	12256	50.1	40.9	11765	48.1	39.2	108	0.44	0.36
MPP+-1.fcs	30000	100	166	0.72	0.55	7273	31.6	24.2	15429	67.1	51.4	148	0.64	0.49
MPP+-2.fcs	30000	100	170	0.73	0.57	7226	31.2	24.1	15618	67.5	52.1	148	0.64	0.49
MPP+-3.fcs	30000	100	196	0.88	0.65	6890	30.9	23	15134	67.8	50.4	101	0.45	0.34
MPP+-4.fcs	30000	100	201	0.92	0.67	6490	29.6	21.6	15152	69.1	50.5	88	0.4	0.29
Mean	**30000**	**100**	**287**	**1.24**	**0.95**	**9133**	**39.5**	**30.4**	**13468**	**58.8**	**44.9**	**132**	**0.57**	**0.44**
SD	**0**	**0**	**79.5**	**0.3**	**0.26**	**2040**	**7.22**	**6.81**	**1540**	**7.46**	**5.13**	**45**	**0.19**	**0.15**

Figure 5 related data

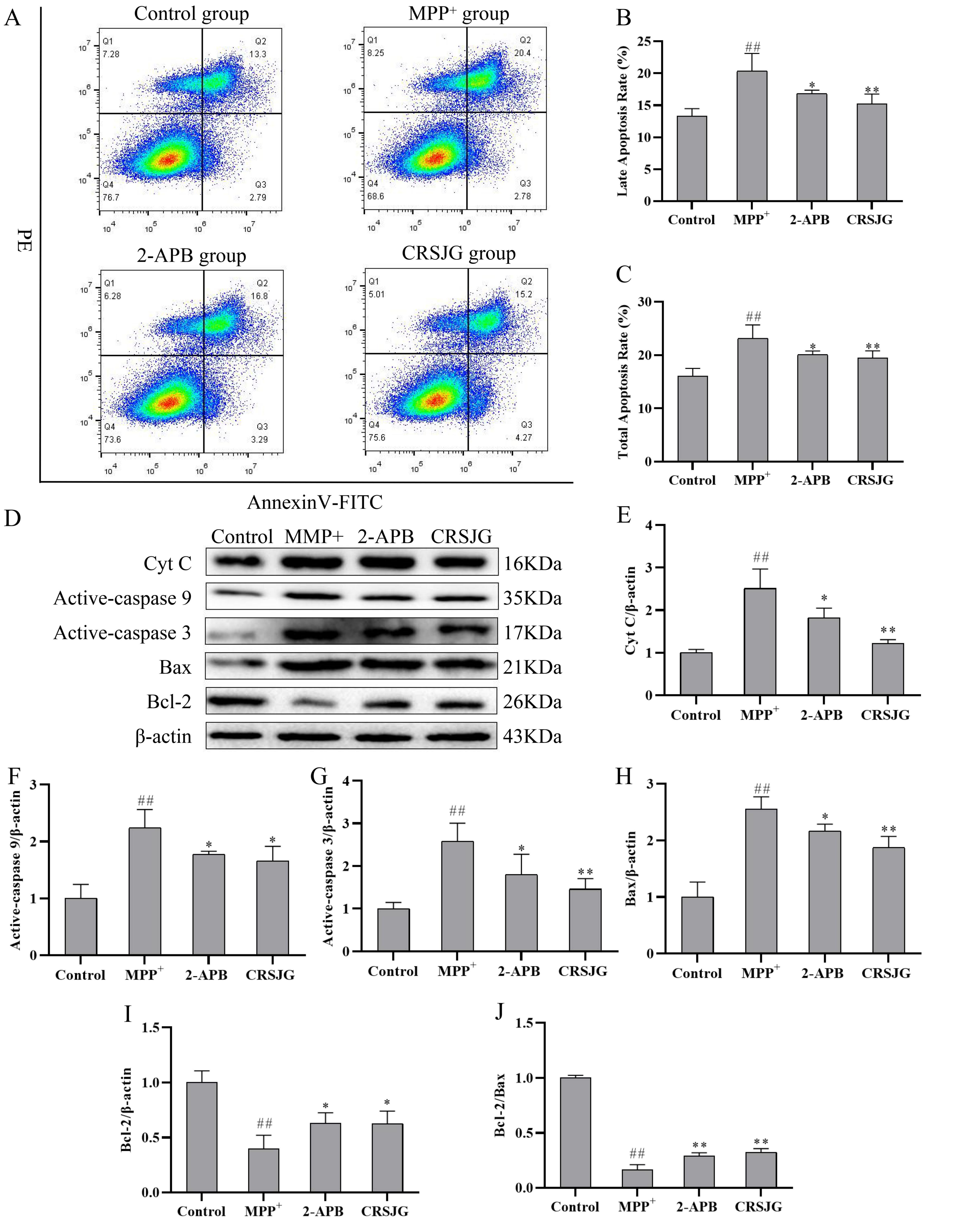

Figure 5. CRSJG can effectively protect neurons from apoptosis induced by MMP+. (A) Flow cytometry was used to detect nerve cell apoptosis; (B) Late apoptosis rate; (C) total cell apoptosis rate; (D) Western blot was used to detect the expression levels of apoptosis-related proteins; (E) Cty C; (F) Active-caspase 9; (G) Active-caspase 3; (H) Bax; (I)Bcl-2; (J)Bcl-2/Bax. Results are showed as mean ± standard deviation (SD), of which (A-C) results are from four independent experiments, and (D-J) results are from three independent experiments. M: # $P < 0.05$, ##$P < 0.01$ vs. control group; * $P < 0.05$, ** $P < 0.01$ vs. MMP+ group.

Gating strategy of apoptosis analysis

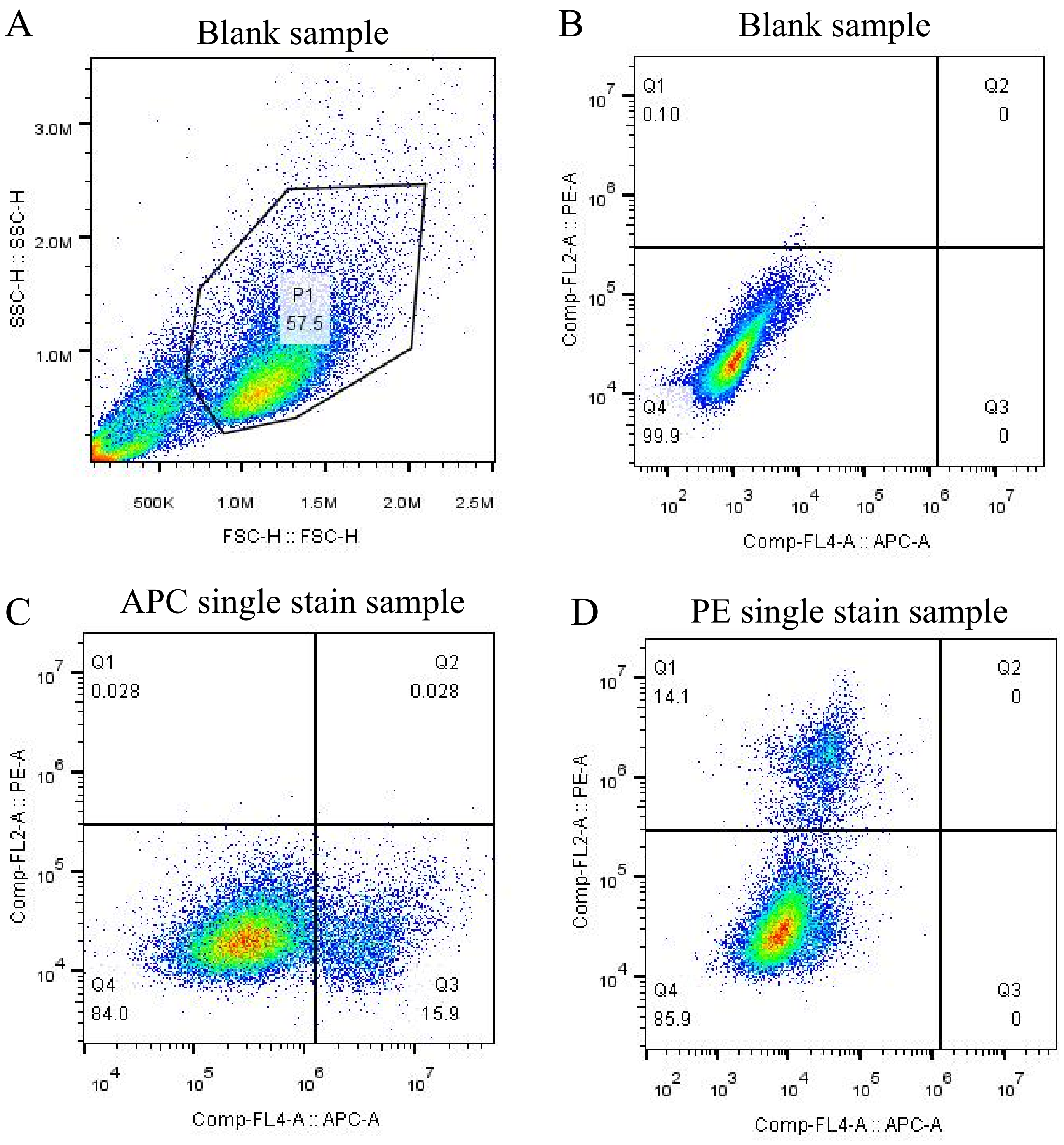

In the analysis of apoptosis cells, we adopted the following gating strategy:

A: In the blank sample, we used SSA-H and FSC-H to select the aggregated cells.

B: In the blank sample, we placed all the negative cells in the Q4 region of the blank sample.

C: In the ANNEXIN-APC monochromic samples, we used ANNEXIN-APC to divide the cells into two distinct clusters that are clearly spaced apart, and we distinguished the APC^- and APC^+ cells at the midpoint of the cluster.

D: In the PI-PE monochromatic sample, we use PI-PE to divide the cells into two distinct groups that are clearly separated from each other, and we distinguish PE^- and PE^+ cells at the midpoint of the group.

Our gating strategy was carried out in accordance with the *ABBkine ANNEXIN V-647 APOPTOSIS Detection KIT* specification, and in consultation with Beyotime's flow analysis technical consultants. In the experiment, we adopted a control strategy for fluorescence Minus One (FMO), which can effectively help us distinguish between negative cells and positive signals from two different dyes. In the results of the experiment, the test cells were clearly divided into three distinct groups, APC^-/PE^- cell group (Q4 region), APC^+/PE^- cell group (Q3 region), and APC^+/PE^+ cell group (Q2 region). In the data analysis, we calculated the proportion of Q2 and Q3 regions.

Flow cytometry was used to detect nerve cell apoptosis

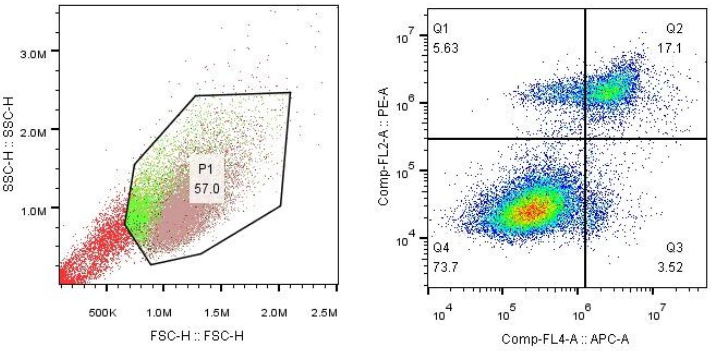

	Sample Name	Subset Name	Count
	2-APB-1.fcs	Q4: APC-A- , PE-A-	16821
	2-APB-1.fcs	Q3: APC-A+ , PE-A-	803
	2-APB-1.fcs	Q2: APC-A+ , PE-A+	3904
	2-APB-1.fcs	Q1: APC-A- , PE-A+	1285
	2-APB-1.fcs	P1	22813
	2-APB-1.fcs	Ungated	40000

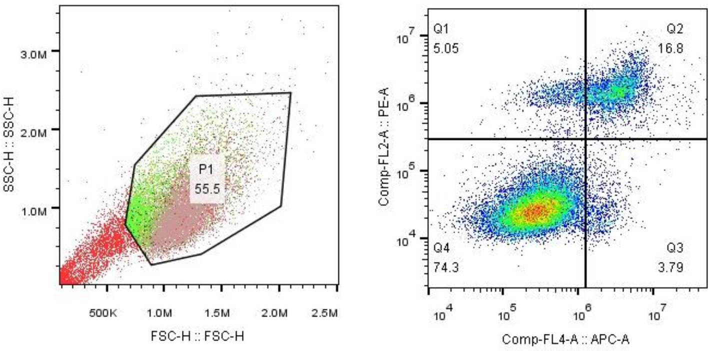

	Sample Name	Subset Name	Count
	2-APB-2.fcs	Q4: APC-A- , PE-A-	16496
	2-APB-2.fcs	Q3: APC-A+ , PE-A-	842
	2-APB-2.fcs	Q2: APC-A+ , PE-A+	3740
	2-APB-2.fcs	Q1: APC-A- , PE-A+	1120
	2-APB-2.fcs	P1	22198
	2-APB-2.fcs	Ungated	40000

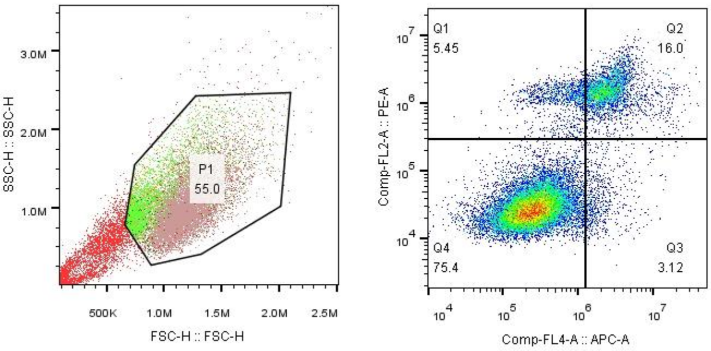

	Sample Name	Subset Name	Count
	2-APB-3.fcs	Q4: APC-A- , PE-A-	16601
	2-APB-3.fcs	Q3: APC-A+ , PE-A-	686
	2-APB-3.fcs	Q2: APC-A+ , PE-A+	3522
	2-APB-3.fcs	Q1: APC-A- , PE-A+	1200
	2-APB-3.fcs	P1	22009
	2-APB-3.fcs	Ungated	40000

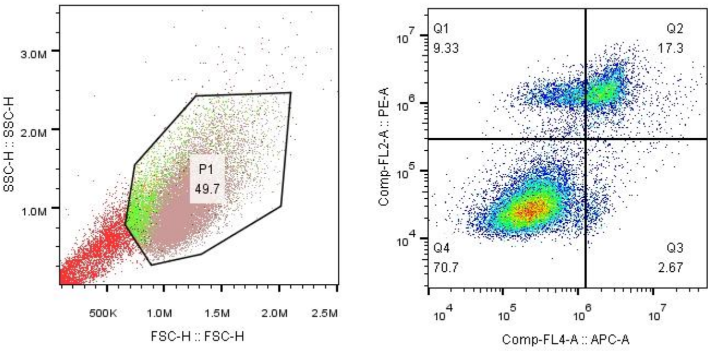

	Sample Name	Subset Name	Count
	2-APB-4.fcs	Q4: APC-A- , PE-A-	14051
	2-APB-4.fcs	Q3: APC-A+ , PE-A-	530
	2-APB-4.fcs	Q2: APC-A+ , PE-A+	3448
	2-APB-4.fcs	Q1: APC-A- , PE-A+	1856
	2-APB-4.fcs	P1	19885
	2-APB-4.fcs	Ungated	40000

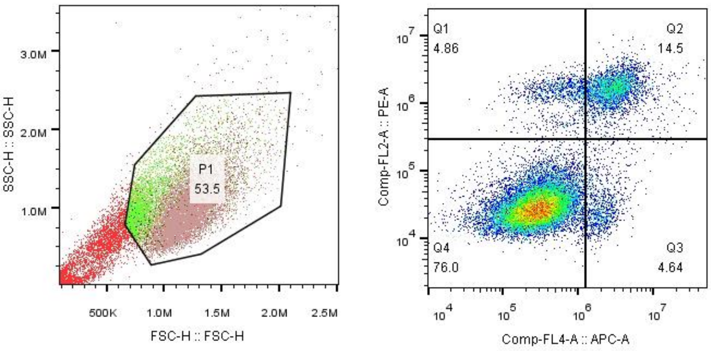

	Sample Name	Subset Name	Count
	CRSJG-1.fcs	Q4: APC-A- , PE-A-	16267
	CRSJG-1.fcs	Q3: APC-A+ , PE-A-	992
	CRSJG-1.fcs	Q2: APC-A+ , PE-A+	3096
	CRSJG-1.fcs	Q1: APC-A- , PE-A+	1040
	CRSJG-1.fcs	P1	21395
	CRSJG-1.fcs	Ungated	40000

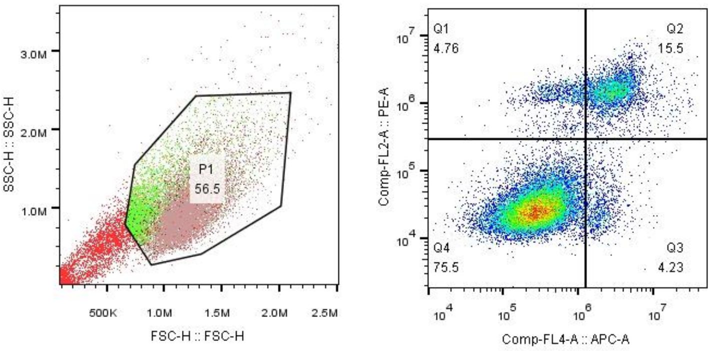

	Sample Name	Subset Name	Count
	CRSJG-2.fcs	Q4: APC-A- , PE-A-	17052
	CRSJG-2.fcs	Q3: APC-A+ , PE-A-	955
	CRSJG-2.fcs	Q2: APC-A+ , PE-A+	3509
	CRSJG-2.fcs	Q1: APC-A- , PE-A+	1076
	CRSJG-2.fcs	P1	22592
	CRSJG-2.fcs	Ungated	40000

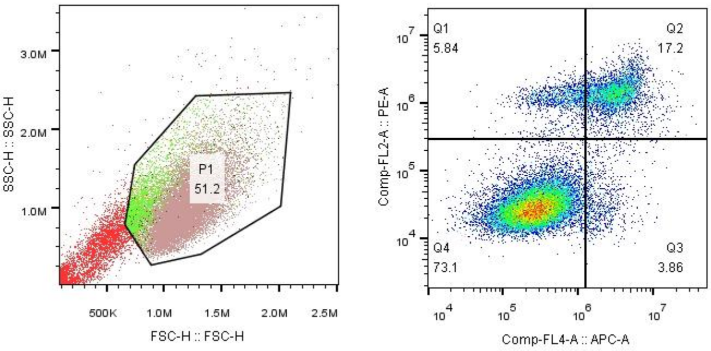

	Sample Name	Subset Name	Count
	CRSJG-3.fcs	Q4: APC-A- , PE-A-	14978
	CRSJG-3.fcs	Q3: APC-A+ , PE-A-	791
	CRSJG-3.fcs	Q2: APC-A+ , PE-A+	3533
	CRSJG-3.fcs	Q1: APC-A- , PE-A+	1197
	CRSJG-3.fcs	P1	20499
	CRSJG-3.fcs	Ungated	40000

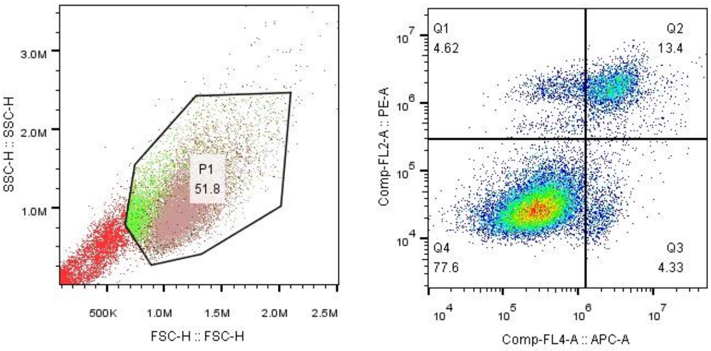

	Sample Name	Subset Name	Count
	CRSJG-4.fcs	Q4: APC-A- , PE-A-	16093
	CRSJG-4.fcs	Q3: APC-A+ , PE-A-	897
	CRSJG-4.fcs	Q2: APC-A+ , PE-A+	2783
	CRSJG-4.fcs	Q1: APC-A- , PE-A+	957
	CRSJG-4.fcs	P1	20730
	CRSJG-4.fcs	Ungated	40000

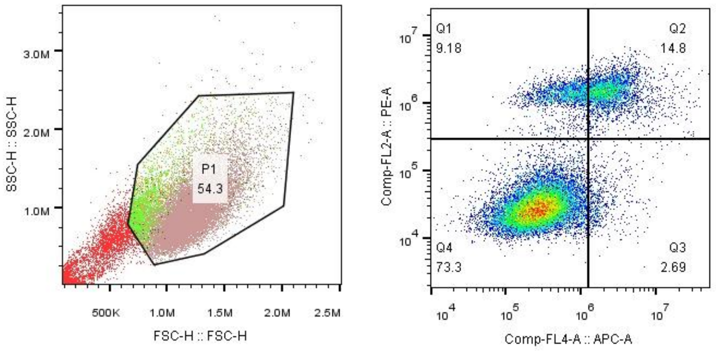

	Sample Name	Subset Name	Count
	Control-1.fcs	Q4: APC-A- , PE-A-	15926
	Control-1.fcs	Q3: APC-A+ , PE-A-	584
	Control-1.fcs	Q2: APC-A+ , PE-A+	3217
	Control-1.fcs	Q1: APC-A- , PE-A+	1993
	Control-1.fcs	P1	21720
	Control-1.fcs	Ungated	40000

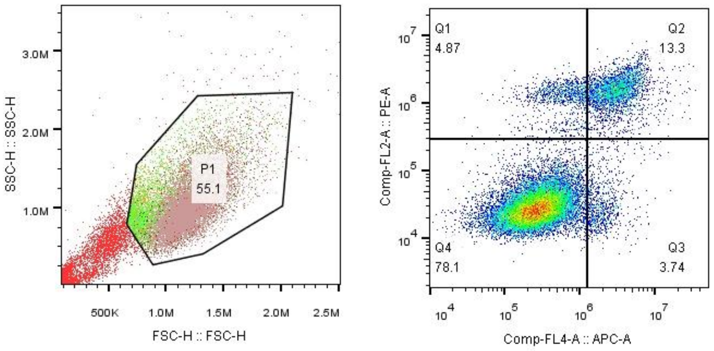

	Sample Name	Subset Name	Count
	Control-2.fcs	Q4: APC-A- , PE-A-	17191
	Control-2.fcs	Q3: APC-A+ , PE-A-	824
	Control-2.fcs	Q2: APC-A+ , PE-A+	2934
	Control-2.fcs	Q1: APC-A- , PE-A+	1073
	Control-2.fcs	P1	22022
	Control-2.fcs	Ungated	40000

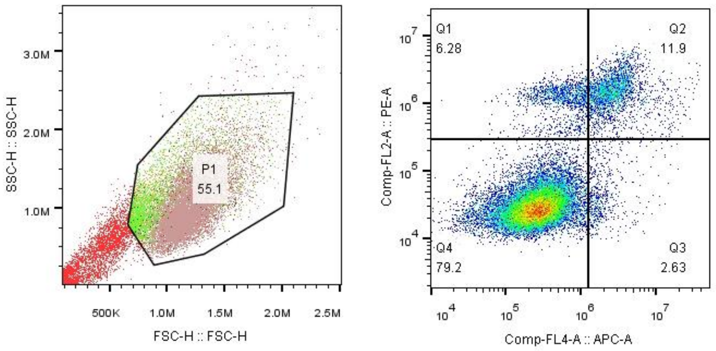

	Sample Name	Subset Name	Count
	Control-3.fcs	Q4: APC-A- , PE-A-	17467
	Control-3.fcs	Q3: APC-A+ , PE-A-	581
	Control-3.fcs	Q2: APC-A+ , PE-A+	2618
	Control-3.fcs	Q1: APC-A- , PE-A+	1384
	Control-3.fcs	P1	22050
	Control-3.fcs	Ungated	40000

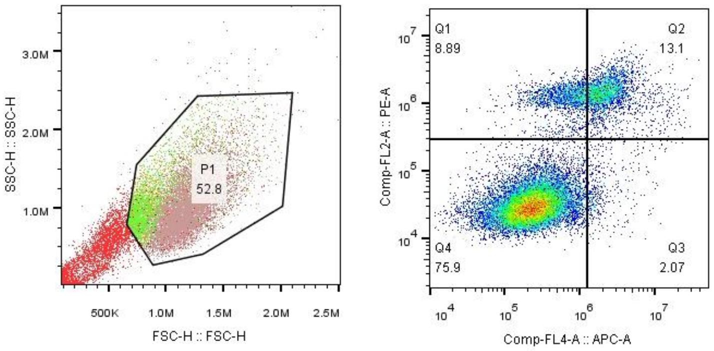

	Sample Name	Subset Name	Count
	Control-4.fcs	Q4: APC-A- , PE-A-	16022
	Control-4.fcs	Q3: APC-A+ , PE-A-	437
	Control-4.fcs	Q2: APC-A+ , PE-A+	2767
	Control-4.fcs	Q1: APC-A- , PE-A+	1876
	Control-4.fcs	P1	21102
	Control-4.fcs	Ungated	40000

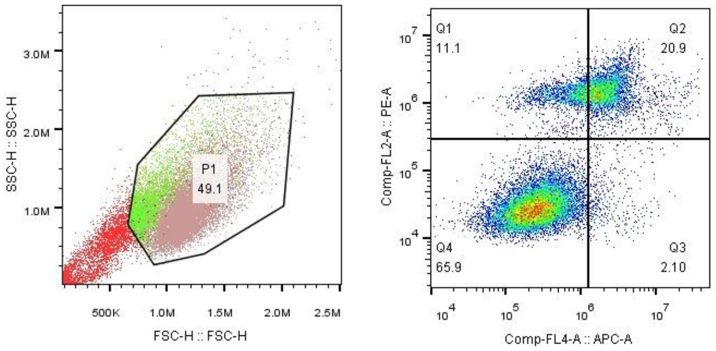

	Sample Name	Subset Name	Count
	MMP+-1.fcs	Q4: APC-A- , PE-A-	12945
	MMP+-1.fcs	Q3: APC-A+ , PE-A-	412
	MMP+-1.fcs	Q2: APC-A+ , PE-A+	4098
	MMP+-1.fcs	Q1: APC-A- , PE-A+	2179
	MMP+-1.fcs	P1	19634
	MMP+-1.fcs	Ungated	40000

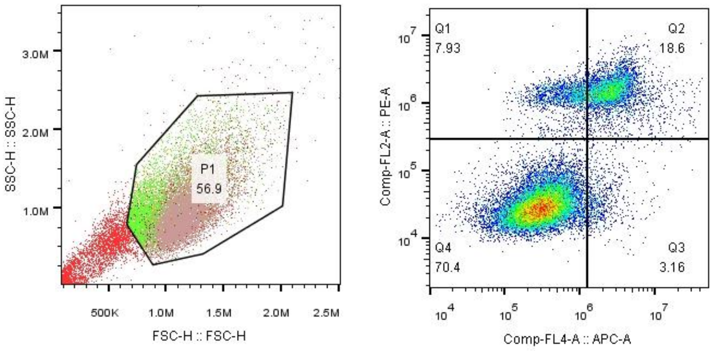

	Sample Name	Subset Name	Count
	MMP+-2.fcs	Q4: APC-A- , PE-A-	16018
	MMP+-2.fcs	Q3: APC-A+ , PE-A-	719
	MMP+-2.fcs	Q2: APC-A+ , PE-A+	4225
	MMP+-2.fcs	Q1: APC-A- , PE-A+	1805
	MMP+-2.fcs	P1	22767
	MMP+-2.fcs	Ungated	40000

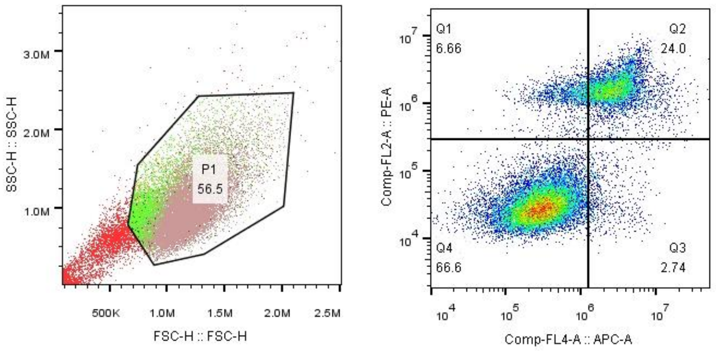

	Sample Name	Subset Name	Count
	MMP+-3.fcs	Q4: APC-A- , PE-A-	15045
	MMP+-3.fcs	Q3: APC-A+ , PE-A-	619
	MMP+-3.fcs	Q2: APC-A+ , PE-A+	5414
	MMP+-3.fcs	Q1: APC-A- , PE-A+	1503
	MMP+-3.fcs	P1	22581
	MMP+-3.fcs	Ungated	40000

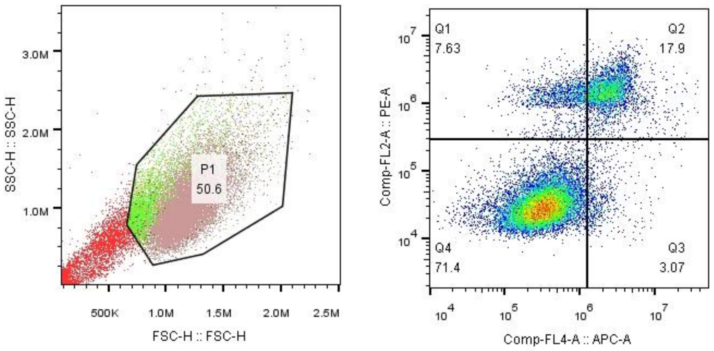

	Sample Name	Subset Name	Count
	MMP+-4.fcs	Q4: APC-A- , PE-A-	14464
	MMP+-4.fcs	Q3: APC-A+ , PE-A-	621
	MMP+-4.fcs	Q2: APC-A+ , PE-A+	3630
	MMP+-4.fcs	Q1: APC-A- , PE-A+	1545
	MMP+-4.fcs	P1	20260
	MMP+-4.fcs	Ungated	40000

	Ungated *Count*	Ungated *Freq. of Total*	P1 *Count*	P1 *Freq. of Parent*	P1 *Freq. of Total*	*P1* Q1: APC-A-, PE-A+ *Count*	*P1* Q1: APC-A-, PE-A+ *Freq. of Parent*	*P1* Q1: APC-A-, PE-A+ *Freq. of Total*	*P1* Q2: APC-A+, PE-A+ *Count*	*P1* Q2: APC-A+, PE-A+ *Freq. of Parent*	*P1* Q2: APC-A+, PE-A+ *Freq. of Total*	*P1* Q3: APC-A+, PE-A- *Count*	*P1* Q3: APC-A+, PE-A- *Freq. of Parent*	*P1* Q3: APC-A+, PE-A- *Freq. of Total*	*P1* Q4: APC-A-, PE-A- *Count*	*P1* Q4: APC-A-, PE-A- *Freq. of Parent*	*P1* Q4: APC-A-, PE-A- *Freq. of Total*
Control-1.fcs	40000	100	21720	54.3	54.3	1993	9.18	4.98	3217	14.8	8.04	584	2.69	1.46	15926	73.3	39.8
Control-2.fcs	40000	100	22022	55.1	55.1	1073	4.87	2.68	2934	13.3	7.34	824	3.74	2.06	17191	78.1	43
Control-3.fcs	40000	100	22050	55.1	55.1	1384	6.28	3.46	2618	11.9	6.54	581	2.63	1.45	17467	79.2	43.7
Control-4.fcs	40000	100	21102	52.8	52.8	1876	8.89	4.69	2767	13.1	6.92	437	2.07	1.09	16022	75.9	40.1
MMP+-1.fcs	40000	100	19634	49.1	49.1	2179	11.1	5.45	4098	20.9	10.2	412	2.1	1.03	12945	65.9	32.4
MMP+-2.fcs	40000	100	22767	56.9	56.9	1805	7.93	4.51	4225	18.6	10.6	719	3.16	1.8	16018	70.4	40
MMP+-3.fcs	40000	100	22581	56.5	56.5	1503	6.66	3.76	5414	24	13.5	619	2.74	1.55	15045	66.6	37.6
MMP+-4.fcs	40000	100	20260	50.6	50.6	1545	7.63	3.86	3630	17.9	9.08	621	3.07	1.55	14464	71.4	36.2
2-APB-1.fcs	40000	100	22813	57	57	1285	5.63	3.21	3904	17.1	9.76	803	3.52	2.01	16821	73.7	42.1
2-APB-2.fcs	40000	100	22198	55.5	55.5	1120	5.05	2.8	3740	16.8	9.35	842	3.79	2.1	16496	74.3	41.2
2-APB-3.fcs	40000	100	22009	55	55	1200	5.45	3	3522	16	8.8	686	3.12	1.71	16601	75.4	41.5
2-APB-4.fcs	40000	100	19885	49.7	49.7	1856	9.33	4.64	3448	17.3	8.62	530	2.67	1.32	14051	70.7	35.1
CRSJG-1.fcs	40000	100	21395	53.5	53.5	1040	4.86	2.6	3096	14.5	7.74	992	4.64	2.48	16267	76	40.7
CRSJG-2.fcs	40000	100	22592	56.5	56.5	1076	4.76	2.69	3509	15.5	8.77	955	4.23	2.39	17052	75.5	42.6
CRSJG-3.fcs	40000	100	20499	51.2	51.2	1197	5.84	2.99	3533	17.2	8.83	791	3.86	1.98	14978	73.1	37.4
CRSJG-4.fcs	40000	100	20730	51.8	51.8	957	4.62	2.39	2783	13.4	6.96	897	4.33	2.24	16093	77.6	40.2
Mean	**40000**	**100**	**21516**	**53.8**	**53.8**	**1443**	**6.75**	**3.61**	**3527**	**16.4**	**8.82**	**706**	**3.27**	**1.76**	**15840**	**73.6**	**39.6**
SD	**0**	**0**	**1047**	**2.63**	**2.63**	**389**	**2.01**	**0.97**	**691**	**3.1**	**1.72**	**176**	**0.78**	**0.44**	**1240**	**3.81**	**3.1**

Western blot was used to detect the expression levels of apoptosis-related proteins

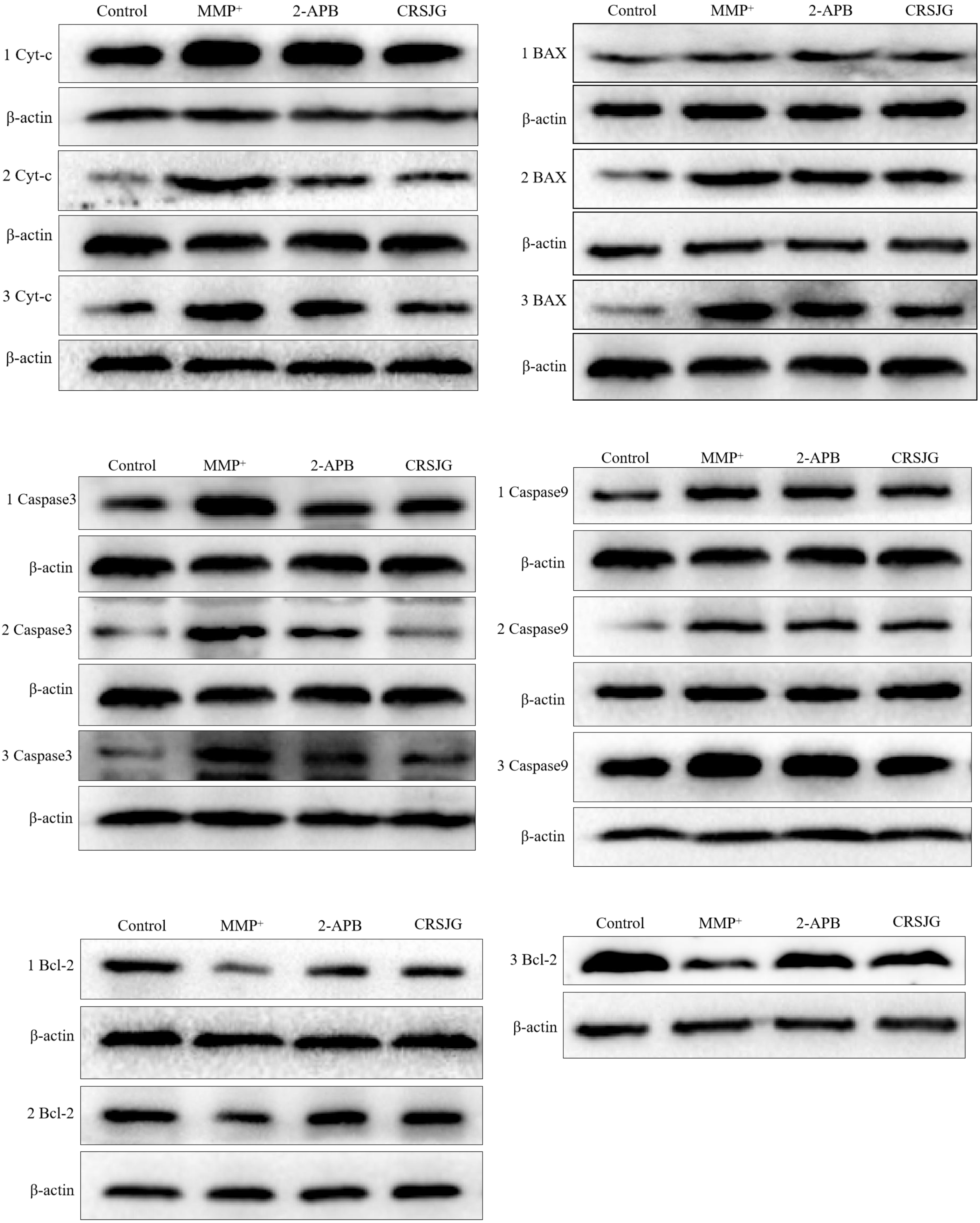

	Target protein（Gray value）			β-actin（Gray value）			ratio		
	sample 1	sample 2	sample 3	sample 1	sample 2	sample 3	sample 1	sample 2	sample 3
Cyt c	4092725	2756936	2541462	7538710	5150400	4867065	0.542894607	0.535285803	0.493449441
	8587600	5862624	6866607	8292063	4349960	5343520	1.03564095	1.347742048	1.57854486
	8015000	3948080	5069592	7623168	4804454	5072315	1.051400153	0.821754147	0.999463164
	5097680	3257415	2971980	7852935	4953528	5005455	0.649143282	0.657594951	0.599972383
Bax	1752978	3802638	1988040	2730566	6023710	5150400	0.641983384	0.631278398	0.385997204
	4104240	10199610	6124720	3157308	6646252	4349960	1.299917525	1.534640877	1.407994556
	3724920	8296214	5401225	3081312	6589336	4804454	1.208874661	1.259036419	1.124212033
	3064950	7201643	5543718	3361824	6687695	4953528	0.911692581	1.076849797	1.119145385
Bcl-2	6500772	7090710	7867600	4867065	6501088	6023710	1.335665745	1.090695896	1.306105374
	2442325	4749657	2416655	5343520	7172235	6646252	0.457062947	0.662228301	0.363611702
	4095503	6187512	4304765	5012315	7019516	6589336	0.817088112	0.88147274	0.653292684
	3964213	6680296	4221566	5005455	7314109	6687695	0.791978551	0.913343785	0.631243799
Active-caspase 3	3045910	3798240	3502680	5150400	5150400	6149880	0.591392902	0.737465051	0.569552577
	6725950	8417556	9947664	4349960	4349960	6940720	1.54620962	1.935088139	1.433232287
	4180295	5226372	8731200	4804454	4804454	5962732	0.870087423	1.087818095	1.464295226
	3713045	4917564	7434984	4953528	4953528	7198503	0.749575858	0.99273972	1.032851414
Active-caspase 9	5467506	1901133	2356868	5150400	2730566	2071994	1.061569199	0.696241365	1.13748785
	9100134	5984388	5232294	4349960	3157308	2090370	2.092004064	1.895408367	2.503046829
	8491577	5261984	3802491	4804454	3081312	2272816	1.767438506	1.707708924	1.673030725
	7634836	4648611	3833600	4953528	3361824	2046646	1.54129259	1.382764535	1.873113377

Figure 6 related data

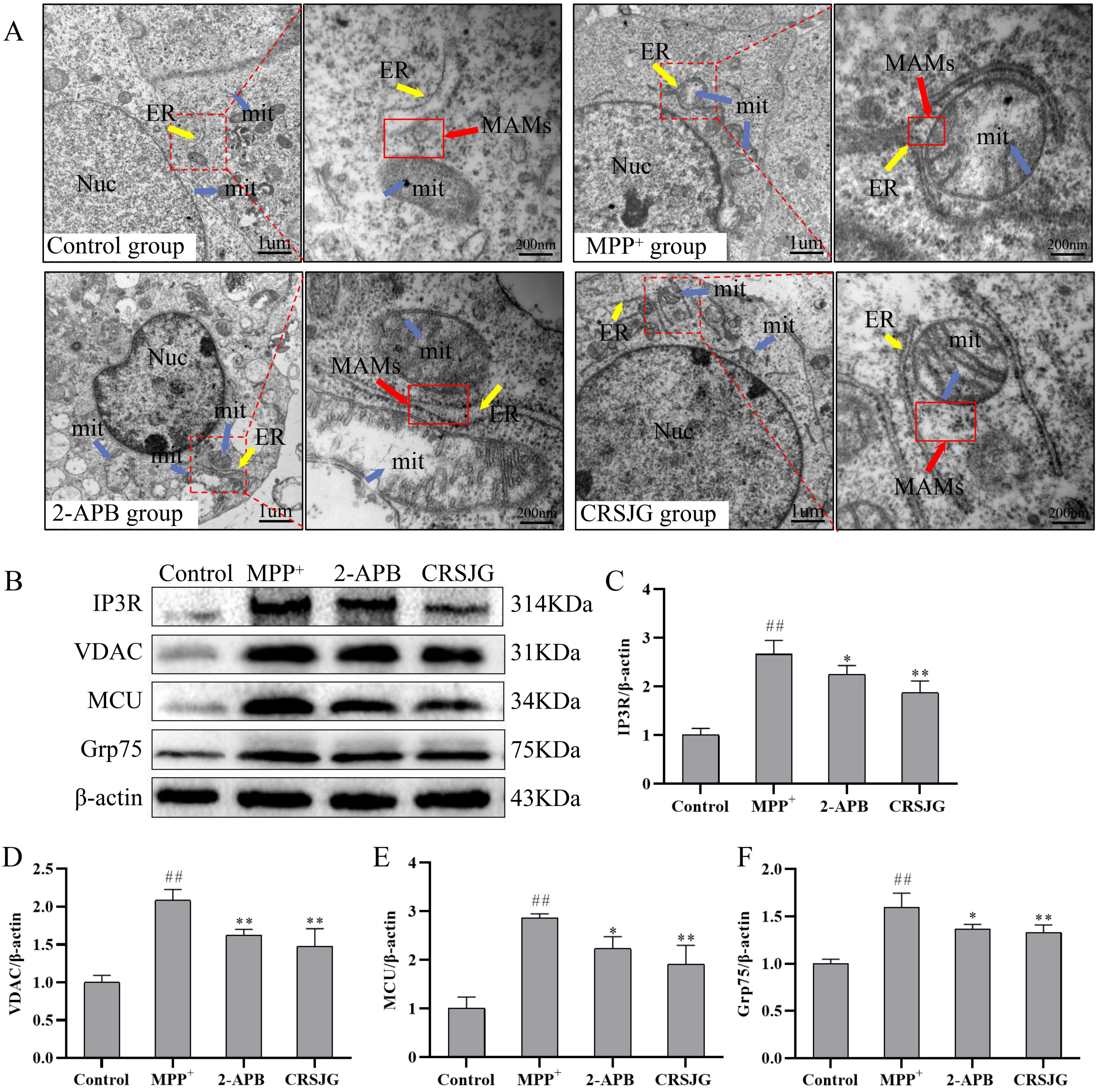

Figure 6. CRSJG protects the structure and function of MAMs in MMP$^+$-induced nerve cells. (A) The microscopic structure of MAMS was observed by transmission electron microscope, magnification (×20000), magnification of red rectangle box (×100000); (B) Western blot was used to detect the expression of major proteins in the Ca^{2+} transport complex; (C) IP3R; (D) VDAC; (E) MCU; (G) Grp75. results are from three independent experiments. M: # $P < 0.05$, ##$P < 0.01$ vs. control group; * $P < 0.05$, ** $P < 0.01$ vs. MMP$^+$ group.

The microscopic structure of MAMS was observed by transmission electron microscope, magnification (×20000), magnification of red rectangle box (×100000)

Control

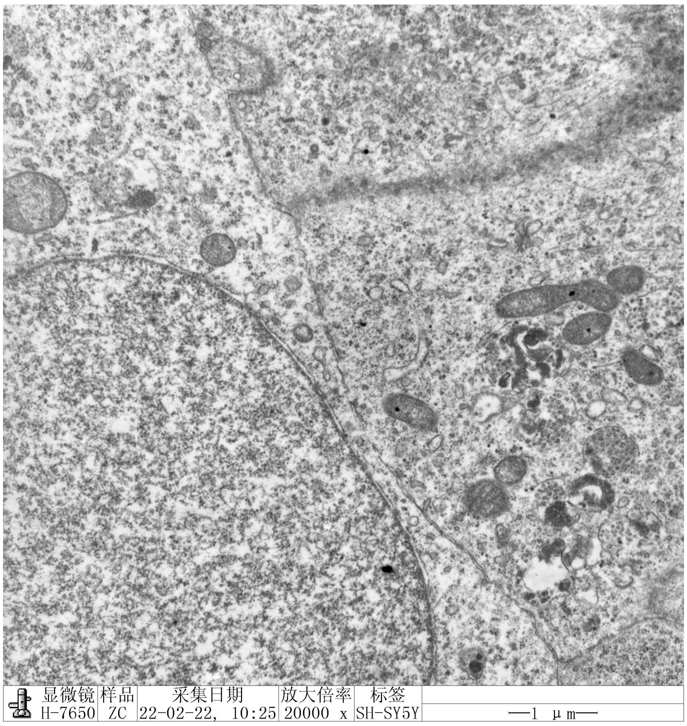

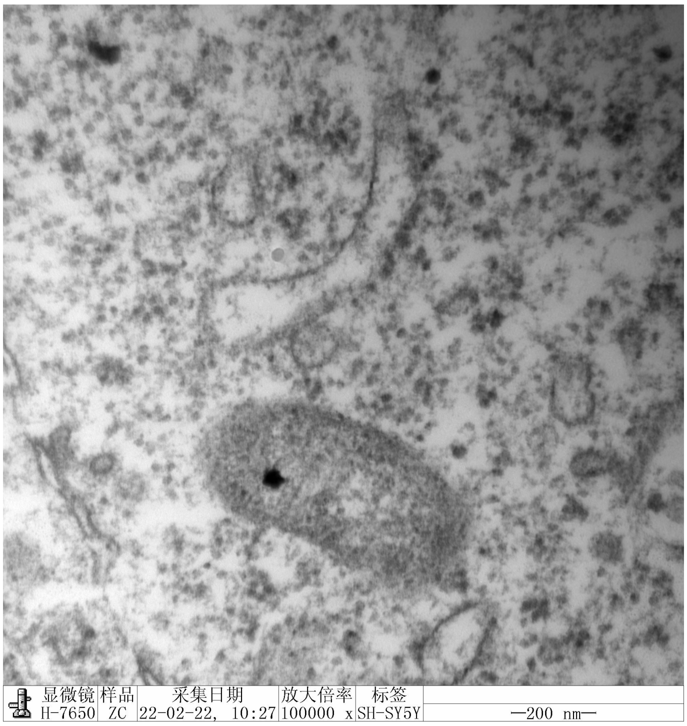

MPP^+

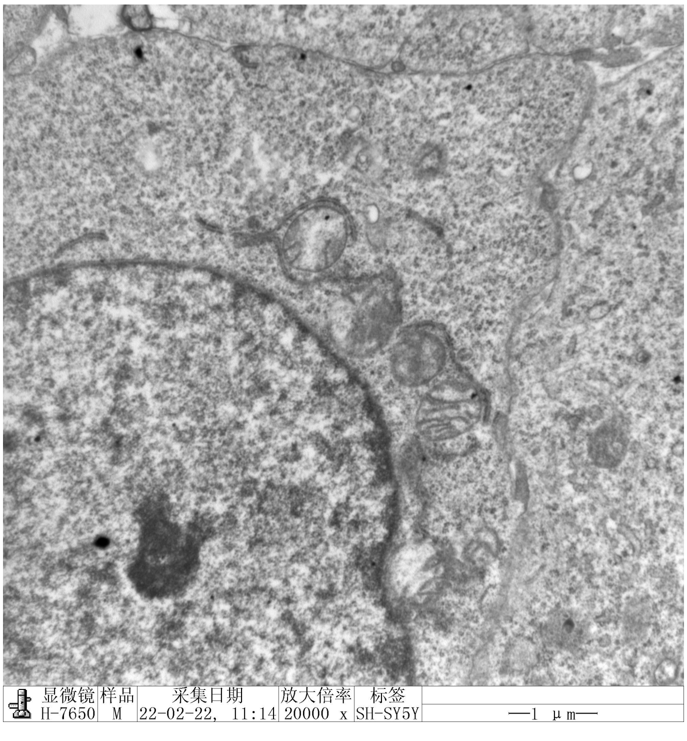

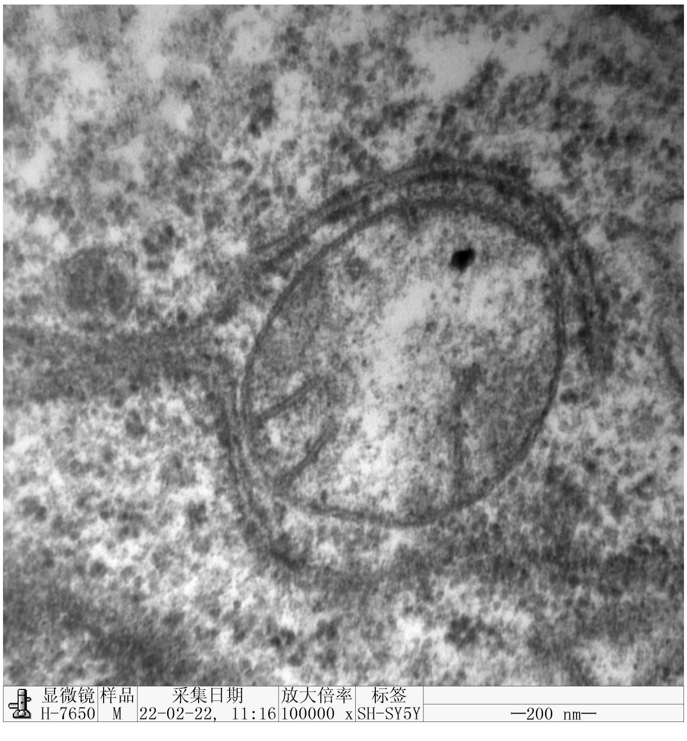

2-APB

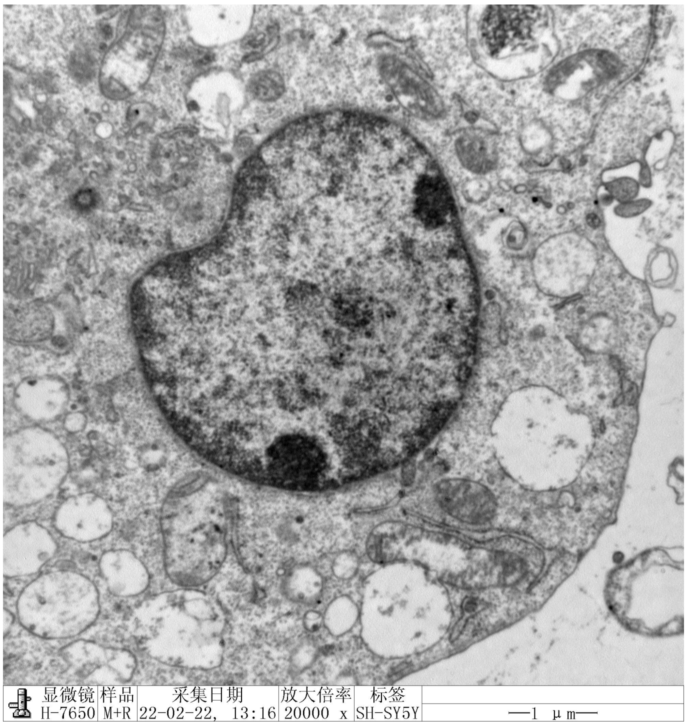
显微镜 样品 采集日期 放大倍率 标签
H-7650 M+R 22-02-22, 13:16 20000 x SH-SY5Y
1 μm

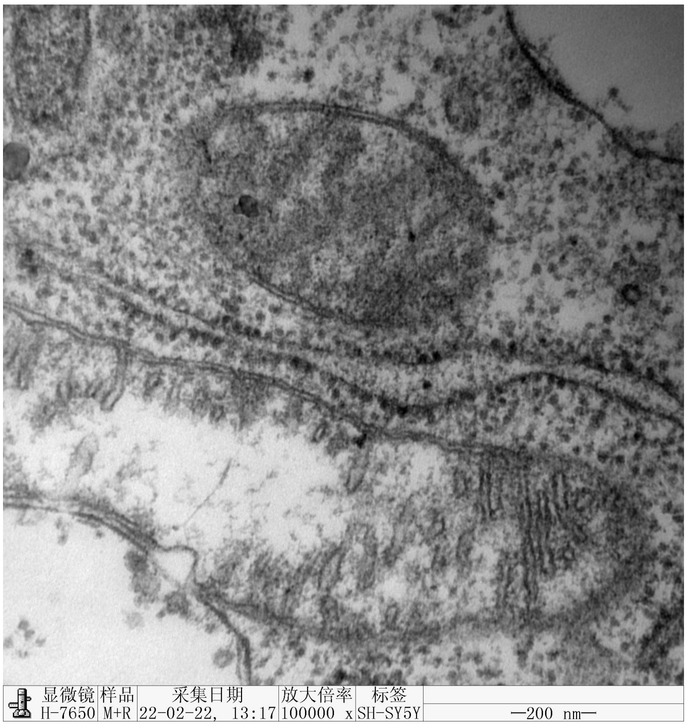
显微镜 样品 采集日期 放大倍率 标签
H-7650 M+R 22-02-22, 13:17 100000 x SH-SY5Y
200 nm

CRSJG

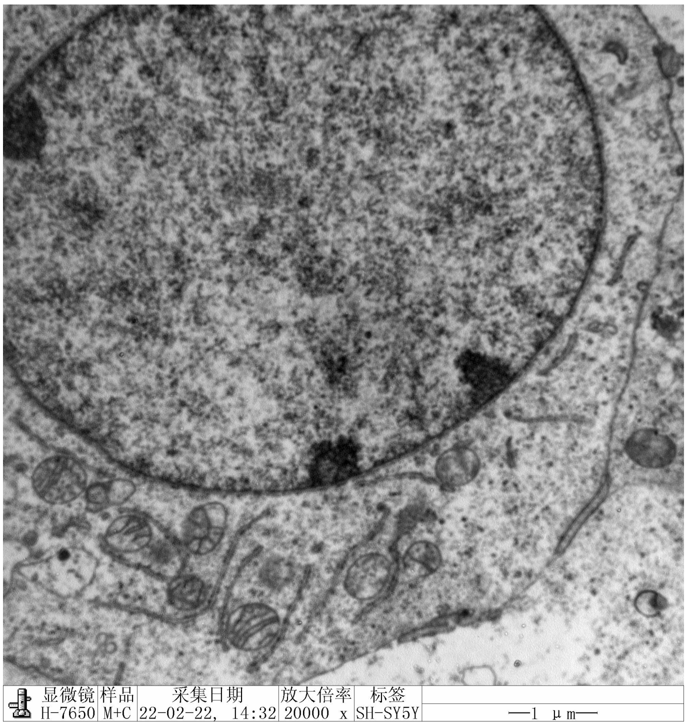
显微镜 样品 采集日期 放大倍率 标签
H-7650 M+C 22-02-22, 14:32 20000 x SH-SY5Y
1 μm

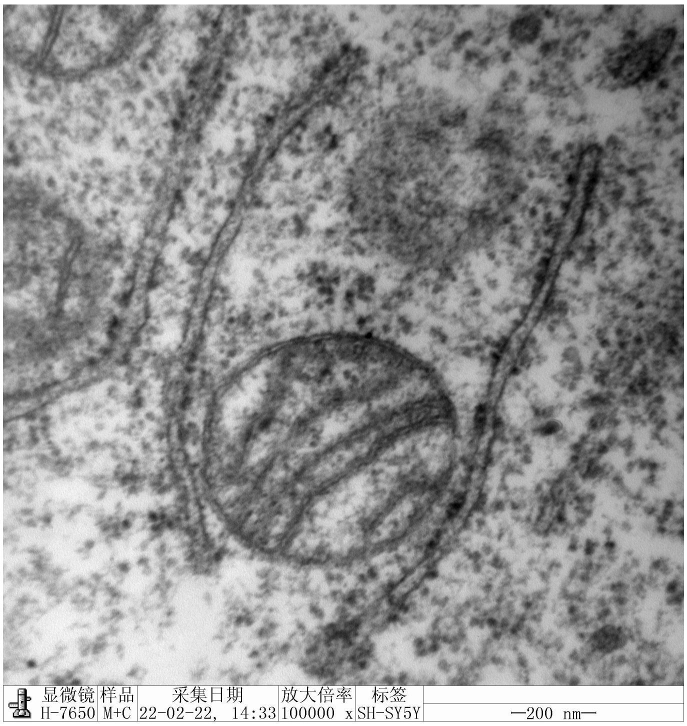
显微镜 样品 采集日期 放大倍率 标签
H-7650 M+C 22-02-22, 14:33 100000 x SH-SY5Y
200 nm

Western blot was used to detect the expression of major proteins in the Ca2+ transport complex

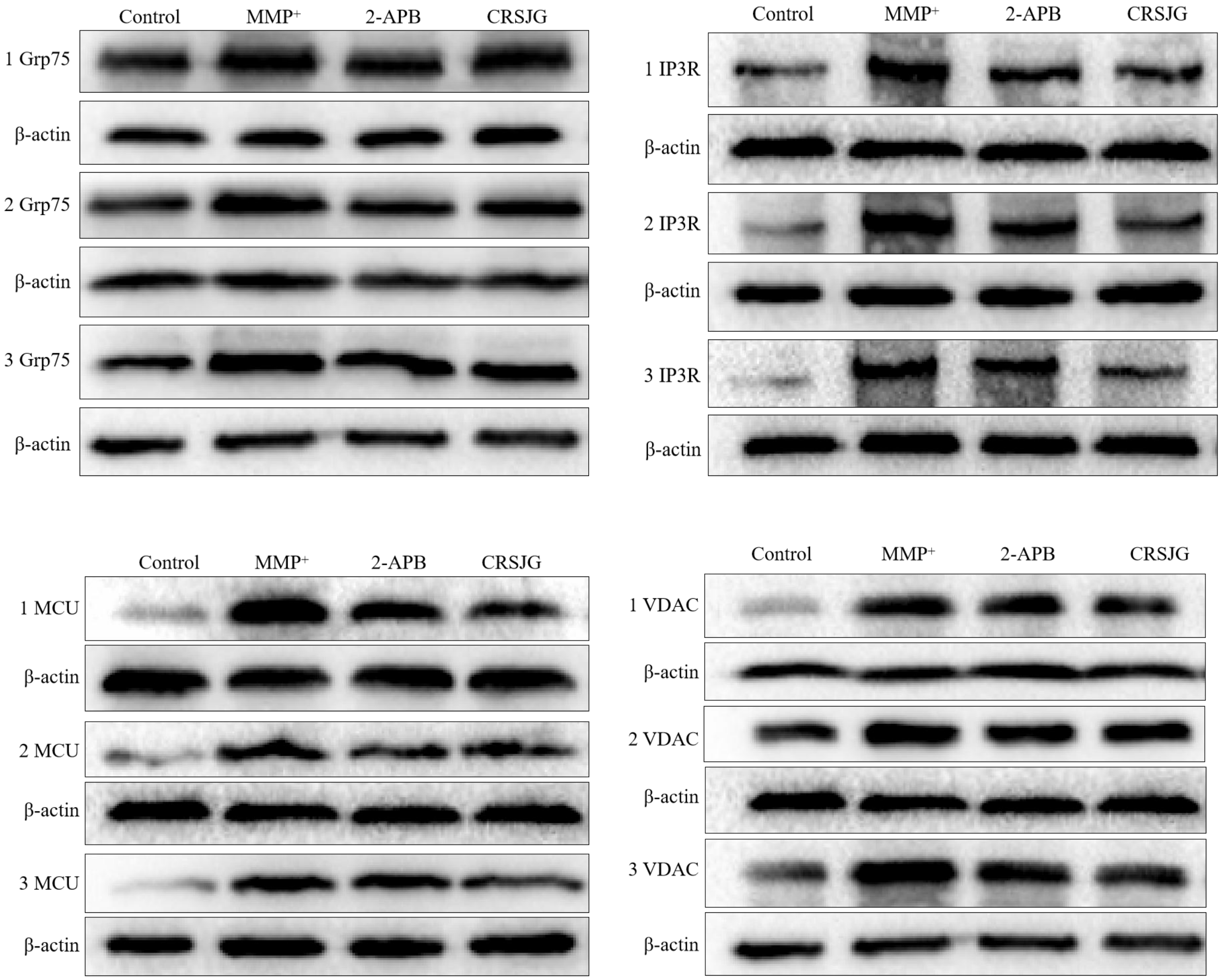

	Target protein（Gray value）			β-actin（Gray value）			ratio		
	sample 1	sample 2	sample 3	sample 1	sample 2	sample 3	sample 1	sample 2	sample 3
IP3R	3303020	1801780	1144550	4867065	2730566	1482532	0.678647193	0.659855869	0.772023808
	12222720	4681596	3073710	5343520	3157308	1584266	2.287391083	1.482780901	1.940147677
	7900928	4217670	2799300	5012315	3081312	1512930	1.576303165	1.368790308	1.850250838
	6794928	3268980	2551050	5005455	3361824	1483552	1.357504563	0.972382849	1.719555499
VDAC	3677990	4244157	5452800	6501088	4867065	6023710	0.565749918	0.872015681	0.905222861
	9692440	8253376	12956346	7172235	5343520	6646252	1.351383495	1.544557894	1.949421418
	7463932	6596512	9079190	7019516	5012315	6589336	1.063311488	1.316060942	1.377861138
	7115622	6677024	7212896	7314109	5005455	6687695	0.97286245	1.333949461	1.078532439
MCU	3133140	1815345	1354848	5150400	4867065	2730566	0.60832945	0.372985567	0.496178448
	6261760	5246774	6254115	4349960	5343520	3157308	1.439498294	0.981894706	1.98083779
	5198668	3558485	5254831	4804454	5012315	3081312	1.082051779	0.709948397	1.70538751
	3933300	3254170	5234500	4953528	5005455	3361824	0.794040127	0.650124714	1.557041654
GRP75	6648480	6381052	5784603	6501088	7538710	6023710	1.022671897	0.846438184	0.960305692
	10130452	11635144	11312828	7172235	8292063	6646252	1.412453998	1.403166377	1.702136482
	9003715	9522903	8765337	7019516	7623168	6589336	1.282668919	1.249205448	1.330230694
	9478560	9763304	8120220	7314109	7852935	6687695	1.295928185	1.243268154	1.2142031
