# Supplementary material for: Congrong Shujing Granules ameliorates mitochondrial associated membranes to against MPP+-induced neurological damage in the cellular model of Parkinson’s disease
Source: Front Pharmacol. 2025 May 30;16:1509317. doi: 10.3389/fphar.2025.1509317 (PMC12162334; doi:10.3389/fphar.2025.1509317)
Supplement: Supplementary file 15 [file DataSheet15.pdf]

# Rhod-2 fluorescence probe was used to detect the mito-$Ca^{2+}$ levels

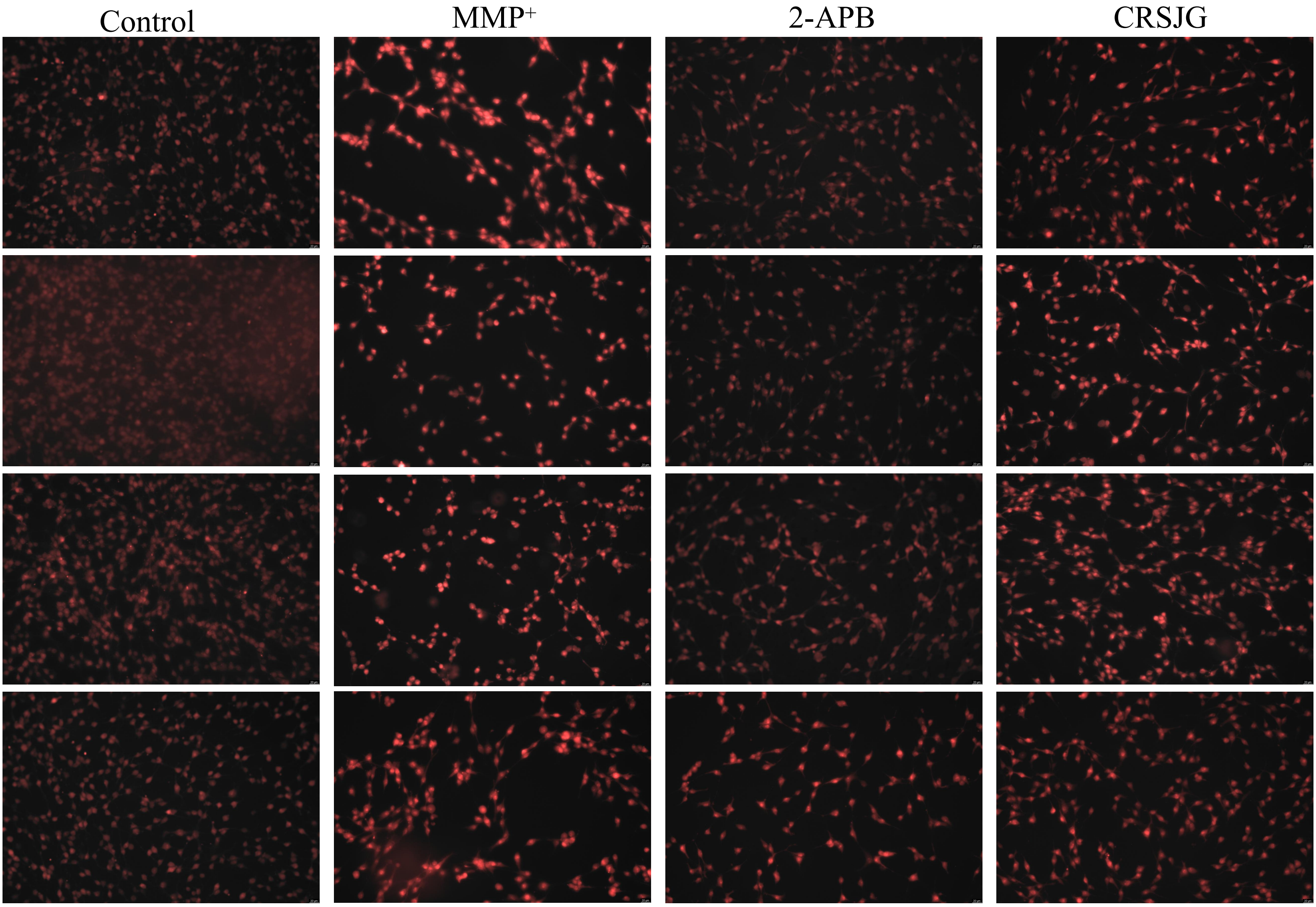


| | Area | Mean | StdDev | IntDen | Median | %Area | RawIntDen | MinThr | MaxThr |
|---|---|---|---|---|---|---|---|---|---|
| Control-1 | 779848 | 47.588 | 7.693 | 37111401 | 46 | 15.627 | 37111401 | 43 | 255 |
| Control-2 | 780563 | 51.739 | 11.732 | 40385278 | 49 | 15.641 | 40385278 | 41 | 255 |
| Control-3 | 1032975 | 42.352 | 13.506 | 43748426 | 40 | 20.699 | 43748426 | 27 | 255 |
| Control-4 | 924087 | 46.545 | 12.977 | 43011899 | 43 | 18.517 | 43011899 | 33 | 255 |
| MPP+-1 | 510366 | 100.214 | 26.344 | 51146060 | 95 | 10.227 | 51146060 | 65 | 255 |
| MPP+-2 | 329478 | 84.95 | 27.859 | 27989108 | 78 | 6.602 | 27989108 | 53 | 255 |
| MPP+-3 | 329283 | 87.718 | 24.194 | 28884055 | 82 | 6.598 | 28884055 | 59 | 255 |
| MPP+-4 | 256778 | 89.862 | 20.505 | 23074659 | 84 | 5.145 | 23074659 | 68 | 255 |
| 2-APB-1 | 496574 | 46.397 | 13.661 | 23039527 | 43 | 9.95 | 23039527 | 33 | 255 |
| 2-APB-2 | 556011 | 50.625 | 13.805 | 28147821 | 47 | 11.141 | 28147821 | 37 | 255 |
| 2-APB-3 | 594203 | 51.117 | 14.333 | 30373792 | 48 | 11.907 | 30373792 | 36 | 255 |
| 2-APB-4 | 338324 | 65.631 | 20.985 | 22204505 | 61 | 6.779 | 22204505 | 42 | 255 |
| CRSJG-1 | 433381 | 63.779 | 21.501 | 27640665 | 58 | 8.684 | 27640665 | 41 | 255 |
| CRSJG-2 | 517422 | 76.063 | 26.408 | 39356420 | 70 | 10.368 | 39356420 | 43 | 255 |
| CRSJG-3 | 679207 | 71.581 | 20.858 | 48618007 | 67 | 13.61 | 48618007 | 47 | 255 |
| CRSJG-4 | 556735 | 59.601 | 18.331 | 33182094 | 55 | 11.156 | 33182094 | 40 | 255 |

# Flou-3 fluorescence probe was used to detect the cyto-$Ca^{2+}$ levels

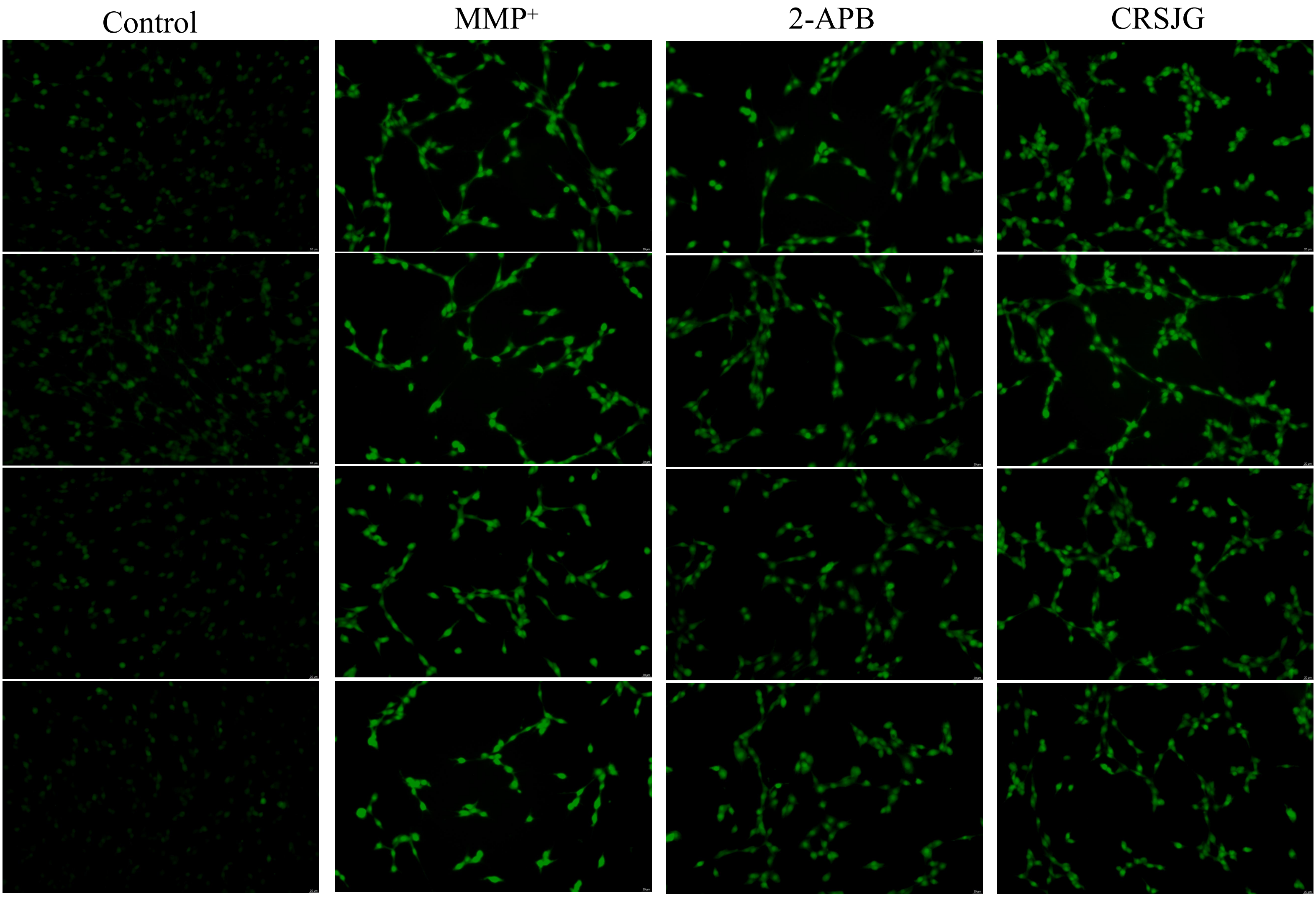


| | Area | Mean | StdDev | IntDen | Median | %Area | RawIntDen | MinThr | MaxThr |
|---|---|---|---|---|---|---|---|---|---|
| Control-1 | 341812 | 12.631 | 9.874 | 4317562 | 11 | 6.849 | 4317562 | 8 | 255 |
| Control-2 | 624782 | 12.91 | 7.788 | 8066185 | 12 | 12.52 | 8066185 | 8 | 255 |
| Control-3 | 264412 | 10.994 | 10.759 | 2906835 | 9 | 5.298 | 2906835 | 7 | 255 |
| Control-4 | 227286 | 8.774 | 11.773 | 1994171 | 7 | 4.554 | 1994171 | 5 | 255 |
| MPP+-1 | 289386 | 33.182 | 12.102 | 9602329 | 31 | 5.799 | 9602329 | 21 | 255 |
| MPP+-2 | 268993 | 38.408 | 12.884 | 10331552 | 40 | 5.39 | 10331552 | 21 | 255 |
| MPP+-3 | 334555 | 34.415 | 11.224 | 11513582 | 33 | 6.704 | 11513582 | 23 | 255 |
| MPP+-4 | 338176 | 35.841 | 12.395 | 12120726 | 35 | 6.776 | 12120726 | 20 | 255 |
| 2-APB-1 | 400358 | 25.82 | 10.617 | 10337224 | 24 | 8.022 | 10337224 | 16 | 255 |
| 2-APB-2 | 343233 | 24.168 | 11.261 | 8295269 | 22 | 6.878 | 8295269 | 15 | 255 |
| 2-APB-3 | 331894 | 27.007 | 11.322 | 8963614 | 25 | 6.651 | 8963614 | 17 | 255 |
| 2-APB-4 | 534530 | 24.736 | 12.77 | 13222290 | 22 | 10.711 | 13222290 | 11 | 255 |
| CRSJG-1 | 514558 | 30.565 | 11.578 | 15727423 | 29 | 10.311 | 15727423 | 17 | 255 |
| CRSJG-2 | 405326 | 28.433 | 10.921 | 11524520 | 27 | 8.122 | 11524520 | 18 | 255 |
| CRSJG-3 | 320950 | 28.707 | 11.657 | 9213610 | 27 | 6.431 | 9213610 | 18 | 255 |
| CRSJG-4 | 528218 | 31.071 | 10.184 | 16412080 | 30 | 10.585 | 16412080 | 20 | 255 |

**The microscopic structure of MAMS was observed by transmission electron microscope, magnification (×20000), magnification of red rectangle box (×100000)**

Control

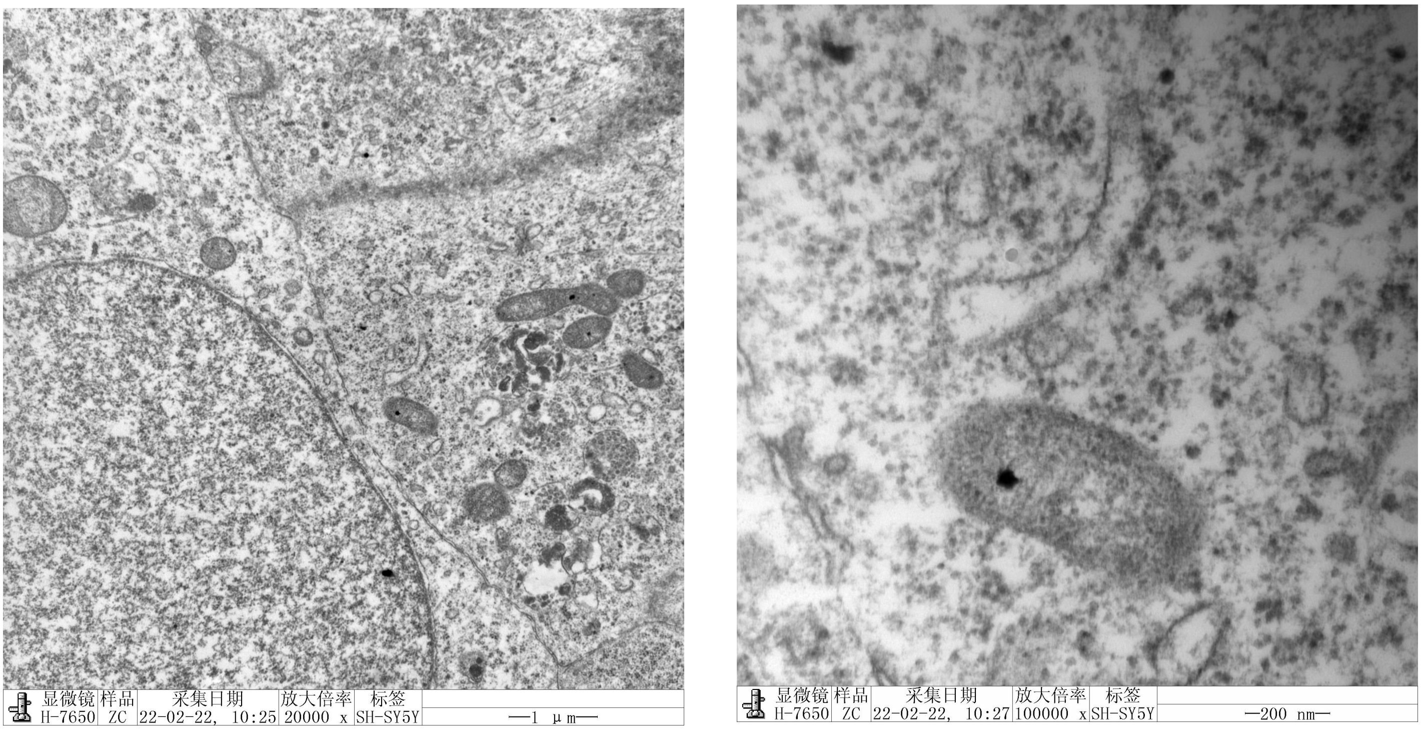


MPP+

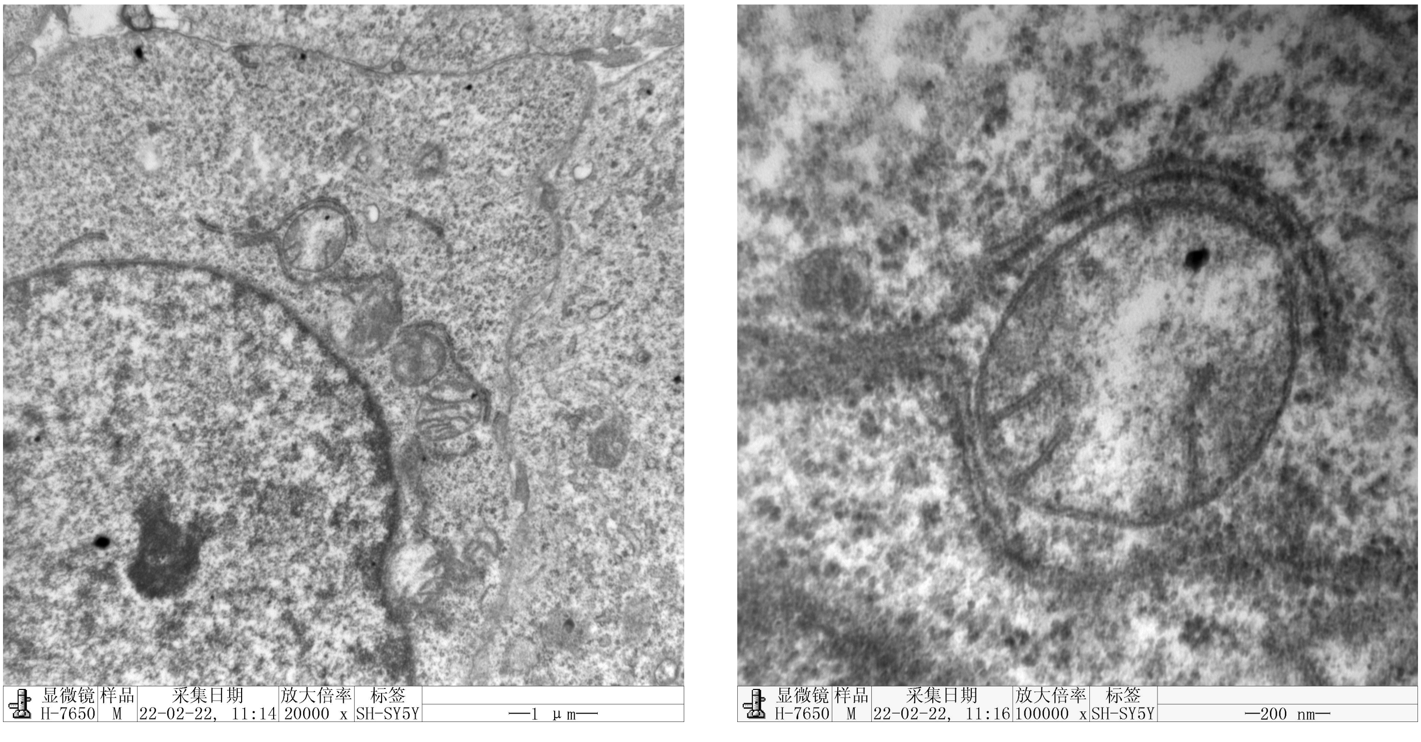

# 2-APB

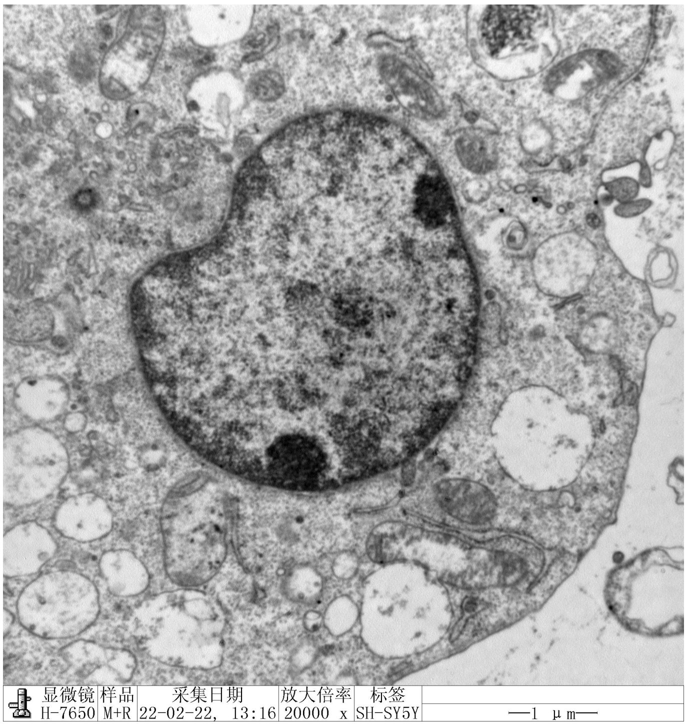
显微镜 样品 采集日期 放大倍率 标签
H-7650 M+R 22-02-22, 13:16 20000 x SH-SY5Y
1 μm

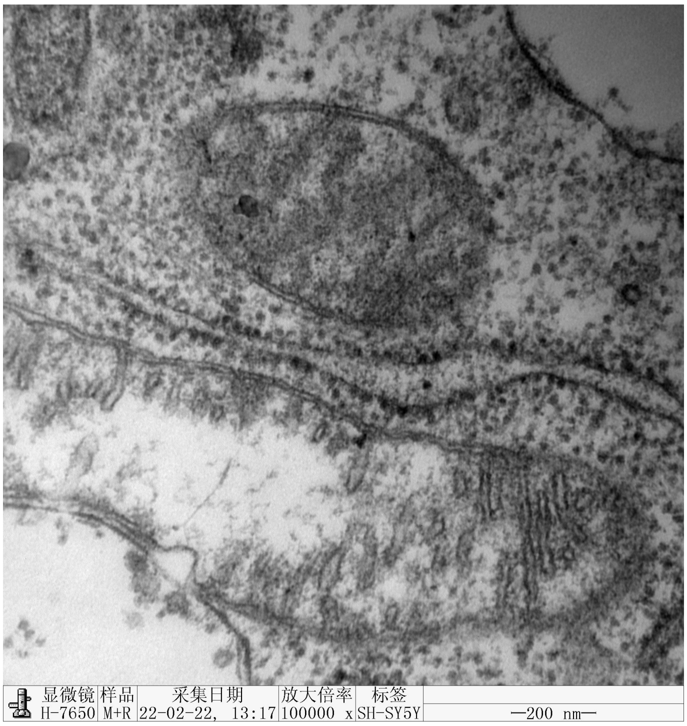
显微镜 样品 采集日期 放大倍率 标签
H-7650 M+R 22-02-22, 13:17 100000 x SH-SY5Y
200 nm

# CRSJG

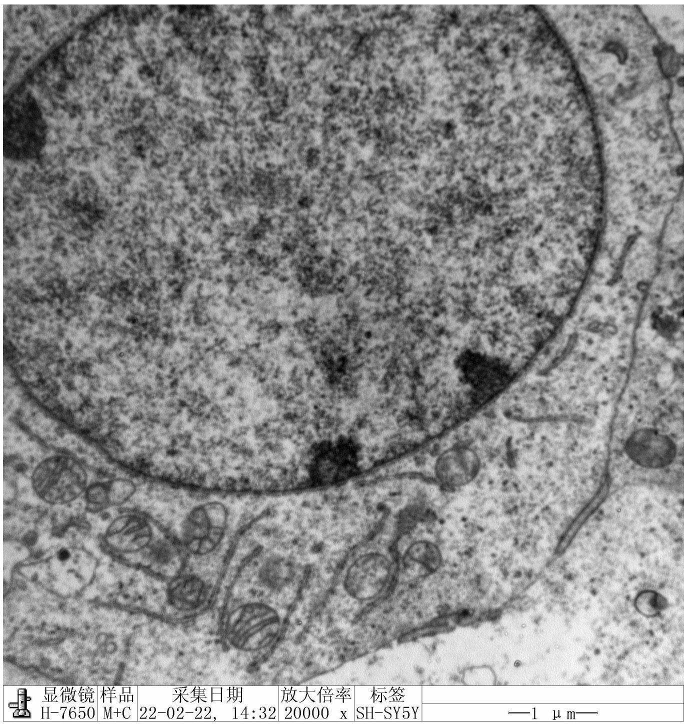
显微镜 样品 采集日期 放大倍率 标签
H-7650 M+C 22-02-22, 14:32 20000 x SH-SY5Y
1 μm

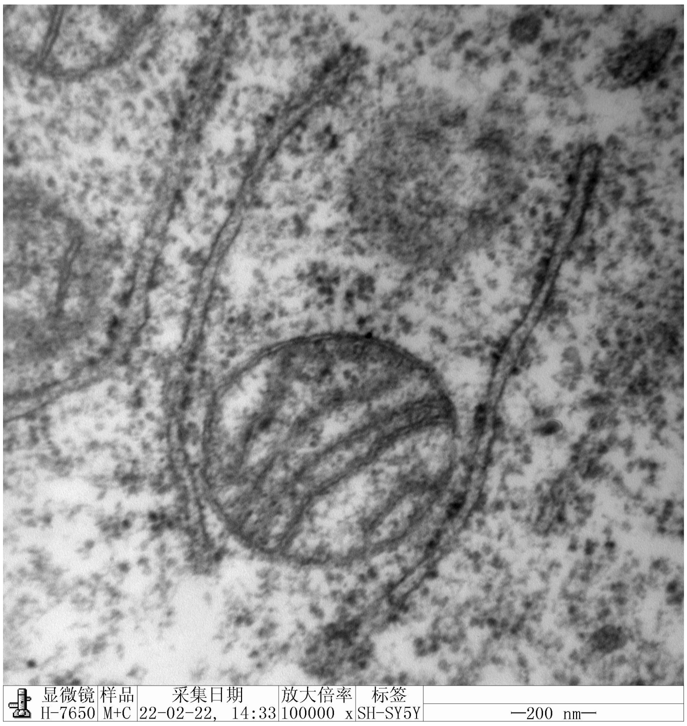
显微镜 样品 采集日期 放大倍率 标签
H-7650 M+C 22-02-22, 14:33 100000 x SH-SY5Y
200 nm
